# Cucurbitane Glycosides from *Siraitia Grosvenorii* and Their Hepatoprotective Activities

**DOI:** 10.3390/molecules30193983

**Published:** 2025-10-04

**Authors:** Jia-Nan Mao, Hua-Xue Huang, Qing-Ling Xie, Guang-Yu Chen, Juan-Jiang Wu, Ying Deng, Shuang Zhan, Zhi Peng, Xu-Dong Zhou, Wei Wang

**Affiliations:** 1 TCM and Ethnomedicine Innovation & Development International Laboratory, Innovative Materia Medica Research Institute, School of Pharmacy, Hunan University of Chinese Medicine, Changsha 410208, China; 19313189907@139.com (J.-N.M.); river@huachengbio.com (H.-X.H.); xieql12@126.com (Q.-L.X.); 004581@hnucm.edu.cn (G.-Y.C.); 3230004587@student.must.edu.mo (J.-J.W.); dengying028@163.com (Y.D.); 2 Modernization Industry College for Innovative Chinese Medicine, Hunan University of Chinese Medicine, Changsha 410208, China; 3 Faculty of Chinese Medicine, Macau University of Science and Technology, Macau SAR 999078, China; 4 Research and Development Institute of Hunan Huacheng Biotech, Inc., Changsha 410205, China; support@huachengbio.com (S.Z.); pengzhi3778@163.com (Z.P.); 5 Hunan Natural Sweetener Engineering Technology Research Center, Changsha 410205, China

**Keywords:** *Siraitia grosvenorii*, Luohanguo, cucurbitane-type triterpenoid glycosides, hepatoprotective activities

## Abstract

*Siraitia grosvenorii* (*S. grosvenorii*), a traditional medicine food homology plant, serves both dietary and medicinal purposes and is increasingly exploited for its bioactivities in pharmaceuticals and nutritional value. In this research, fifteen glycosides including three new cucurbitane-type triterpenoid glycosides named Luohanguosides A–C (**1**–**3**) and twelve known ones (**4**–**15**) have been isolated from the aqueous extract of fresh *S. grosvenorii* fruits. A comprehensive analysis of 1D, 2D-NMR, HRESIMS techniques along with some other spectroscopic methods led to the elucidation of their chemical structures. Further investigation focused on the hepatoprotective activities of compounds **1**–**15**. It turned out that compounds **1**, **5,** and **10** exhibited significant hepatoprotective activities compared to bicyclol under the same concentration (20 μM), providing scientific support for further research on *S.grosvenorii* products for their preventive potential of hepatic diseases.

## 1. Introduction

*Siraitia grosvenorii* (*S. grosvenorii*) [Swingle] C. Jeffrey ex A. M. Lu et Z. Y. Zhang, commonly known as Luohanguo or monk fruit belonging to the Cucurbitaceae family, is a traditional Chinese medicine (TCM) native to South China [1]. The traditional use of *S. grosvenorii* mainly consists of clearing heat and moistening the lungs, resolving phlegm and relieving cough, and alleviating throat discomfort [2]. Based on modern research, more than 200 constituents have been found from *S. grosvenorii* including triterpenoid glycosides, flavonoids, amino acids, and polysaccharides. The main active components of this plant have been regarded as mogrosides, such as mogroside V, mogroside IIIE, and mogroside VI, which were identified as cucurbitane-type triterpenoid glycosides characterized by glycosidic linkage. Till now, no more than 50 cucurbitane glycosides have been isolated and identified from *S. grosvenorii*, rendering the in-depth research on the material basis of this plant meaningful [3]. As a natural sweetening agent, mogrosides were not only listed as a kind of food additive which can be used in various food products according to the requirements of specific groups for high sweetness, zero calories, and dietary health [4,5,6], but also widely investigated and applied mainly due to its multiple bioactivities and strong potential in the natural sweetener market [7].

As one of the first approved medicine food homology (MFH) species, *S. grosvenorii* has been proven to have a variety of pharmacological properties such as containing antioxidant abilities [8], anti-inflammatory effects [9,10], hepatoprotective properties [11,12], antidiabetic effects [13,14], regulation of sugar and lipid metabolism properties [15] and anti-cancer effects [16]. Accordingly, the bioactivity of mogrosides contributes to their nutritional value, including natural sweetness with low calories, a low glycemic index, antioxidants, anti-inflammatory effects, lipid-regulating properties, and beneficial bacteria enrichment, making it possible for mogrosides to become a valuable natural food additive [6,17].

Mogrosides have been suggested to have protective abilities for hepatic injury through different mechanisms such as improving the detoxification function of the liver, reducing liver injury, promoting the repair and regeneration of liver cells, antioxidation and anti-inflammation [11,12]. Furthermore, based on our years of investigation on traditional Chinese medicines along with their multiple bioactivities, especially hepatoprotective properties [18,19], a hepatoprotective experiment was performed on the glycosides isolated from the *S. grosvenorii* fruit.

## 2. Results and Discussion

**Figure 1:**
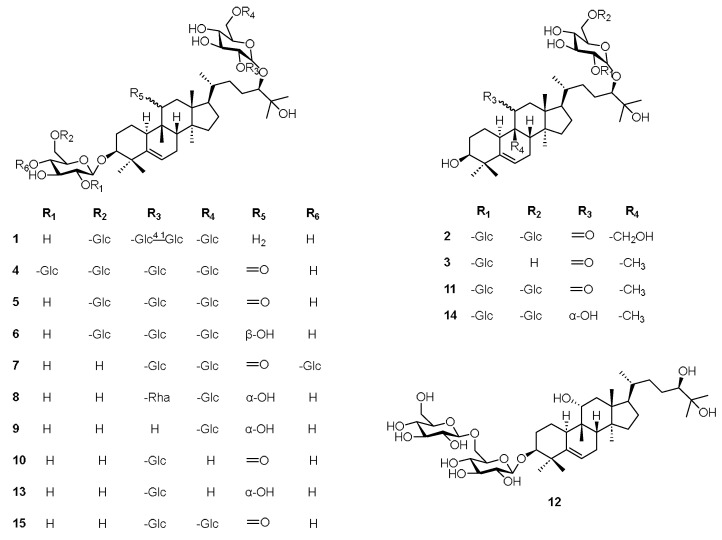
Chemical structures of compounds **1**–**15**.

**Figure 2:**
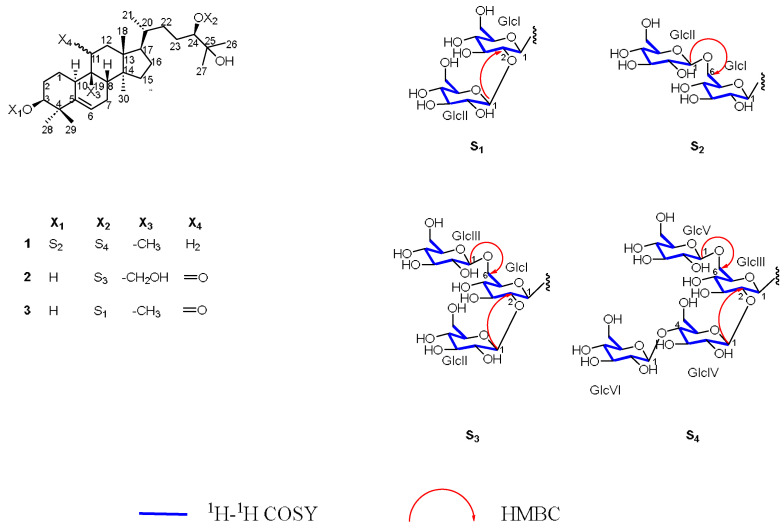
Structures and key 2D-NMR correlations of compounds **1**–**3**.

The study resulted in the isolation of fifteen compounds (**1**–**15**), including three new cucurbitane-type triterpenoid glycosides (**1**–**3**), and their structures elucidated through various spectroscopic approaches (see Figure 1 and Figure 2). The hydrolyzed sugars of new compounds were derivatized by the use of L-cycteinemethyl ester and isothiocyanate. Upon hydrolysis of glycosides, only one sugar was observed as product, and it was D-glucose.

The structures of known compounds (**4**–**15**) were indicated by a comprehensive analysis of 1D and 2D-NMR spectra. Additionally, compared with reported NMR and HRESIMS data, they were determined as 11-oxo-mogroside VI (**4**) [20], 11-oxo-mogroside V (**5**) [21], 11-epi-mogroside V (**6**) [20], 11-oxoisomogrosideV (**7**) [18], (3β,9β,10α,11α,24R)-3-(β-D-Glucopyranosyloxy)-11,25-dihydroxy-9-methyl-19-norlanost-5-en-24-yl O-6-deoxy-α-L-mannopyranosyl-(1→2)-O-[β-D-glucopyranosyl-(1→6)]-β-D-glucopyranoside (**8**) [20], mogroside III A2 (**9**) [22], 11-oxomogroside III E (**10**) [23], 11-oxomogroside III A1 (**11**) [24], mogroside II A2 (**12**) [25], mogroside III (**13**) [26], mogroside III A1 (**14**) [20], 11-oxo-siamenoside I (**15**) [20], respectively (see Figure 2). Afterwards, compounds **1**–**15** were evaluated for their hepatoprotective effects.

### 2.1. Detailed Information and Elucidation for New Compounds

Compound **1** was obtained as a white amorphous powder. The molecular formula C_66_H_112_O_33_ was established by positive and negative ion mode HRESIMS, which showed prominent peaks at *m*/*z* 1471.6796 [M + K]^+^ (calcd. for C_66_H_112_O_33_K^+^, 1471.6717) and 1431.7014 [M – H]^−^ (calcd. for C_66_H_111_O_33_^−^, 1431.7013). The low field region of ^1^H NMR and ^13^C NMR spectra displayed six groups of anomeric signals at δ_H_ 4.80 (1H, d, *J* = 7.5 Hz) / δ_C_ 107.0; δ_H_ 4.87 (1H, d, *J* = 7.6 Hz) / δ_C_ 104.9; δ_H_ 4.93 (1H, d, *J* = 7.6 Hz) / δ_C_ 103.7; δ_H_ 5.13 (1H, d, *J* = 7.9 Hz) / δ_C_ 105.5; δ_H_ 5.19 (1H, d, *J* = 7.8 Hz) / δ_C_ 105.4 and δ_H_ 5.44 (1H, d, *J* = 7.9 Hz) / δ_C_ 105.3 (see Table 1), providing evidence of two set of glycosides, one with two sugars and the other with four sugars, with six sugars overall (see Table 2). Additionally, the high-field region of ^1^H NMR and ^13^C NMR exhibited eight groups of methyl signals including δ_H_ 0.95 (3H, s) / δ_C_ 17.1; δ_H_ 1.52 (3H, s) / δ_C_ 26.3; δ_H_ 1.12 (3H, d, *J* = 6.4 Hz) / δ_C_ 19.1; δ_H_ 1.43 (3H, s) / δ_C_ 24.6; δ_H_ 1.34 (3H, s) / δ_C_ 27.0; δ_H_ 1.17 (3H, s) / δ_C_ 27.9; δ_H_ 1.52 (3H, s) / δ_C_ 26.3 and δ_H_ 0.94 (3H, s) / δ_C_ 19.4; nine groups of methylene signals including δ_H_ 2.02 (1H, m) and 3.00 (1H, m) / δ_C_ 26.9; δ_H_ 2.23 (1H, m) and 2.52 (1H, m) / δ_C_ 29.5; δ_H_ 1.69 (1H, m) and 2.30 (1H, m) / δ_C_ 24.6; δ_H_ 1.46 (1H, m) and 2.12 (1H, m) / δ_C_ 28.6; δ_H_ 1.08 (1H, m) and 1.15 (1H, m) / δ_C_ 34.6; δ_H_ 2.16 (1H, m) and 2.21 (1H, m) / δ_C_ 41.1; δ_H_ 1.46 (1H, m) and 2.12 (1H, m) / δ_C_ 28.4; δ_H_ 1.79 (1H, m) and 1.87 (1H, m) / δ_C_ 33.3; δ_H_ 1.58 (1H, m) and 1.89 (1H, m) / δ_C_ 29.8; along with six methine signals δ_H_ 3.69 (1H, br, s) / δ_C_ 87.6; δ_H_ 1.66 (1H, m) / δ_C_ 43.6; δ_H_ 2.85 (1H, d, *J* = 12.2) / δ_C_ 36.7; δ_H_ 1.79 (1H, m) / δ_C_ 51.2; δ_H_ 1.53 (1H, m) / δ_C_ 36.6; δ_H_ 3.77 (1H, m) / δ_C_ 92.3; and one olefinic proton signal at δ_H_ 5.49 (1H, m) / δ_C_ 118.3. Moreover, ^13^C NMR spectra showed five quaternary carbon signals at δ_C_ 40.2, 42.4, 49.7, 47.5, 72.8 and one olefinic carbon signal at δ_C_ 144.4 (see Table 1). A comprehensive analysis of ^1^H-^1^H COSY, HMBC, HSQC and NOESY spectra contributed to converting these fragments into four directly connected rings, deducing the aglycone of compound **1** as a triterpenoid with a cucurbitane-type skeleton. Furthermore, all the ^13^C NMR chemical shifts of aglycone were numerically close to that of 11-deoxymogroside V [27], deducing the aglycone as 11-deooxy-mogrol.

Upon hydrolysis of glycosides, six sugar moieties of single configuration D-glucose were obtained. NOESY spectra along with the coupling constant of anomeric protons ranging from 7.5 to 7.9 identified the sugars as β-configurations of these anomeric carbons. In addition, according to HMBC, correlation signals could be found between δ_H_ 4.80 (1H, d, *J* = 7.5 Hz) with δ_C_ 87.6; between δ_H_ 4.93 (1H, d, *J* = 7.6 Hz), with δ_C_ 92.3, elaborating that GlcI and GlcIII were separately connected to C-3 and C-24 of aglycone. Some key HMBC cross-peaks could also be found between δ_H_ 5.19 (1H, d, *J* = 7.8 Hz) with δ_C_ 70.3; between δ_H_ 5.44 (1H, d, *J* = 7.9 Hz) with δ_C_ 82.6; between δ_H_ 4.87 (1H, d, *J* = 7.6 Hz) with δ_C_ 70.2; between δ_H_ 5.13 (1H, d, *J* = 7.9 Hz) wtih δ_C_ 82.4 (see Table 2). By ^1^H-^1^H COSY, HSQC, HMBC, NOESY spectrum, combined with TOCSY spectra analysis, all these ^13^C NMR signals were elucidated as C-6 of GlcI, C-2 of GlcIII, C-6 of GlcIII, and C-4 of GlcIV, respectively, demonstrating that GlcII was attached to C-6 of GlcI, GlcIV and V were separately connected to C-2 and C-6 of GlcIII while GlcVI was connected to C-4 of GlcIV (see Figure 1 and Figure 2). Thus, the structure of **1** was defined, named Luohanguonoside A.

Compound **2** was identified as a white amorphous solid; the molecular ion in HRESIMS at *m*/*z* 999.5125 [M + Na]^+^ (calcd. for C_48_H_80_O_20_Na^+^, 999.5135) and 977.5306 [M + H]^+^ (calcd. for C_48_H_81_O_20_^+^, 977.5316) determined the molecular formula of **2** as C_48_H_80_O_20_. The ^1^H NMR and ^13^C NMR exhibited three anomeric signals at δ_H_ 4.88 (1H, d, *J* = 7.7 Hz) / δ_C_ 104.9; δ_H_ 4.93 (1H, d, *J* = 7.5 Hz) / δ_C_ 103.7 and δ_H_ 5.54 (1H, d, *J* = 7.8 Hz) / δ_C_ 105.5, indicating three sugar residues were attached to the aglycone of **2** (see Table 2). The ^1^H NMR and ^13^C NMR data of the aglycone were similar to those of 11-oxo-mogrol except for an oxidized methylene at δ_C_ 60.4 replacing the ^13^C NMR of C-19 methyl; for that, H-8 (1H, m) exhibited a long-range correlation to δ_C_ 60.4 in HMBC [28] (see Table 1). Combined with the fully comprehensive analysis of the 1D and 2D-NMR spectrum, the structure of aglycone was deduced as 11-oxo-mogrol oxidized at C-19.

The acid hydrolysis experiment contributed to identifying the sugar moieties as D-glucose. NOESY data and coupling constants of anomeric protons elucidated that the glucose adopted β-configurations of anomeric center. Accordingly, glycosidated C-24 at δ_C_ 92.2 to which δ_H_ 4.93 (1H, d, *J* = 7.5 Hz) was correlated through HMBC showed that GlcI was connected to C-24. In HMBC, long-range correlation existed between δ_H_ 5.54 (1H, d, *J* = 7.8 Hz), δ_C_ 82.2, δ_H_ 4.88 (1H, d, *J* = 7.7 Hz), and δ_C_ 70.2, identified as C-2 and C-6 of GlcI, illustrating that GlcII and III were connected to C-2 and C-6 of GlcI. In addition, all the ^1^H and ^13^C NMR chemical shifts were almost the same to those of 11-oxo-mogroside IIIA_1_ [24] with the exception of an oxidized methylene, providing evidence for the structural elucidation of compound **2**, subsequently being named Luohanguonoside B (see Figure 1 and Figure 2).

Compound **3** was identified as a white amorphous solid. HRESIMS provided molecular ions at *m*/*z* 821.4637 [M + Na]^+^ (calcd. for C_42_H_70_O_14_Na^+^, 821.4658) and 799.4823 [M + H]^+^ (calcd. for C_42_H_71_O_14_^+^, 799.4838), determining the molecular formula as C_42_H_70_O_14_. In the ^1^H NMR and ^13^C NMR spectrum, two groups of anomeric signals at δ_H_ 5.08 (1H, d, overlap) / δ_C_ 102.2 and δ_H_ 5.40 (1H, d, *J* = 7.7 Hz) / δ_C_ 106.6 appeared in the low-field region, suggesting **3** possessed two sugars moieties (see Table 2). Furthermore, aglycone was elucidated as 11-oxo-mogrol compared to reported data combined with ^1^H-^1^H COSY, HSQC, HMBC and NOESY spectra analysis [28] (see Table 1).

Acid hydrolysis revealed the sugar units as D-glucose, and the β-configuration of anomeric centers of both glucoses were established from NOESY correlations and the anomeric proton coupling constants. In addition, C-24 at δ_C_ 88.4 had a glycosylated-like shift and it was connected with GlcI for that δ_H_ 5.08 (1H, d, overlapped) was distantly correlated to C-24 in HMBC. Another anomeric hydrogen at δ_H_ 5.40 (1H, d, *J* = 7.7 Hz) exhibited a long-range relationship with δ_C_ 84.1, which was identified as C-2 of GlcI by comprehensively analyzing 2D-NMR, suggesting that GlcII was attached to C-2 of GlcI, named Luohanguonoside C (see Figure 1 and Figure 2).

### 2.2. Cytotoxicity and Hepatoprotective Activity

**Figure 3:**
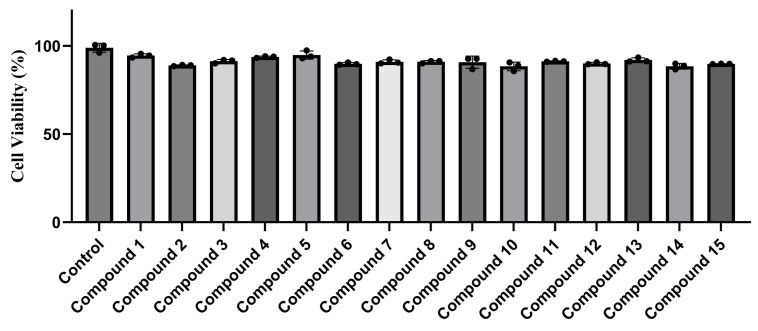
Cytotoxicity of compounds **1**–**15** (20 µM) to ALM-12 cells. Data are presented with mean± SD (n = 3).

**Figure 4:**
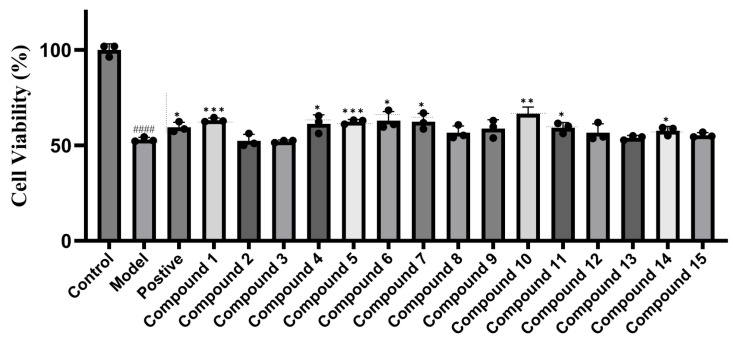
Hepatoprotective activity of compounds **1**–**15** (20 µM) on ALM-12 cells. Data are shown as mean± SD (n = 3). Details: Bicyclol was used as positive control and H_2_O_2_ was used to cause liver damage model. Compared with control group: #### *p* < 0.0001, compared with model group: *** *p* < 0.001, ** *p* < 0.01, * *p* < 0.05.

A cytotoxic experiment was conducted to evaluate the influence of compounds **1**–**15** on ALM-12 cells with the cell viability ranging from 88.43% ± 2.46% to 94.85% ± 2.29% (control group, 98.96% ± 2.48%) (see Figure 3), indicating that **1**–**15** had no evident cytotoxicity against ALM-12 cells, which contributed to a further study on the assessment of hepatoprotective activity of these compounds. Subsequently, a H_2_O_2_-induced hepatic injury model was performed on ALM-12 cells which have been treated with compounds **1**–**15** (20 µM) to evaluate their protective effects, using bicyclol (20 µM) as positive comparison. It turned out that compounds **1**, **5**, and **10** exhibited evident liver protective efficiency with a cell viability of 63.20% ± 1.11%, 62.32% ± 1.18%, and 66.52% ± 3.52%, respectively, while compounds **4**, **6**, **7**, **11** and **14** showed apparent cell viability improvement in 61.29% ± 4.71%, 62.92% ± 4.73%, 58.79% ± 4.60%, 59.21% ± 2.72%, and 57.64% ± 2.30%, respectively, compared with that of model group. (control group, 100.00% ± 3.20%; H_2_O_2_ group, 52.98% ± 1.16%; bicyclol group, 59.41% ± 2.67%) (see Figure 4).

## 3. Materials and Methods

### 3.1. General Experimental Procedures

^1^H and ^13^C NMR spectra data (Figures S1–S8) were obtained at 600 and 150 MHz, respectively, on a Bruker Avance III HD spectrophotometer (Bruker Co., Karlsruhe, Germany). Compounds were dissolved in C_5_D_5_N. Optical rotation measurement for compounds was performed on a Perkin-Elmer 341-MC polarimeter (PerkinElmer, Waltham, MA, USA). HRESIMS data were acquired on a Vanquish Flex Binary/Orbitrap Exploris 120 UHPLCHRMS spectrometer (Thermo Fisher Scientific, Corporation, Waltham, MA, USA). UV–visible data were acquired on a Shimadzu 2450 UV–vis spectrophotometer (Shimadzu Corporation, Kyoto, Janpan) while FTIR data was confirmed (both in methanol) on a Cary 630 Agilent spectrophotometer (Agilent Technologies Corporation, Santa Clara, CA, USA). The glycohydrolytic experiment was performed on a 5 μm 4.6 mm × 250 mm C_18_ column (Agilent Technologies Corporation, Santa Clara, CA, USA) with 20–35% CH_3_CN in 0.1% CH_3_COOH-H_2_O analytically pure to provide auxiliary evidence for the types of sugars. The purification of compounds was by use of silica gel with pore size of 80–100 and 200–300 mesh (Qingdao Marine Chemical Branch Factory, Qingdao, China), MCI gels (Mitsubishi chemical corporation, Kyoto, Janpan) along with semi-preparative HPLC (SEP Beijing Technology Corporation, Beijing, China). The C_18_ column (250 × 10 mm D. S-5 μm, 12 nm (SilGreen, Beijing Technology Corporation, Beijing, China) was performed on HPLC. The chromatographic grade methanol (Merck KgaA Corporation, Darmstadt, Germany), was used for HPLC experiment. Cell counting kit-8 (CCK-8) detection reagent (Elabscience Biotechnology Corporation, Wuhan, China) and dimethyl sulfoxide (DMSO) (Sinopharm Chemical ReagentCorporation, Shanghai, China) were performed in biological detection. The bicyclol purchased from National Institutes for Food and Drug Control (NIFDC) was used as a positive drug in the bioassay of hepatoprotective activities. The bioassay data were acquired by an enzyme-linked immunosorbent assay (ELISA) reader (Agilent technologiesCorporation, Santa Clara, CA, USA). Alpha mouse liver 12 (ALM-12) cells and ALM-12 cells specialized medium (DMEM/F12) were obtained from Wuhan Pricella Biotechnology Corporation (Wuhan, China).

### 3.2. Plant Material

The aqueous crude extract of fresh *S. grosvenorii* fruit was provided by Hunan Huacheng Biotech, Inc (Changsha, China). The source herb, obtained from Liuzhou, Guangxi (latitude 25°12′, longitude 109°29′), was identified by Professor Wei Wang (Hunan University of Chinese Medicine).

### 3.3. Isolation and Purification

The aqueous crude extract of *S. grosvenorii* (1.0 kg) was dissolved into water and then absorbed on a D101 microporous adsorption resin (12.0 kg; column, 35 cm × 100 cm) and eluted with 100% H_2_O, 20%, 70%, and 95% EtOH in H_2_O, respectively. The 70% eluate was under reduced pressure using a rotary evaporator, obtaining dried crude saponin powder (362 g). Subsequently, the crude saponin was separated by silica gel column chromatography with the gradient solvent systems of CH_2_Cl_2_: EtOH: H_2_O (3:1:1, 4:2:1, 5:3:1) and followed with *n*-BuOH: EtOH: H_2_O (25:5:1, 16:4:1, 9:3:1, 4:2:1) to obtain ten fractions (Fr.1–10). The subfraction Fr.4 was continuously purified with a semi-preparative HPLC (3.0 mL/min, MeOH-H_2_O, 11:25 and 3:5) to afford **9** (4.8 mg), **10** (3.0 mg), and **11** (10.5 mg). In the same manner, the semi-preparative HPLC (3.0 mL/min, MeOH-H_2_O, 9:20) was also used on the isolation of Fr.5 to afford **8** (10.7 mg) and 13 (12.0 mg). Meanwhile, Fr.6 was further isolated with 55% and 72% MeOH in H_2_O to obtain **3** (4.3 mg). Fr.7 was purified with 32%, 65% and 75% MeOH in H_2_O on the semi-preparative HPLC to obtain **2** (2.3 mg), **12** (3.0 mg) and **14** (16.3 mg). Similarly, Fr.8 was performed on the semi-preparative HPLC eluting with MeOH/H_2_O (3.0 mL/min, 52:100 and 57:100) to yield **15** (12.2 mg) while Fr.10 was primarily purified with an MCI gel column eluted with MeOH/H_2_O (0.1:1–1:1). Afterwards, the 40% MeOH/H_2_O eluate was performed on semi-preparative HPLC (3.0 mL/min, MeOH-H_2_O, 9:20 and 49:100), affording **4** (19.7 mg), **1** (24.1 mg), **6** (14.9 mg), and **7** (17.9 mg). The 50% MeOH/H_2_O fraction was also purified by the use of the same semi-preparative HPLC (3.0 mL/min, MeOH-H_2_O, 51:100) to obtain **5** (21.0 mg).

#### 3.3.1. Luohanguoside A (**1**)

White amorphous solid; HRESIMS *m*/*z* 1471.6796 [M + K]^+^ (calcd. for C_66_H_112_O_33_K^+^, 1471.6717) 1431.7014 [M – H]^–^ (calcd. for C_66_H_111_O_33_^–^, 1431.7013); UV (MeOH) *λ*_max_ (log *ε*): 202 (3.87) nm; IR ν_max_: 3302, 2944, 2832, 1450, 1113, 1024 cm^−1^; $[{\alpha ]}_{D}^{25}$ − 17.0 (*c* 0.10, MeOH); ^1^H and ^13^C NMR signals information—see Table 1 and Table 2**.**

#### 3.3.2. Luohanguoside B (**2**)

White amorphous solid; HRESIMS *m*/*z* 999.5125 [M + Na]^+^ (calcd. for C_48_H_80_O_20_Na^+^, 999.5135) 977.5306 [M + H]^+^ (calcd. for C_48_H_81_O_20_^+^, 977.5316); UV (MeOH) *λ*_max_ (log *ε*): 202 (3.82) nm; IR ν_max_: 3327, 2943, 2832, 1654, 1455, 1116, 1023 cm^−1^; $[{\alpha ]}_{D}^{25}$ + 18.9 (*c* 0.07, MeOH); ^1^H and ^13^C NMR signals information—see Table 1 and Table 2.

#### 3.3.3. Luohanguoside C (**3**)

White amorphous solid; HRESIMS *m*/*z* 821.4637 [M + Na]^+^ (calcd. for C_42_H_70_O_14_Na^+^, 821.4658) 799.4823 [M + H]^+^ (calcd. for C_42_H_71_O_14_^+^, 799.4838); UV (MeOH) *λ*_max_ (log *ε*): 201 (3.78) nm; IR ν_max_: 3304, 2943, 2833, 1655, 1448, 1116, 1022 cm^−1^; $[{\alpha ]}_{D}^{25}$ + 49.2 (*c* 0.07, MeOH); ^1^H and ^13^C NMR signals information—Table 1 and Table 2.

### 3.4. Acid Hydrolysis

Compounds **1**–**3** (1 mg each) were subjected to acid hydrolysis with 2 M HCl (10 mL) at 80 °C under reflux for 5 h. After cooling, the hydrolysates were concentrated to dryness in vacuo, redissolved in H_2_O (5 mL), and extracted with EtOAc (3 × 5 mL). The aqueous layers were again concentrated to dryness, then dissolved into dry pyridine (1 mL). The sugars were derivatized sequentially with L-cysteine methyl ester hydrochloride (2 mg, 60 °C, 1 h) and phenyl isothiocyanate (2 mg, 60 °C, 1 h). The resulting thiazolidine derivatives were analyzed by analytical HPLC. Co-injection with authentic standards showed a single peak for D-glucose (*t*_R_ 13.74–13.84 min) in every hydrolysate, whereas L-glucose eluted at *t*_R_ 13.22 min under identical conditions. These data confirm that D-glucose is the sole monosaccharide constituent of compounds **1**–**3**.

### 3.5. Cytotoxicity Assay

ALM-12 cells were maintained in Dulbecco’s Modified Eagle Medium/Nutrient Mixture F-12 (DMEM/F-12) supplemented with 10% fetal bovine serum and 1% penicillin-streptomycin under standard conditions (37 °C, 5% CO_2_). Upon reaching 80% confluence, cells were harvested and seeded into 96 well plates at a density of 8 × 10^3^ cells per well. After 24 h of attachment, compounds **1**–**15** were added at a final concentration of 20 µM (0.1% DMSO, *v*/*v*) and incubated for an additional 24 h. Cell viability was then quantified using the cell counting kit-8 (CCK-8) assay according to the manufacturer’s instructions. After 2 h of CCK-8 reagent exposure, absorbance at 450 nm was recorded with a microplate reader, and viability was expressed as a percentage relative to vehicle-treated controls.

### 3.6. Hepatoprotective Activity Assay

ALM-12 cells were cultured in Dulbecco’s Modified Eagle Medium/Nutrient Mixture F-12 (DMEM/F12) added with dexamethasone (40 ng/mL), insulin, transferrin, selenium, 10% FBS and 1% P/S solution. Cells were delivered into a cell culture incubator under 37 °C in 5% CO_2_ for 2 d. When the density of cells came to 80%, it was inoculated into a 96 well plate for 24 h. Compounds **1**–**15** (20 µM) were immediately added into the 96 well plate for another 24 h. The hepatic injury modeling agent H_2_O_2_ (225 µM) was included for 3 h. Then a CCK-8 method was measured to testify the cell viability through the ELISA under 450 nm for the evaluation of hepatoprotective ability of these compounds.

### 3.7. Statistical Analysis

Results are expressed as mean ± SD. Inter-group differences were assessed by one-way ANOVA in GraphPad Prism 10 (The tenth version), with *p* < 0.05 taken as the threshold for statistical significance.

## 4. Conclusions

In conclusion, a comprehensive usage of isolation and purification methods contributed to the finding of three new compounds and twelve known ones from the aqueous extract of *Siraitia grosvenorii*. The structures of these compounds were elucidated using various spectroscopic techniques. The in vitro hepatoprotective activities of all the compounds were evaluated, and the results demonstrated that several of these compounds exhibited significant hepatoprotective effects. Our findings not only promoted the material-based research of *S. grosvenorii* but also highlighted the potential preventive value of these compounds derived from *S. grosvenorii* in liver diseases. Nevertheless, the mechanisms of the hepatoprotective properties of mogrosides are still in need of deeper exploration, which constitutes the reason why further investigations are supposed to put emphasis on in vivo experiments of these glycosides purified from *S. grosvenorii.*

Structurally, Luohanguonoside A–C obtained cucurbitane-type triterpenoid aglycone skeletons, similar to those of mogrosides. The only difference is in the glycosidic linkage, which explains the structural diversity of mogrosides from *S. grosvenorii*. The experimental results indicated that compounds **1**, **4**, **5**, **6**, **7**, **10**, **11**, and **14** exhibited visible hepatoprotective effects and they all had more than three sugar moieties. Meanwhile, compounds **1** and **5** possessed more than five sugar residues and exhibited the most significant efficiency in liver protection. Additionally, most other compounds without hepatoprotective activity contained only two sugar moieties, suggesting that the intensity of hepatoprotective effects is likely related to the amount of sugar residues in the isolates. In addition, among the eight bioactive compounds, five had 11-oxo-mogrol aglycones, indicating that the oxidation at C-11 of these constituents may influence their hepatoprotective ability. Thus, deeper research could also focus on the structure–activity relationship between the structures of mogrosides and their hepatoprotective properties.

*S. grosvenorii* offers a valuable reservoir of bioactive metabolites. Our work expands its chemical profile and highlights the hepatoprotection afforded by its triterpenoid glycosides, suggesting their promise to manage liver injury.

## Figures and Tables

**Table 1:** ^1^H NMR (600 MHz) and ^13^C NMR (150 MHz) data of the aglycones of compounds **1**–**3** in C_5_D_5_N (*δ* in ppm, *J* in Hz).

Position	1			2			3	
	δ_H_	δ_C_		δ_H_	δ_C_		δ_H_	δ_C_
1	2.02, m 3.00, m	26.9		1.83, m 2.02, m	20.5		1.66, m 2.11, m	21.6
2	2.23, m 2.52, m	29.5		1.27, m 1.84, m	30.2		1.85, m 1.93, m	30.2
3	3.69, br s	87.6		3.70, br s	75.6		3.73, br s	75.9
4	-	42.4		-	42.0		-	42.2
5	-	144.4		-	141.3		-	141.7
6	5.49, m	118.3		5.77, m	120.1		5.68, m	119.3
7	1.69, m 2.30, m	24.6		1.93, m 2.03, m	24.1		1.82, m 2.31, m	24.5
8	1.66, m	43.6		3.21, m	34.4		1.84, m	44.4
9	-	40.2		-	54.2		-	49.4
10	2.85, d (12.2)	36.7		2.67, d (14.7)	35.9		2.55, m	36.3
11	1.46, m 2.12, m	28.6		-	213.0		-	214.4
12	1.08, m 1.15, m	34.6		2.68, m 3.08, m	49.2		2.55, m 3.01, m	49.1
13	-	49.7		-	49.6		-	49.4
14	-	47.5		-	48.9		-	50.0
15	2.16, m 2.21, m	41.1		1.36, m 1.36, m	35.1		1.17, m 1.31, m	34.9
16	1.46, m 2.12, m	28.4		1.57, m 2.24, m	29.3		1.84, m 2.05, m	28.6
17	1.79, m	51.2		1.92, m	50.4		1.83, m	50.1
18	0.95, s	17.1		1.12, s	16.2		0.74, s	17.3
19	1.52, s	26.3		3.17, m 4.96, m	60.4		1.27, s	20.5
20	1.53, m	36.6		1.46, m	36.3		1.44, m	36.9
21	1.12, d (6.4)	19.1		1.03, d (6.4)	18.8		1.01, d (6.5)	18.7
22	1.79, m 1.87, m	33.3		1.96, m 1.77, m	33.2		1.83, m 1.83, m	33.9
23	1.58, m 1.89, m	29.8		1.50, m 1.90, m	28.5		2.05, m 2.22, m	28.7
24	3.77, m	92.3		3.77, d (9.5)	92.2		3.93, d (8.3)	88.4
25	-	72.8		-	72.8		-	72.7
26	1.43, s	24.6		1.47, s	24.6		1.48, s	26.2
27	1.34, s	27.0		1.35, s	27.1		1.51, s	26.6
28	1.17, s	27.9		1.14, s	27.8		1.15, s	28.3
29	1.52, s	26.3		1.43, s	26.6		1.44, s	27.5
30	0.94, s	19.4		1.16, s	18.9		1.03, s	19.1

**Table 2:** ^1^H NMR (600 MHz) and ^13^C NMR (150 MHz) data of the sugar residues of compounds **1**–**3** in C_5_D_5_N (*δ* in ppm, *J* in Hz.).

Position	1			2			3	
	δ_H_	δ_C_		δ_H_	δ_C_		δ_H_	δ_C_
GlcI								
G_I_-1	4.80, d (7.5)	107.0		4.93, d (7.5)	103.7		5.08, overlapped	102.2
G_I_-2	3.92, m	75.6		4.23, m	82.2		4.17, m	84.1
G_I_-3	4.15, m	78.6		4.24, m	78.6		4.34, m	78.6
G_I_-4	4.04, m	71.7		3.95, m	71.6		4.20, m	71.7
G_I_-5	4.07, m	77.4		4.12, m	76.4		3.98, m	78.9
G_I_-6	4.34, m 4.80, m	70.3		3.98, m 4.93, m	70.2		4.36, m 4.57, m	62.8
GlcII								
G_II_-1	5.19, d (7.8)	105.4		5.54, d (7.8)	105.5		5.40, d (7.7)	106.6
G_II_-2	4.05, m	75.3		4.13, m	75.6		4.15, m	76.6
G_II_-3	4.27, m	78.2		4.25, m	78.4		4.25, m	78.7
G_II_-4	4.26, m	71.5		4.15, m	72.5		4.19, m	72.5
G_II_-5	3.97, m	78.5		3.97, m	78.2		3.98, m	78.8
G_II_-6	4.40, m 4.53, m	62.6		4.35, m 4.54, m	63.5		4.40, m 4.57, m	63.6
GlcIII								
G_III_-1	4.93, d (7.6)	103.7		4.88, d (7.7)	104.9			
G_III_-2	4.15, m	82.6		4.08, m	75.5			
G_III_-3	4.23, m	76.3		4.27, m	78.1			
G_III_-4	3.94, m	71.5		4.27, m	71.5			
G_III_-5	4.08, m	76.4		3.93, m	78.8			
G_III_-6	3.98, m 4.92, m	70.2		4.37, m 4.52, m	62.6			
GlcIV								
G_IV_-1	5.44, d (7.9)	105.4						
G_IV_-2	4.11, m	75.4						
G_IV_-3	4.24, m	76.7						
G_IV_-4	4.23, m	82.4						
G_IV_-5	3.94, m	78.6						
G_IV_-6	4.48, m 4.50, m	63.2						
GlcV								
G_V_-1	4.88, d (7.6)	104.9						
G_V_-2	4.07, m	75.6						
G_V_-3	4.27, m	78.3						
G_V_-4	4.26, m	71.7						
G_V_-5	3.93, m	78.5						
G_V_-6	4.31, m 4.55, m	62.5						
GlcVI								
G_VI_-1	5.13, d (7.9)	105.0						
G_VI_-2	4.09, m	75.4						
G_VI_-3	4.20, m	78.1						
G_VI_-4	4.23, m	71.5						
G_VI_-5	4.01, m	78.1						
G_VI_-6	4.31, m 4.55, m	62.5						

## Data Availability

The original contributions presented in this study are included in the article/Supplementary Material. Further inquiries can be directed to the corresponding author(s).

## Supplementary Materials

The following supporting information can be downloaded at: https://www.mdpi.com/article/10.3390/molecules30193983/s1. Figures S1–S8: HRESIMS plot, 1D and 2D NMR spectra of **1**; Figures S9–S16: HRESIMS plot, 1D and 2D NMR spectra of **2**; Figures S17–S24: HRESIMS plot, 1D and 2D NMR spectra of **3**; Figures S25–S27: IR spectra of **1**–**3**. Figures S28: UV spectra of **1**–**6**. Figure S29: sugars acid hydrolysis experiment of **1**–**6.** Figure S30: Chemical structures of compounds **1**–**15** isolated from *S. grosvenorii* fruit. Figure S31: Structures and key 2D-NMR correlations of compounds **1**–**3**. Figure S32: Cytotoxicity of compounds **1**–**15.** Figure S33: Hepatoprotective activities of compounds **1**–**15**. Table S1: ^1^H NMR (600 MHz) and ^13^C NMR (150 MHz) data of the aglycones of compounds **1**–**3** in C_5_D_5_N; Table S2: ^1^H NMR (600 MHz) and ^13^C NMR (150 MHz) data of the sugar residues of compounds **1**–**3** in C_5_D_5_N. Appendix A contains the HRESIMS, 1D and 2D plots of compounds 4–15.

## Abbreviations

The following abbreviations are used in this manuscript:

^1^H NMR	^1^H Nuclear Magnetic Resonance
^13^C NMR	^13^C Nuclear Magnetic Resonance
^1^H-^1^H COSY	^1^H-^1^H Correlation Spectroscopy
HSQC	Heteronuclear Single Quantum Coherence
HMBC	Heteronuclear Multiple Bond Correlation
NOESY	Nuclear Overhauser Effect Spectroscopy
TOCSY	Total Correlation Spectroscopy
HRESIMS	High Resolution Electrospray Ionization Mass Spectrometry
UV	Ultraviolet
IR	Infrared
HPLC	High-Performance Liquid Chromatography
*t*_R_	retention time

## References

[1] Li C., Lin L.-M., Sui F., Wang Z.-M., Huo H.-R., Dai L., Jiang T.-L. (2014). Chemistry and pharmacology of *Siraitia grosvenorii*: A review. Chin. J. Nat. Med..

[2] Jin J.-S., Lee J.-H. (2012). Phytochemical and pharmacological aspects of *Siraitia grosvenorii*, luo han kuo. Orient. Pharm. Exp. Med..

[3] Huang H., Peng Z., Zhan S., Li W., Liu D., Huang S., Zhu Y., Wang W. (2024). A comprehensive review of *Siraitia grosvenorii* (Swingle) C. Jeffrey: Chemical composition, pharmacology, toxicology, status of resources development, and applications. Front. Pharmacol..

[4] Muñoz-Labrador A., Hernandez-Hernandez O., Moreno F.J. (2023). A review of the state of sweeteners science: The natural versus artificial non-caloric sweeteners debate. *Stevia rebaudiana* and *Siraitia grosvenorii* into the spotlight. Crit. Rev. Biotechnol..

[5] Di R., Huang M.-T., Ho C.-T. (2011). Anti-inflammatory Activities of Mogrosides from Momordica *grosvenori* in Murine Macrophages and a Murine Ear Edema Model. J. Agric. Food Chem..

[6] Guo Q., Shi M., Sarengaowa, Xiao Z., Xiao Y., Feng K. (2024). Recent Advances in the Distribution, Chemical Composition, Health Benefits. and Application of the Fruit of *Siraitia grosvenorii*. Foods.

[7] Chen N., Cao W., Yuan Y., Wang Y., Zhang X., Chen Y., Yiasmin M.N., Tristanto N.A., Hua X. (2024). Recent advancements in mogrosides: A review on biological activities, synthetic biology, and applications in the food industry. Food Chem..

[8] Liu H., Wang C., Qi X., Zou J., Sun Z. (2018). Antiglycation and antioxidant activities of mogroside extract from *Siraitia grosvenorii* (Swingle) fruits. J. Food Sci. Technol..

[9] Shen J., Shen D., Tang Q., Li Z., Jin X., Li C. (2022). Mogroside V exerts anti-inflammatory effects on fine particulate matter-induced inflammation in porcine alveolar macrophages. Toxicol. Vitr..

[10] Zhou Y., Hu Z., Ye F., Guo T., Luo Y., Zhou W., Qin D., Tang Y., Cao F., Luo F. (2021). Mogroside V exerts anti-inflammatory effect via MAPK-NF-κB/AP-1 and AMPK-PI3K/Akt/mTOR pathways in ulcerative colitis. J. Funct. Foods.

[11] Zhang X., Song Y., Ding Y., Wang W., Liao L., Zhong J., Sun P., Lei F., Zhang Y., Xie W. (2018). Effects of Mogrosides on High-Fat-Diet-Induced Obesity and Nonalcoholic Fatty Liver Disease in Mice. Molecules.

[12] Cao F., Zhang Y., Li W., Shimizu K., Xie H., Zhang C. (2018). Mogroside IVE attenuates experimental liver fibrosis in mice and inhibits HSC activation through downregulating TLR4-mediated pathways. Int. Immunopharmacol..

[13] Zhang Y., Peng Y., Zhao L., Zhou G., Li X. (2021). Regulating the gut microbiota and SCFAs in the faeces of T2DM rats should be one of antidiabetic mechanisms of mogrosides in the fruits of *Siraitia grosvenorii*. J. Ethnopharmacol..

[14] Zou C., Zhang Q., Zhang S. (2018). Mogroside IIIE attenuates gestational diabetes mellitus through activating of AMPK signaling pathway in mice. J. Pharmacol. Sci..

[15] Zhang Y., Zhou G., Peng Y., Wang M., Li X. (2020). Anti-hyperglycemic and anti-hyperlipidemic effects of a special fraction of Luohanguo extract on obese T2DM rats. J. Ethnopharmacol..

[16] Takasaki M., Konoshima T., Murata Y., Sugiura M., Nishino H., Tokuda H., Matsumoto K., Kasai R., Yamasaki K. (2003). Anticarcinogenic activity of natural sweeteners, cucurbitane glycosides, from *Momordica grosvenori*. Cancer Lett..

[17] Luo J., Lu D., Zhang R., Long B., Chen L., Wang W., Tian X. (2025). What exactly happens to rats that drink different types of sweetness water over a long time:A comparison with sucrose, artificial sweeteners and natural sweeteners. J. Funct. Foods.

[18] Luo Q.-J., Zhou W.-C., Liu X.-Y., Li Y.-J., Xie Q.-L., Wang B., Liu C., Wang W.-M., Wang W., Zhou X.-D. (2023). Chemical Constituents and α-Glucosidase Inhibitory, Antioxidant and Hepatoprotective Activities of *Ampelopsis grossedentata*. Molecules.

[19] Jia Y.Z., Yang Y.P., Cheng L., Cao S.W., Xie Q.L., Wang M.Y., Li B., Jian Y.Q., Liu B., Peng C.Y. (2021). Heilaohuguosus A-S from the fruits of *Kadsura coccinea* and their hepatoprotective activity. Phytochemistry.

[20] Li F., Yang F., Liu X., Wang L., Chen B., Li L., Wang M. (2017). Cucurbitane glycosides from the fruit of *Siraitia grosvenori* and their effects on glucose uptake in human HepG2 cells in vitro. Food Chem..

[21] Chaturvedula V.S.P., Prakash I. (2011). Cucurbitane Glycosides from *Siraitia grosvenorii*. J. Carbohydr. Chem..

[22] Akihisa T., Hayakawa Y., Tokuda H., Banno N., Shimizu N., Suzuki T., Kimura Y. (2007). Cucurbitane Glycosides from the Fruits of *Siraitia grosvenorii* and Their Inhibitory Effects on Epstein−Barr Virus Activation. J. Nat. Prod..

[23] Niu B., Ke C.-Q., Li B.-H., Li Y., Yi Y., Luo Y., Shuai L., Yao S., Lin L.-G., Li J. (2017). Cucurbitane Glucosides from the Crude Extract of *Siraitia grosvenorii* with Moderate Effects on PGC-1α Promoter Activity. J. Nat. Prod..

[24] Chu D., Yaseen A., Wang L., Chen B., Wang M., Hu W., Li F. (2019). Two New Cucurbitane Glycosides from the Fruits of *Siraitia grosvenori*. Chem. Pharm. Bull..

[25] Suzuki Y.A., Murata Y., Inui H., Sugiura M., Nakano Y. (2005). Triterpene Glycosides of *Siraitia grosvenori* Inhibit Rat Intestinal Maltase and Suppress the Rise in Blood Glucose Level after a Single Oral Administration of Maltose in Rats. J. Agric. Food Chem..

[26] Matsumoto K., Kasai R., Ohtani K., Tanaka O. (1990). Minor cucurbitane-glycosides from fruits of *Siraitia grosvenori* (Cucurbitaceae). Chem. Pharm. Bull..

[27] Prakash I., Chaturvedula V. (2014). Additional New Minor Cucurbitane Glycosides from *Siraitia grosvenorii*. Molecules.

[28] Oobayashi K., Yoshikawa K., Arihara S. (1992). Structural revision of bryonoside and structure elucidation of minor saponins from *Bryonia dioica*. Phytochemistry.

