# Supplementary material for: Cucurbitane Glycosides from Siraitia Grosvenorii and Their Hepatoprotective Activities
Source: Molecules. 2025 Oct 4;30(19):3983. doi: 10.3390/molecules30193983 (PMC12526448; doi:10.3390/molecules30193983)

Supplementary materials

Cucurbitane Glycosides from *Siraitia Grosvenorii* and Their Hepatoprotective Activities

Jia-Nan Mao [1,2], Hua-Xue Huang [1,2,3,4,5], Qing-Ling Xie [1,2], Guang-Yu Chen [1,2], Juan-Jiang Wu [1,2,3], Ying Deng [1,2], Shuang Zhan [4,5], Zhi Peng [4,5], Xu-Dong Zhou [1,2,*] and Wei Wang [1,2,*]

[1] TCM and Ethnomedicine Innovation & Development International Laboratory, Innovative Materia Medica Research Institute, School of Pharmacy, Hunan University of Chinese Medicine, Changsha 410208, China; 19313189907@139.com (J.-N.M.); river@huachengbio.com (H.-X.H.); xieql12@126.com (Q.-L.X.); 004581@hnucm.edu.cn (G.-Y.C.); 3230004587@student.must.edu.mo (J.-J.W.); dengying028@163.com (Y.D.)

[2] Modernization Industry College for Innovative Chinese Medicine, Hunan University of Chinese Medicine, Changsha 410208, China

[3] Faculty of Chinese Medicine, Macau University of Science and Technology, Macau SAR 999078, China

[4] Research and Development Institute of Hunan Huacheng Biotech, Inc., Changsha 410205, China; support@huachengbio.com (S.Z.); pengzhi3778@163.com (Z.P.)

[5] Hunan Natural Sweetener Engineering Technology Research Center, Changsha 410205, China

* Correspondence: to: xudongzhou999@hnucm.edu.cn (X.-D.Z.); wangwei402@hotmail.com (W.W.)

Contents

Figure S1. HRESIMS spectrum of **1**

Figure S2. ^{1}H-NMR spectrum of **1** in PYRIDINE-d_5

Figure S3. ^{13}C-NMR spectrum of **1** in PYRIDINE-d_5

Figure S4. ^{1}H-^{1}H Cosy spectrum of compound **1** in PYRIDINE-d_5

Figure S5. HSQC spectrum of **1** in PYRIDINE-d_5

Figure S6. HMBC spectrum of **1** in PYRIDINE-d_5

Figure S7. NOESY spectrum of **1** in PYRIDINE-d_5

Figure S8. TOCSY spectrum of **1** in PYRIDINE-d_5

Figure S9. HRESIMS spectrum of **2**

Figure S10. ^{1}H-NMR spectrum of **2** in PYRIDINE-d_5

Figure S11. ^{13}C-NMR spectrum of **2** in PYRIDINE-d_5

Figure S12. ^{1}H-^{1}H Cosy spectrum of compound **2** in PYRIDINE-d_5

Figure S13. HSQC spectrum of **2** in PYRIDINE-d_5

Figure S14. HMBC spectrum of **2** in PYRIDINE-d_5

Figure S15. NOESY spectrum of **2** in PYRIDINE-d_5

Figure S16. TOCSY spectrum of **2** in PYRIDINE-d_5

Figure S17. HRESIMS spectrum of **3**

Figure S18. ^{1}H-NMR spectrum of **3** in PYRIDINE-d_5

Figure S19. ^{13}C-NMR spectrum of **3** in PYRIDINE-d_5

Figure S20. ^{1}H-^{1}H Cosy spectrum of compound **3** in PYRIDINE-d_5

Figure S21. HSQC spectrum of **3** in PYRIDINE-d_5

Figure S22. HMBC spectrum of **3** in PYRIDINE-d_5

Figure S23. NOESY spectrum of **3** in PYRIDINE-d_5

Figure S24. TOCSY spectrum of **3** in PYRIDINE-d_5

Figure S25. IR spectroscopy of **1**

Figure S26. IR spectroscopy of **2**

Figure S27. IR spectroscopy of **3**

Figure S28. UV spectroscopy of **1-6**

Figure S29. Glycoside Hydrolysis Experiment of **1-6**

Figure S30. Structures of compounds **1–15** isolated from *S. grosvenorii* fruit.

Figure S31. Structures and key 2D-NMR correlations of compounds **1–3**.

Figure S32. Cytotoxicity of compounds **1–15.**

Figure S33. Hepatoprotective activities of compounds **1–15**.

Table S1. ^{1}H NMR (600 MHz) and ^{13}C NMR (150 MHz) data of the aglycones of compounds **1–3** in C_5D_5N.

Table S2. ^{1}H NMR (600 MHz) and ^{13}C NMR (150 MHz) data of the sugars residues of compounds **1–3** in C_5D_5N.

Appendix A 1D-NMR, 2D-NMR and HRESIMS of compounds **4-15**

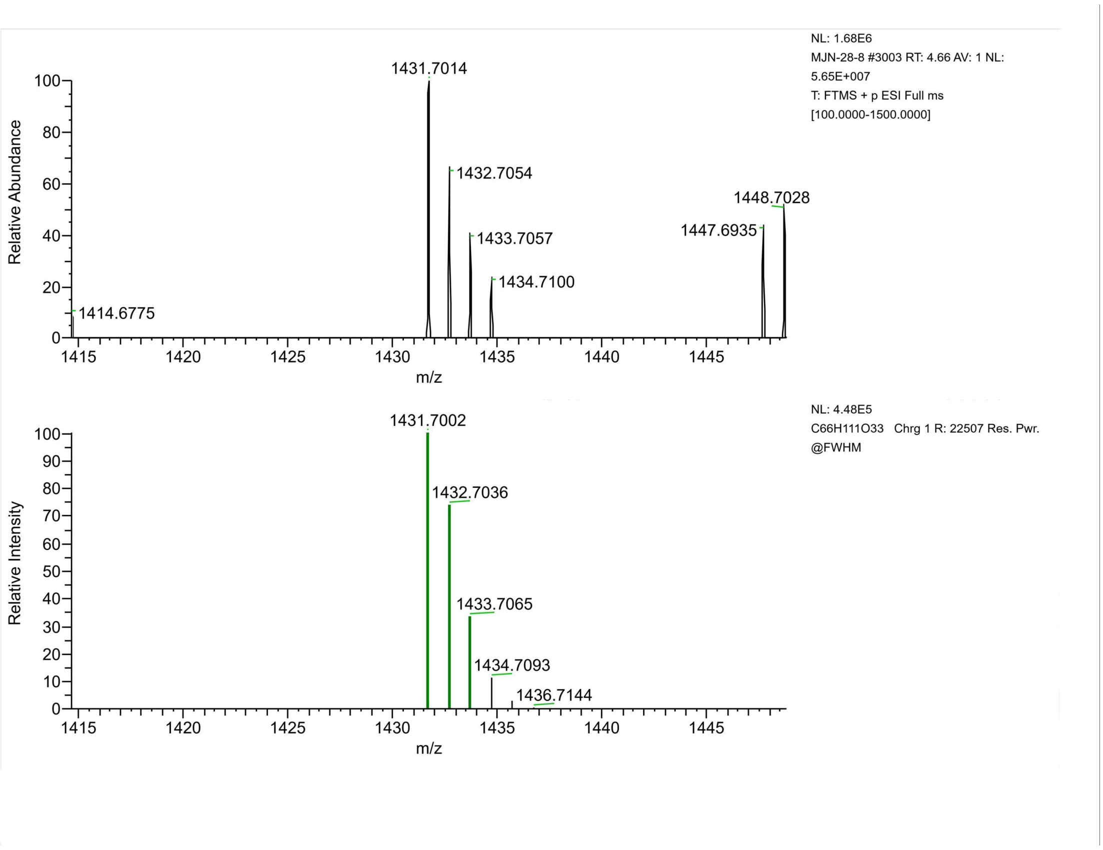

$[M+K]^+$

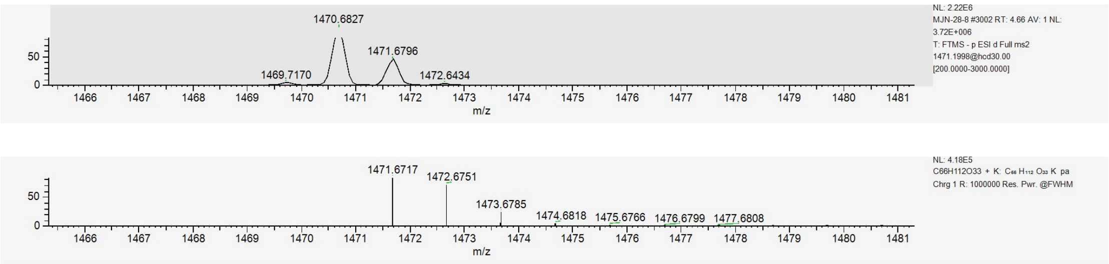

$[M-H]^-$

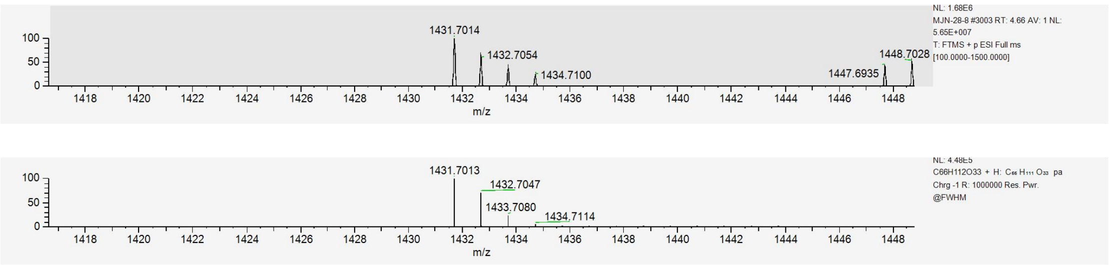

Figure S1. HRESIMS spectrum of **1** in CH_3OH

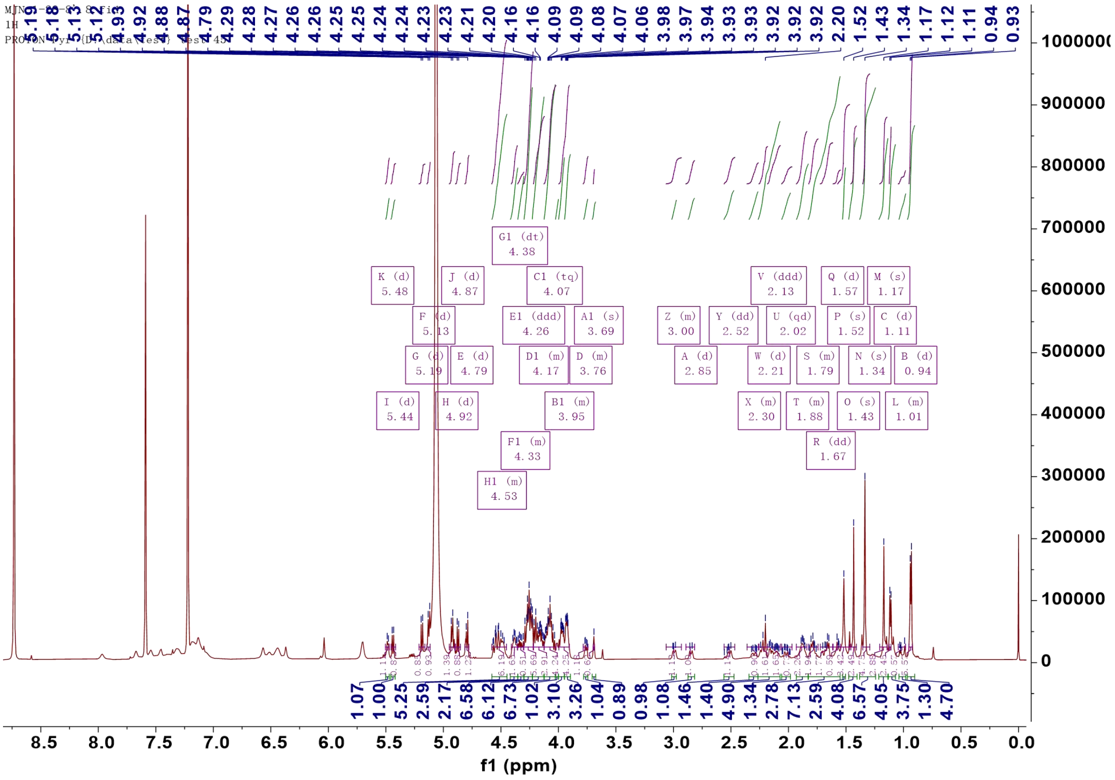

Figure S2. ^{1}H-NMR spectrum of **1** in PYRIDINE-d_5

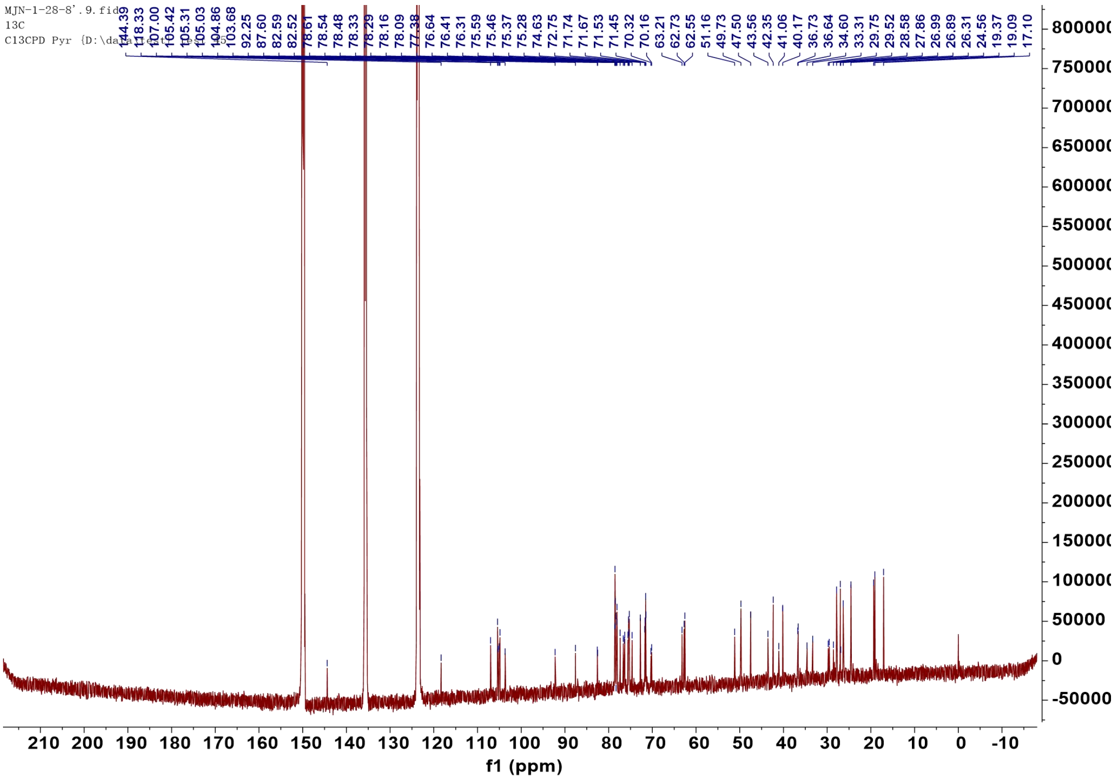

Figure S3. ^{13}C-NMR spectrum of **1** in PYRIDINE-*d*$_5$

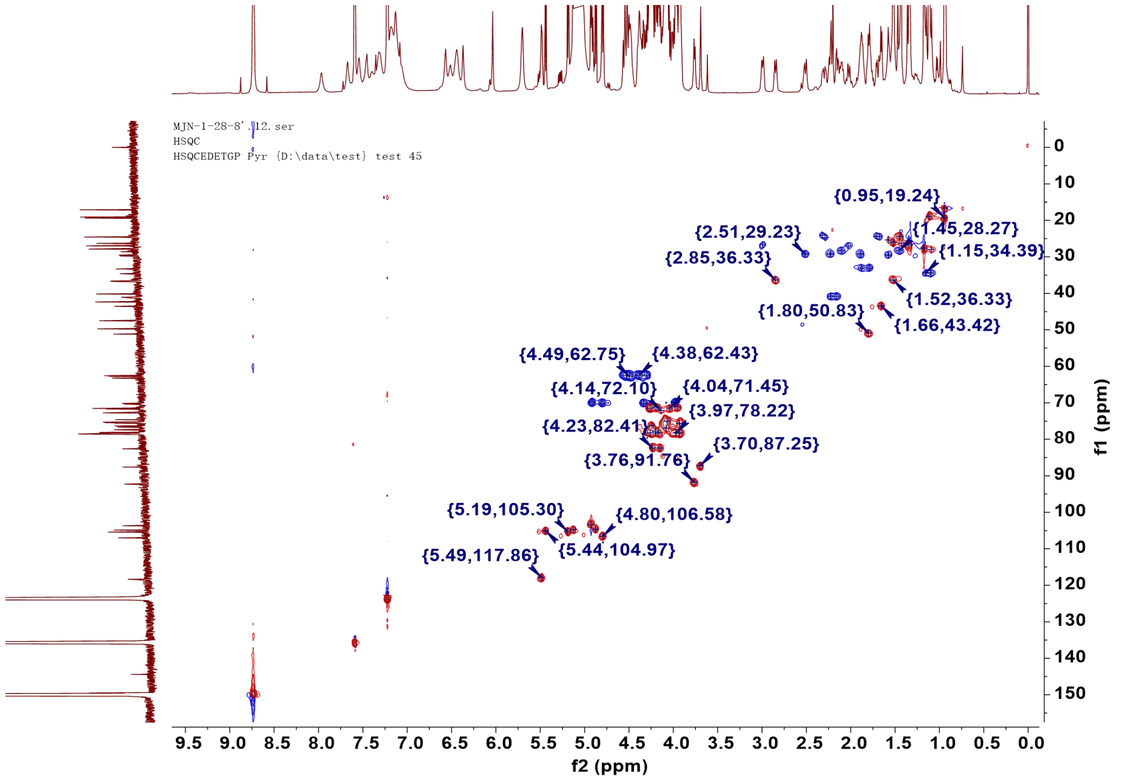

Figure S4. HSQC spectrum of **1** in PYRIDINE-*d*$_5$

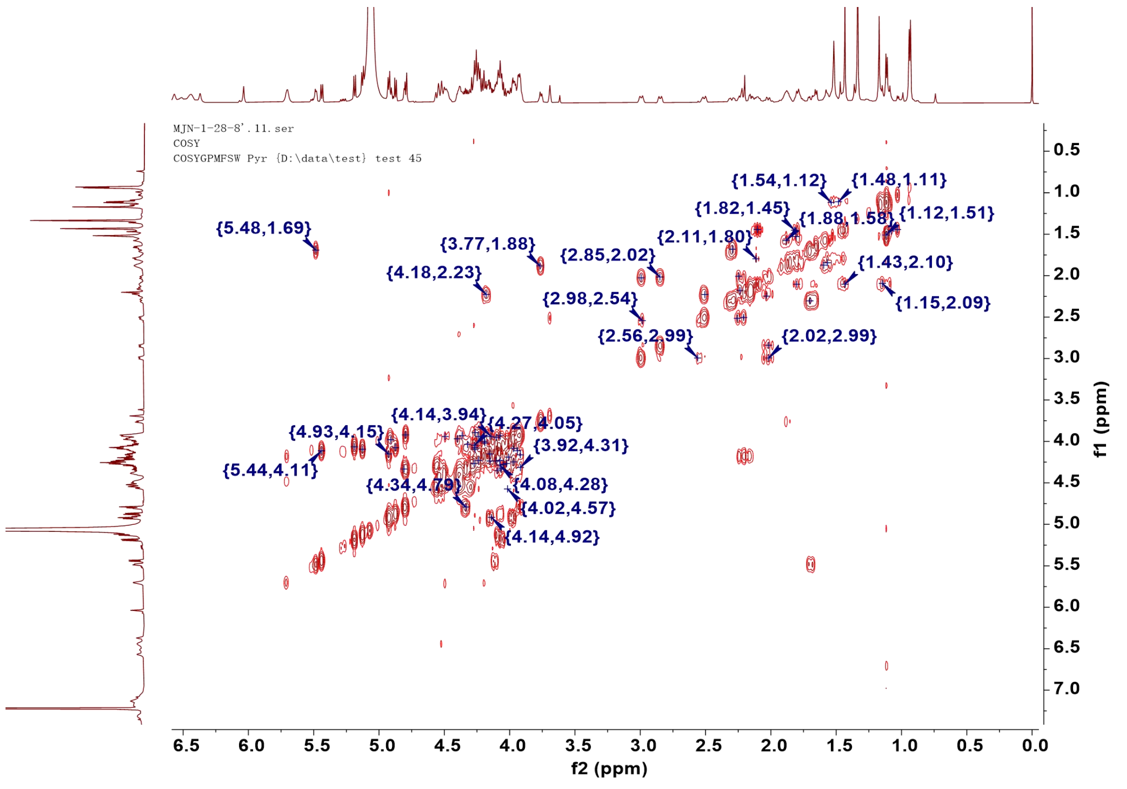

Figure S5. ^{1}H-^{1}H Cosy spectrum of **1** in PYRIDINE-d_5

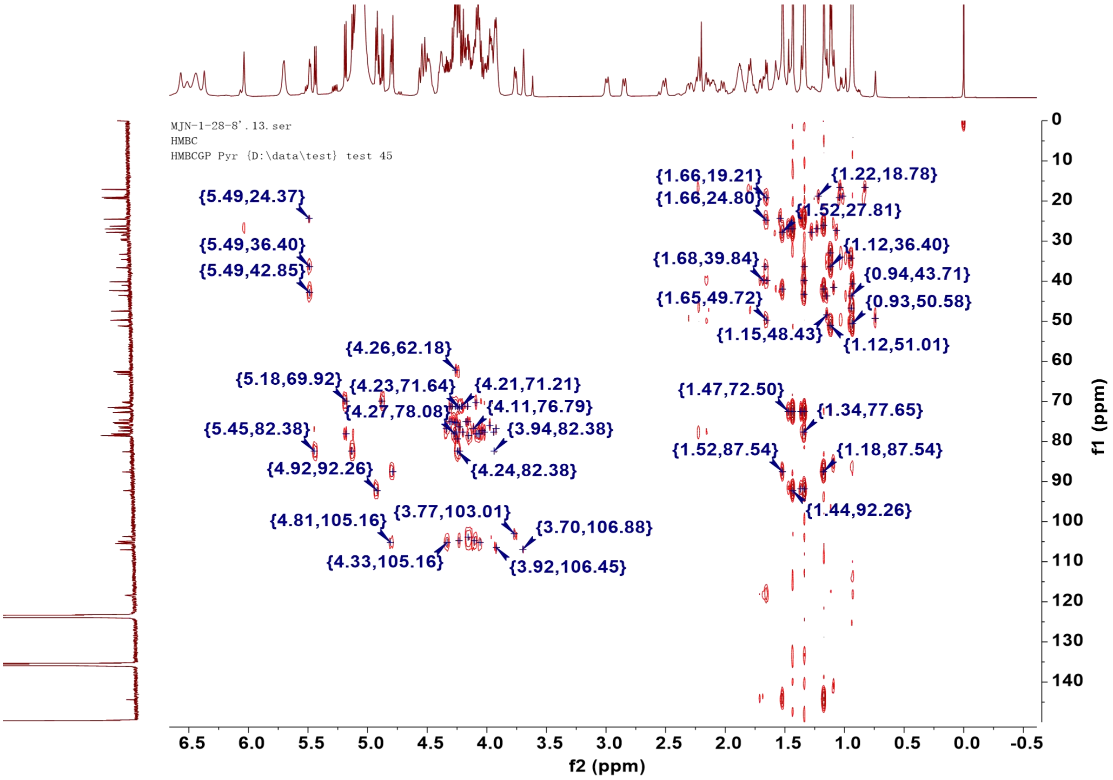

Figure S6. HMBC of compound **1** PYRIDINE-d_5

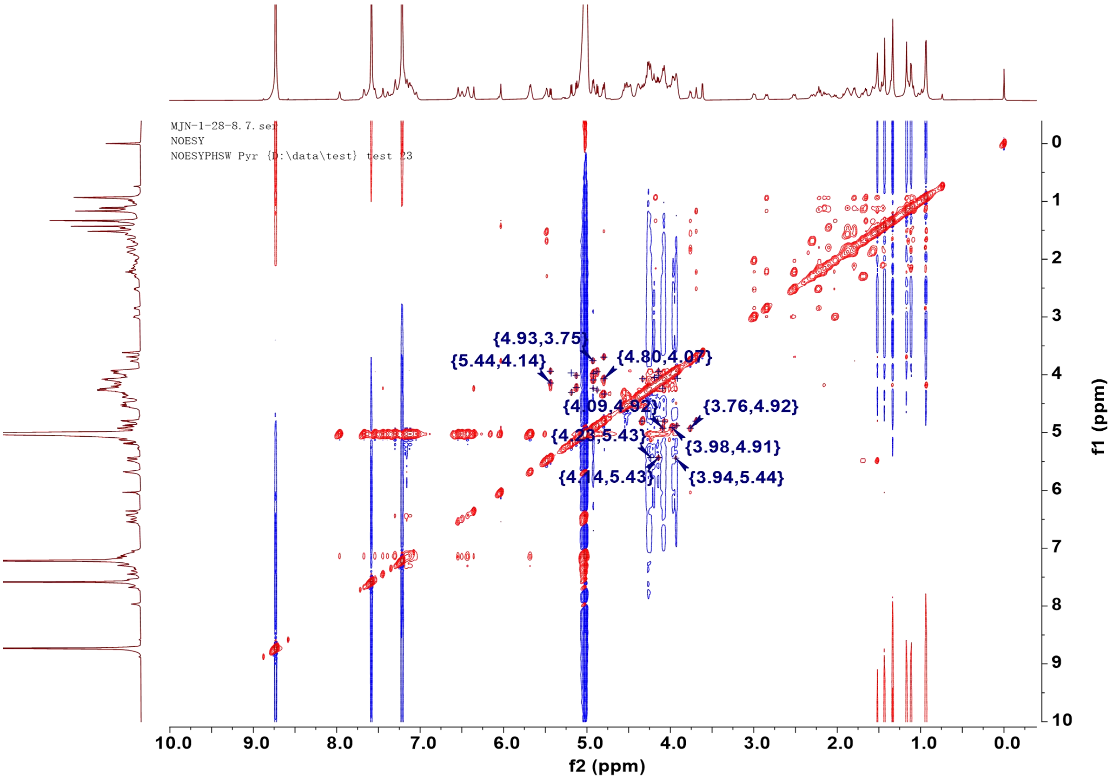

Figure S7. NOESY of compound **1** PYRIDINE-d_5

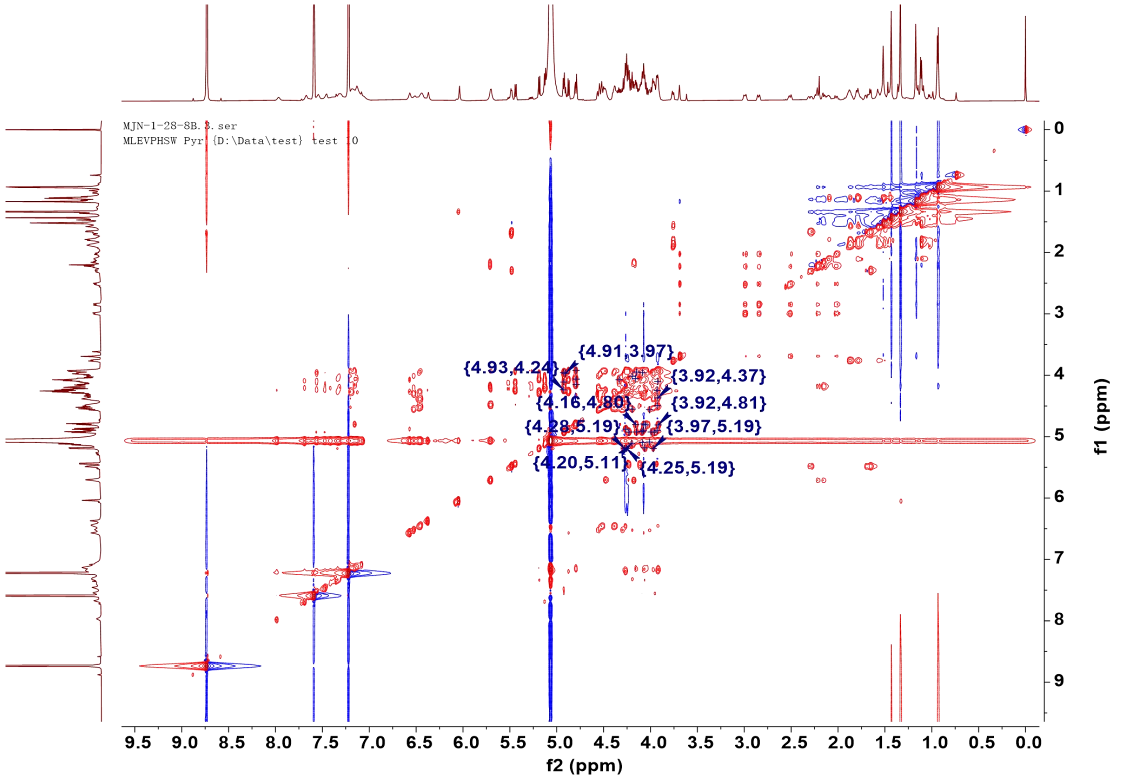

Figure S8. TOCSY of compound **1** PYRIDINE-d_5

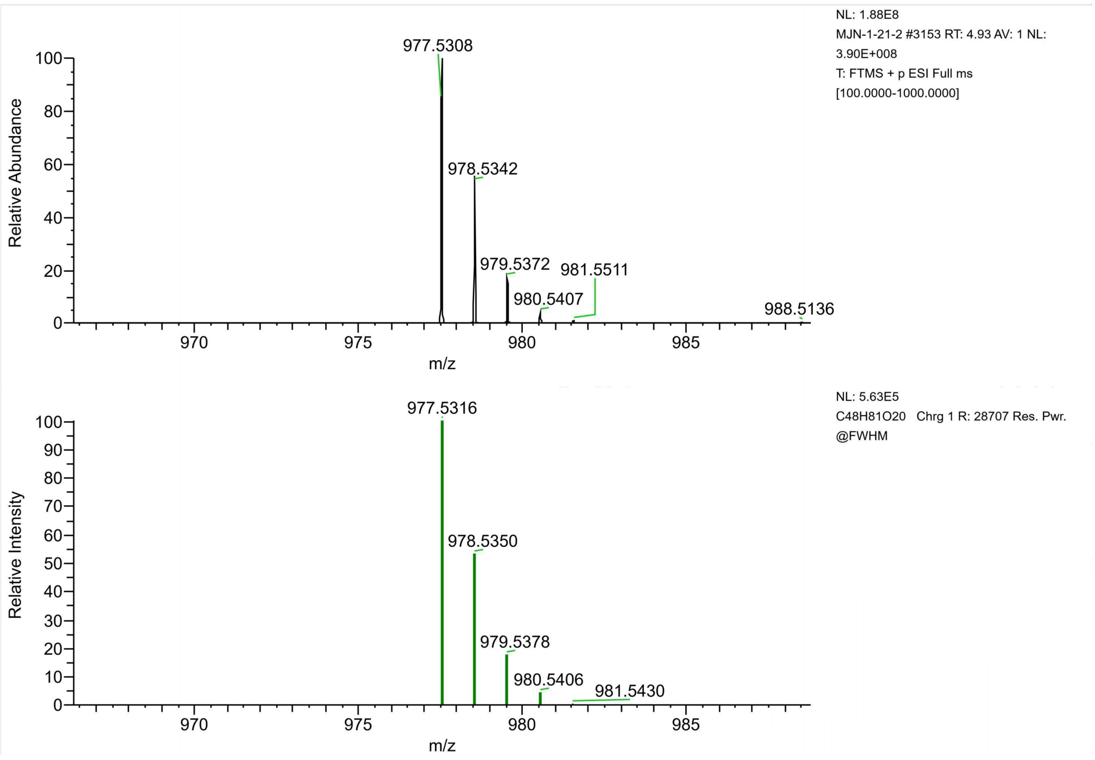

$[M+Na]^+$

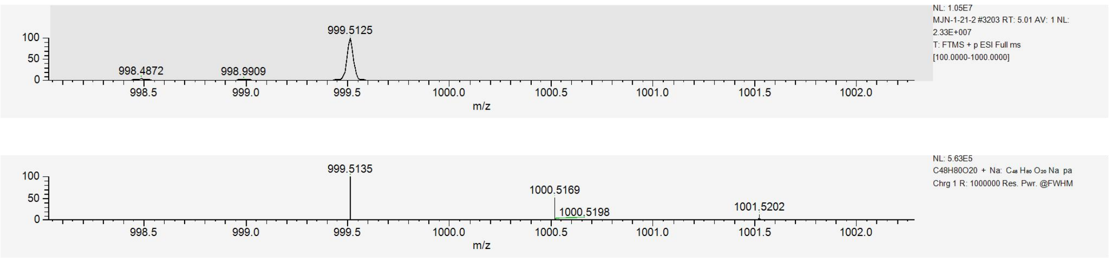

$[M+H]^+$

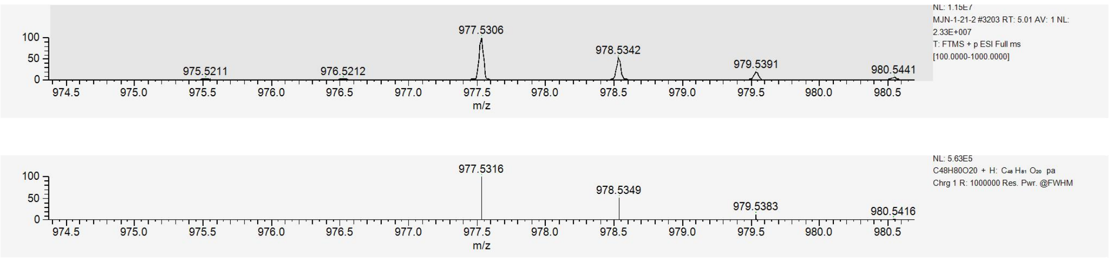

Figure S9. HRESIMS spectrum of **2** in CH_3OH

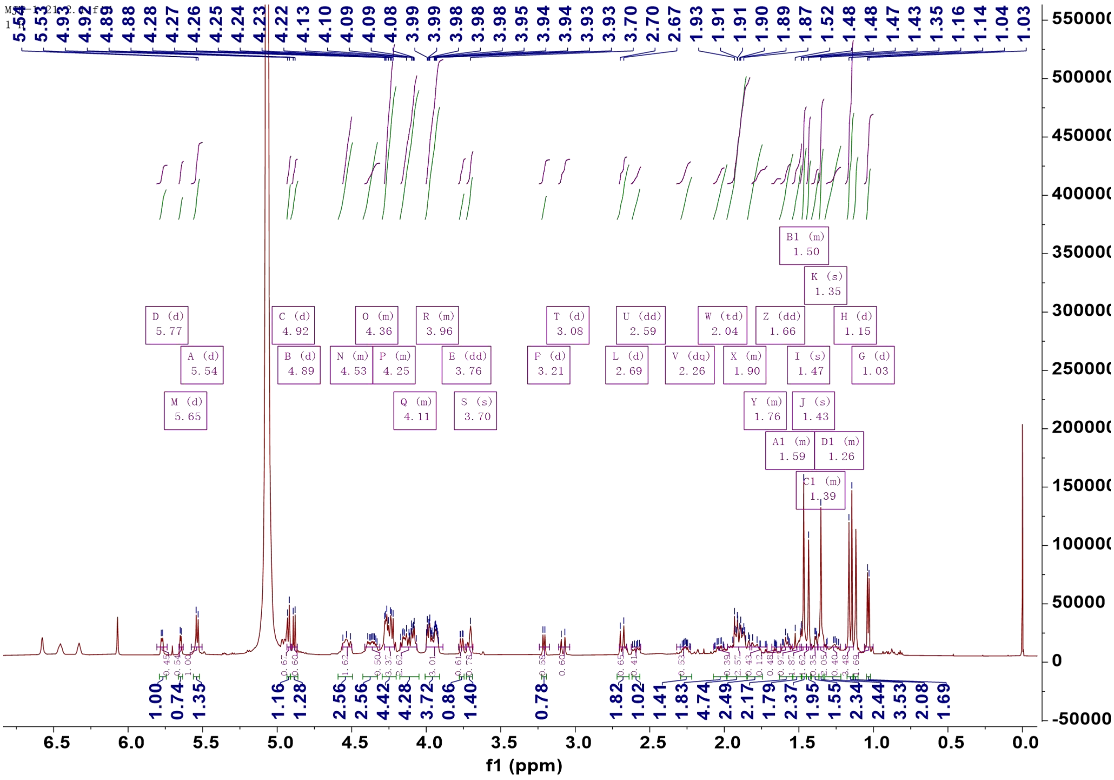

Figure S10. ^{1}H-NMR spectrum of **2** in PYRIDINE-d_5

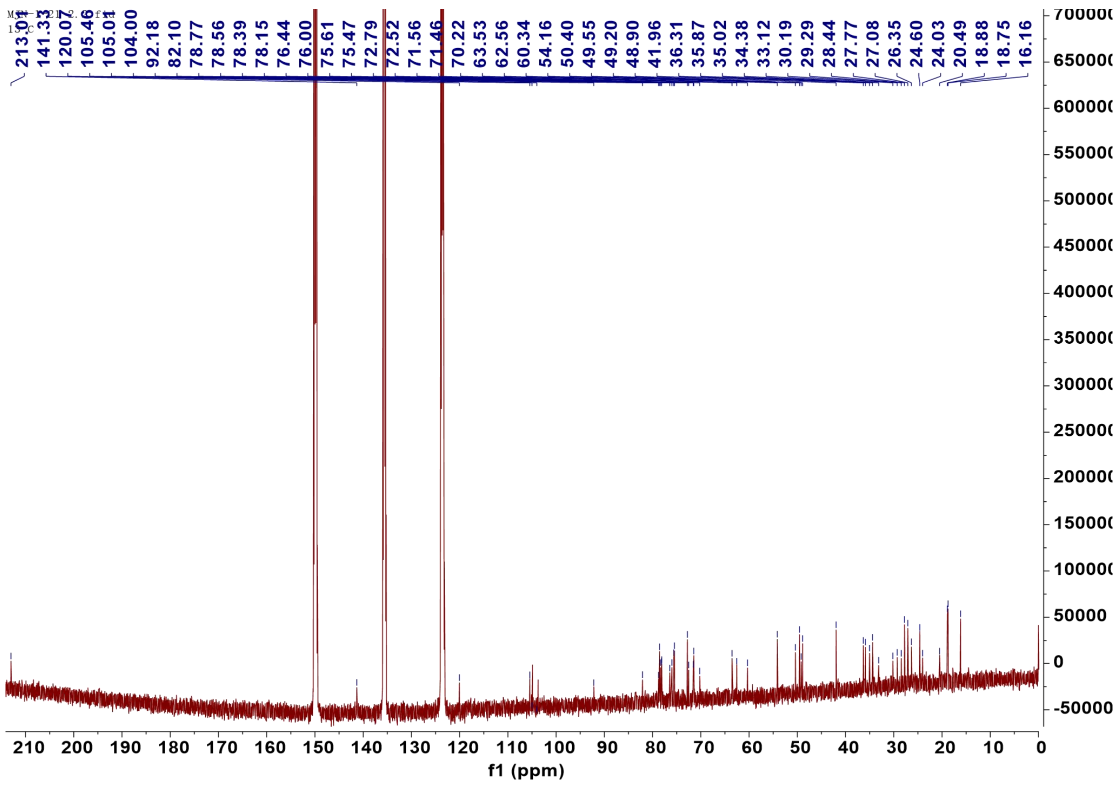

Figure S11. ^{13}C-NMR spectrum of **2** in PYRIDINE-d_5

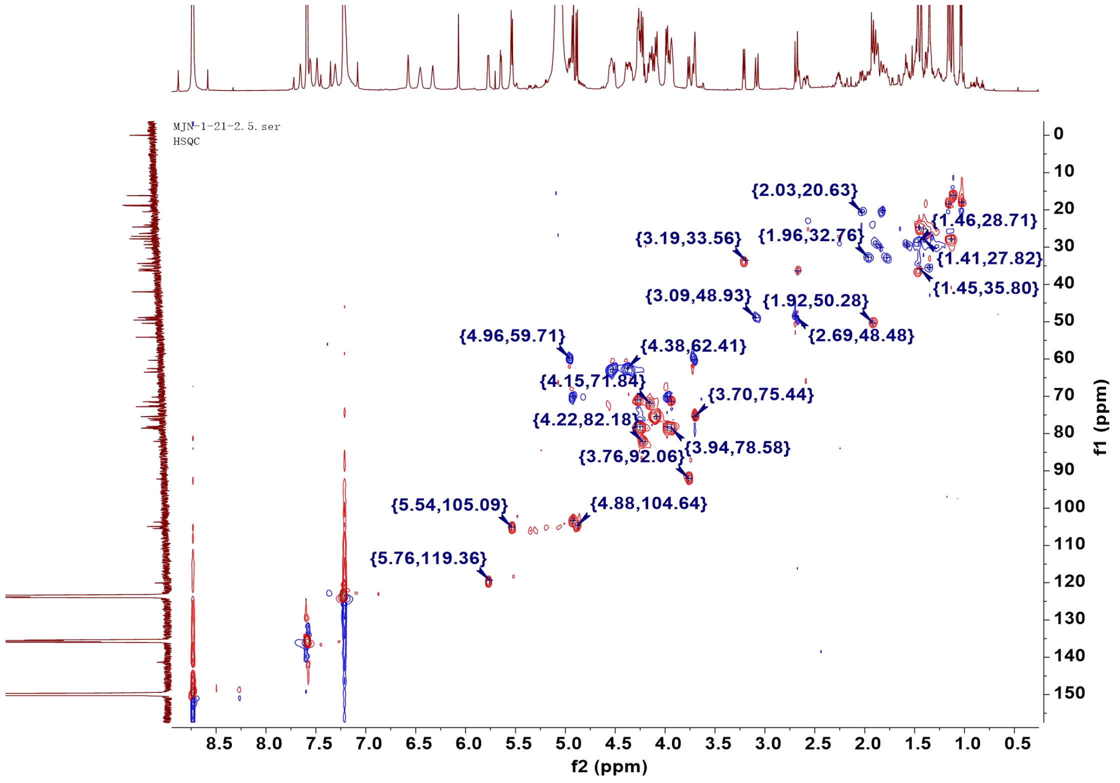

Figure S12. HSQC spectrum of **2** in PYRIDINE-d_5

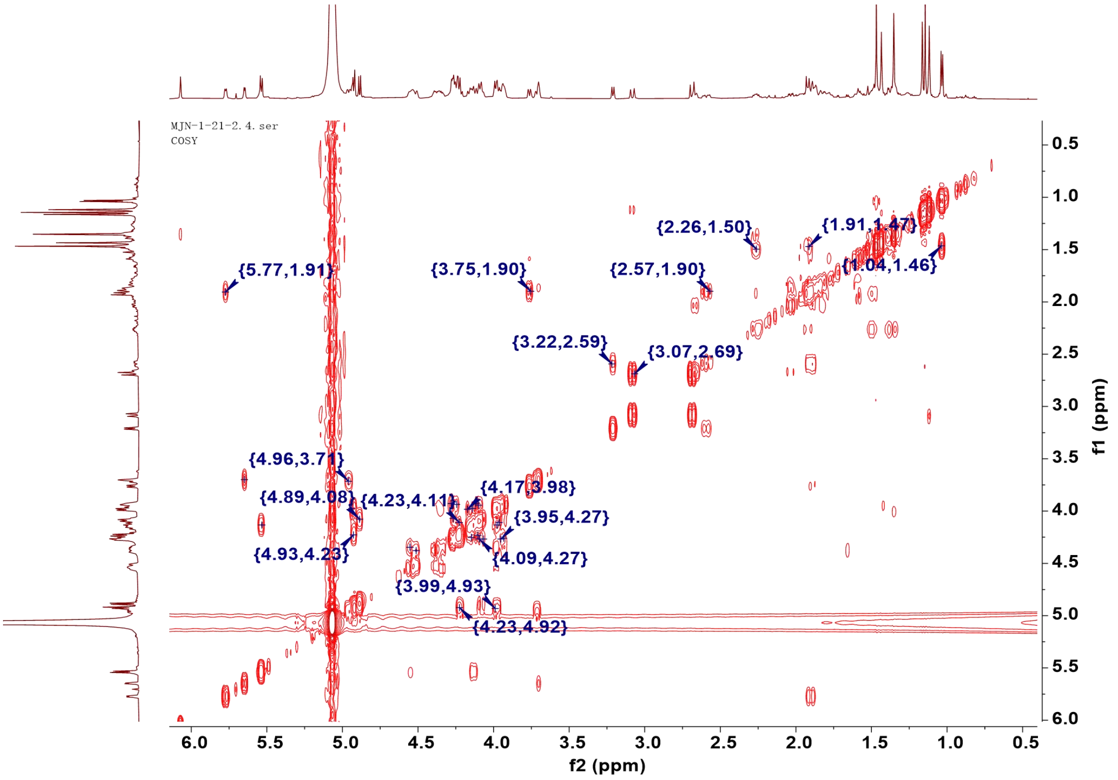

Figure S13. ^{1}H-^{1}H Cosy spectrum of **2** in PYRIDINE-d_5

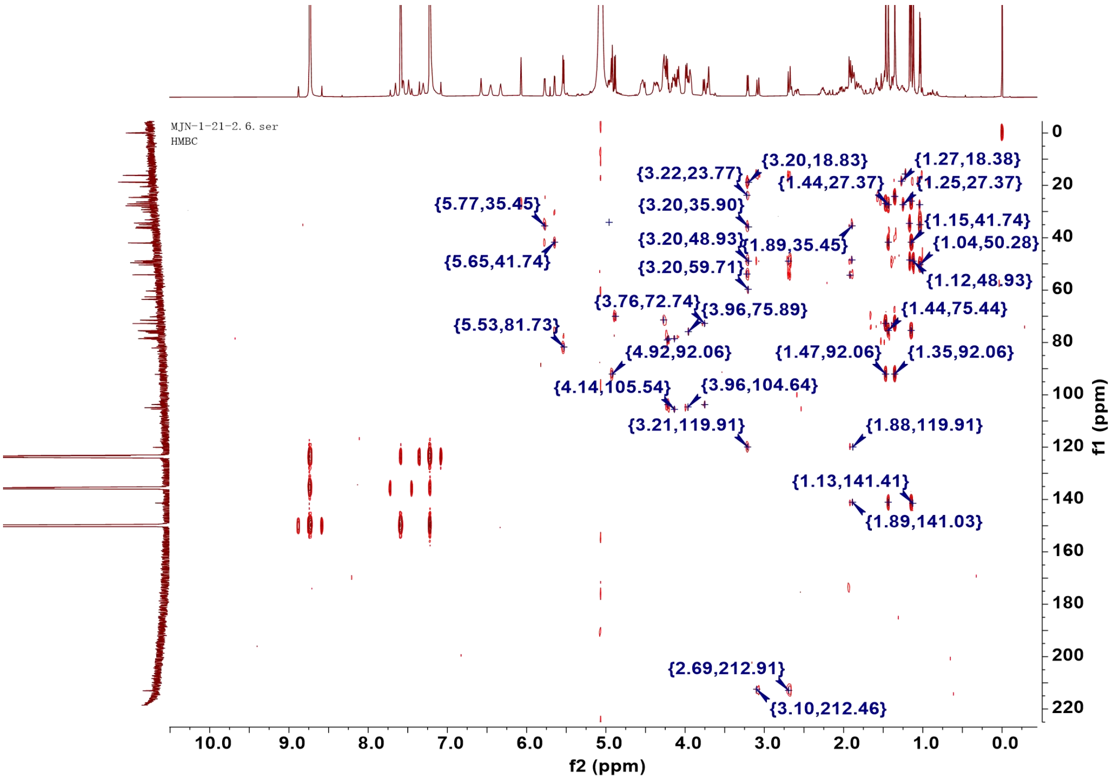

Figure S14. HMBC spectrum of **2** in PYRIDINE-d_5

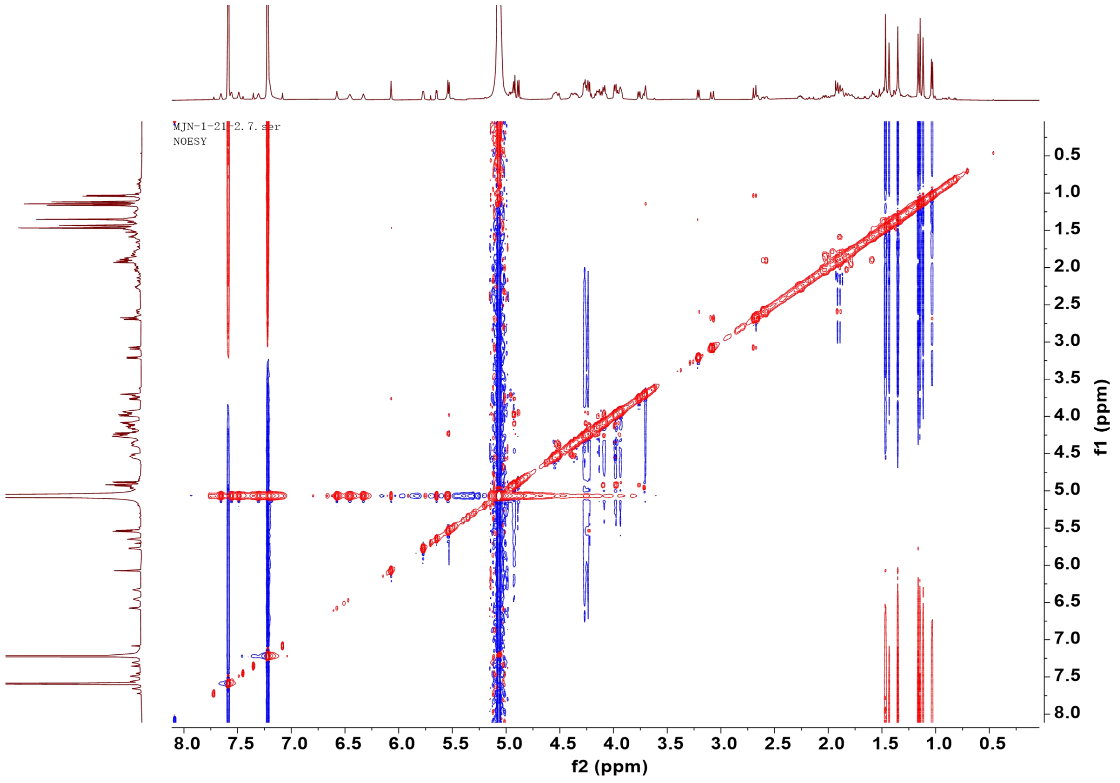

Figure S15. NOESY spectrum of **2** in PYRIDINE-d_5

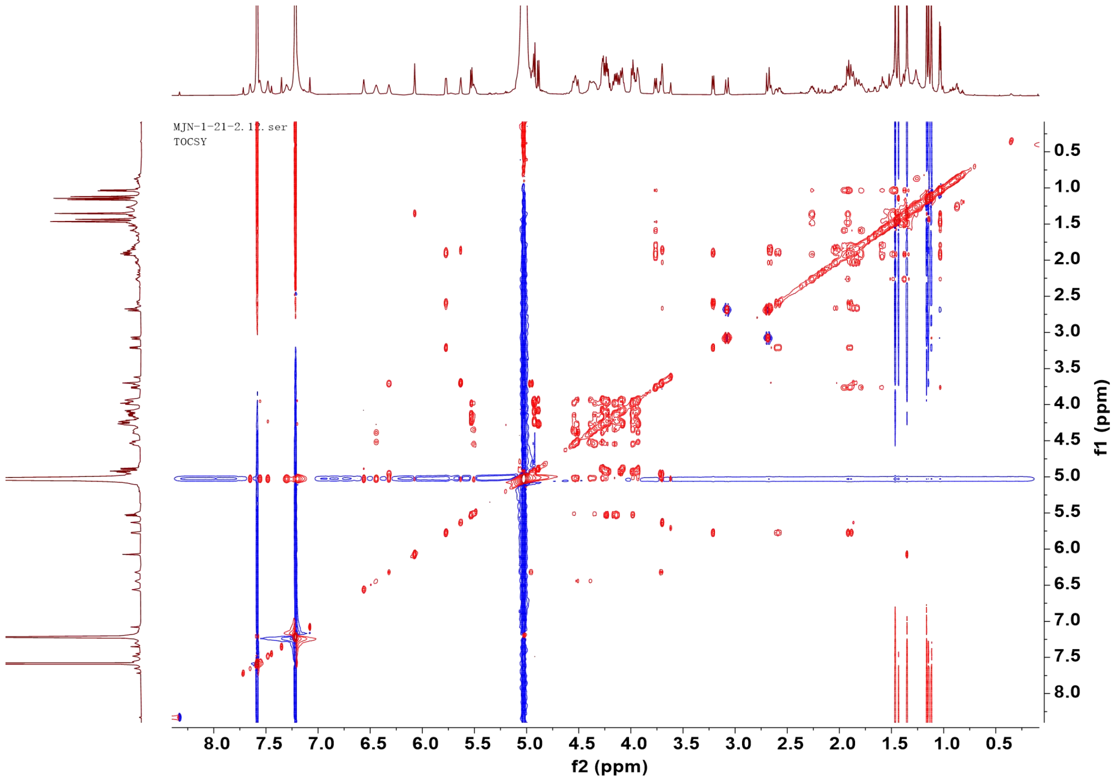

Figure S16. TOCSY spectrum of **2** in PYRIDINE-d_5

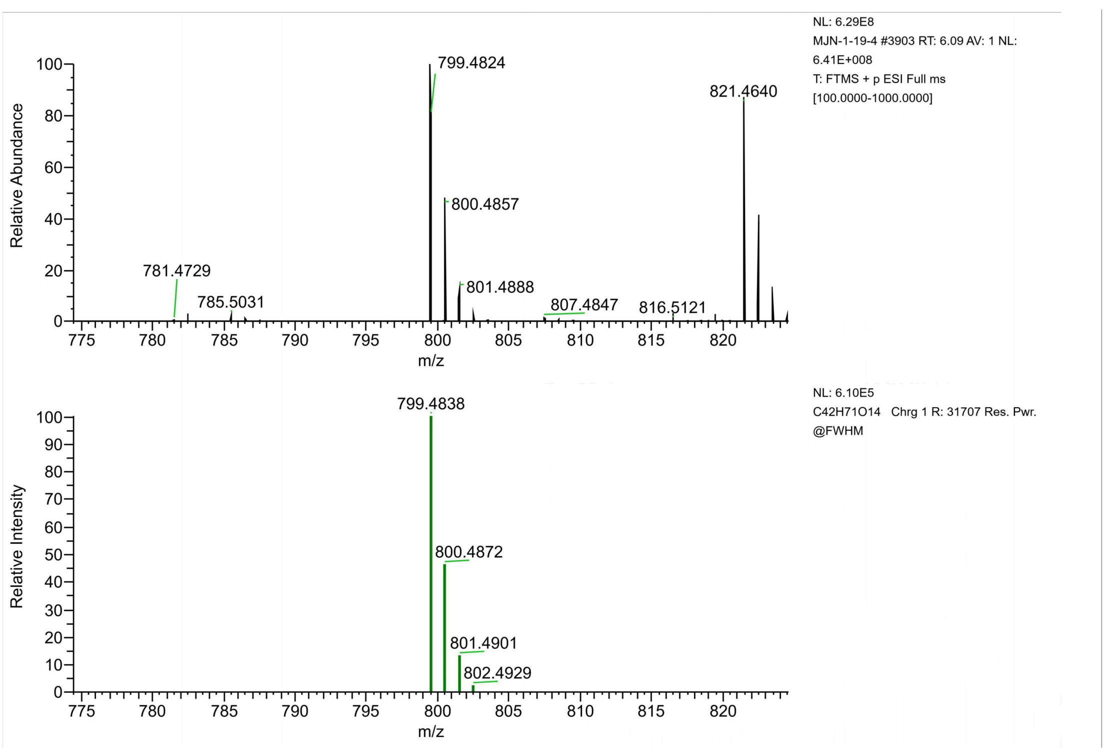

[M+Na]$^{+}$

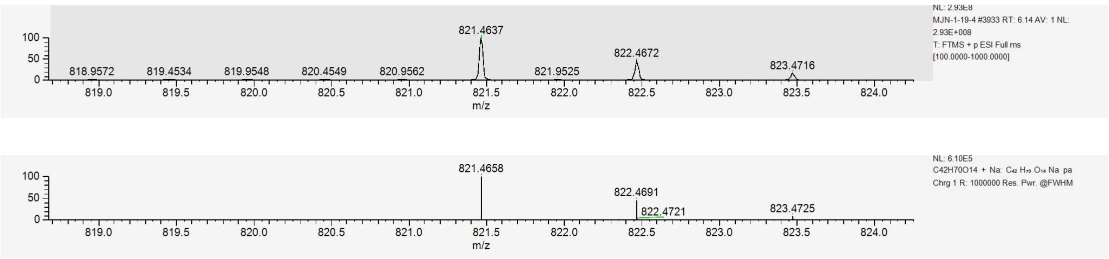

[M+H]$^{+}$

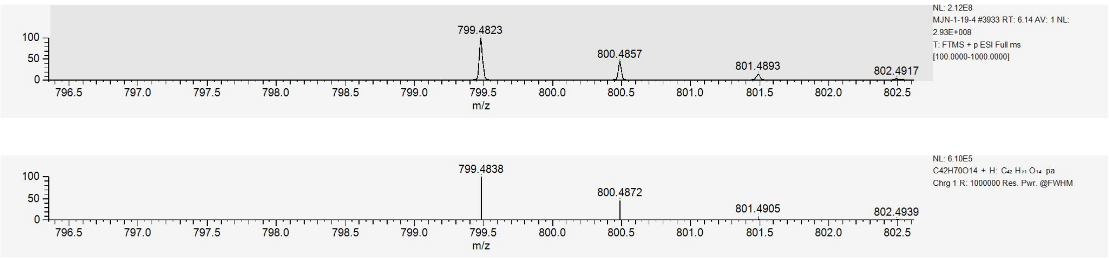

Figure S17. HRESIMS spectrum of **3** in CH_3OH

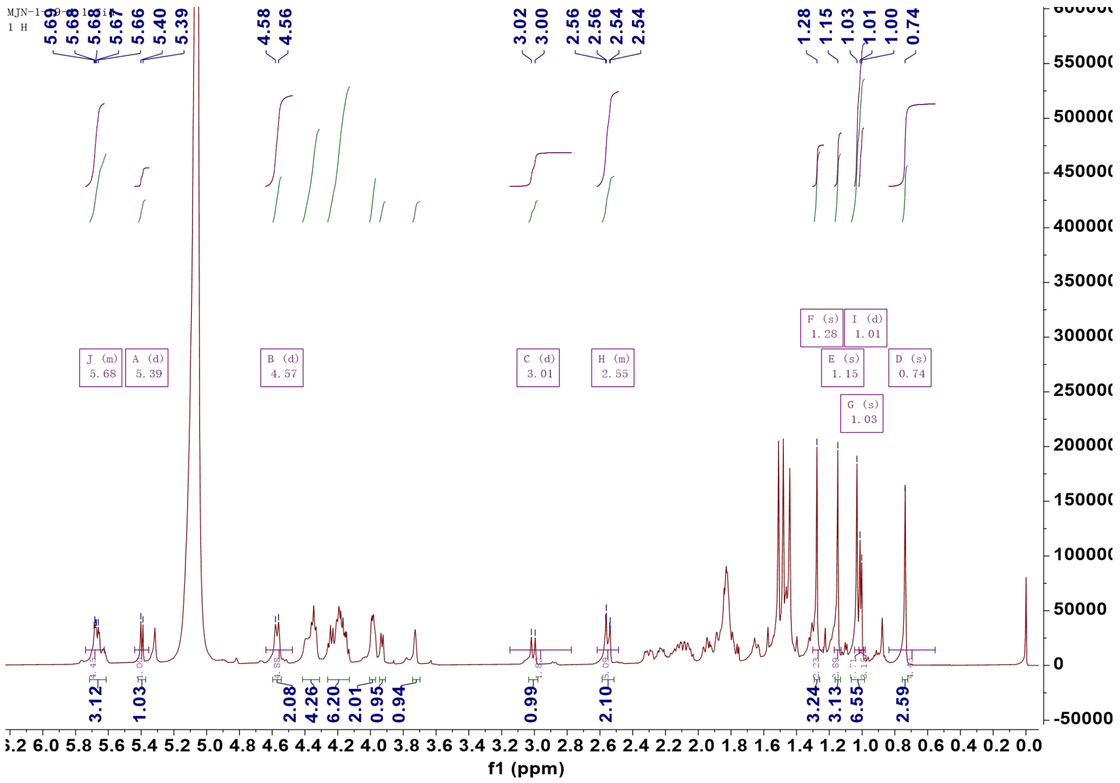

Figure S18. ^{1}H-NMR spectrum of compound **3** in PYRIDINE-d_5

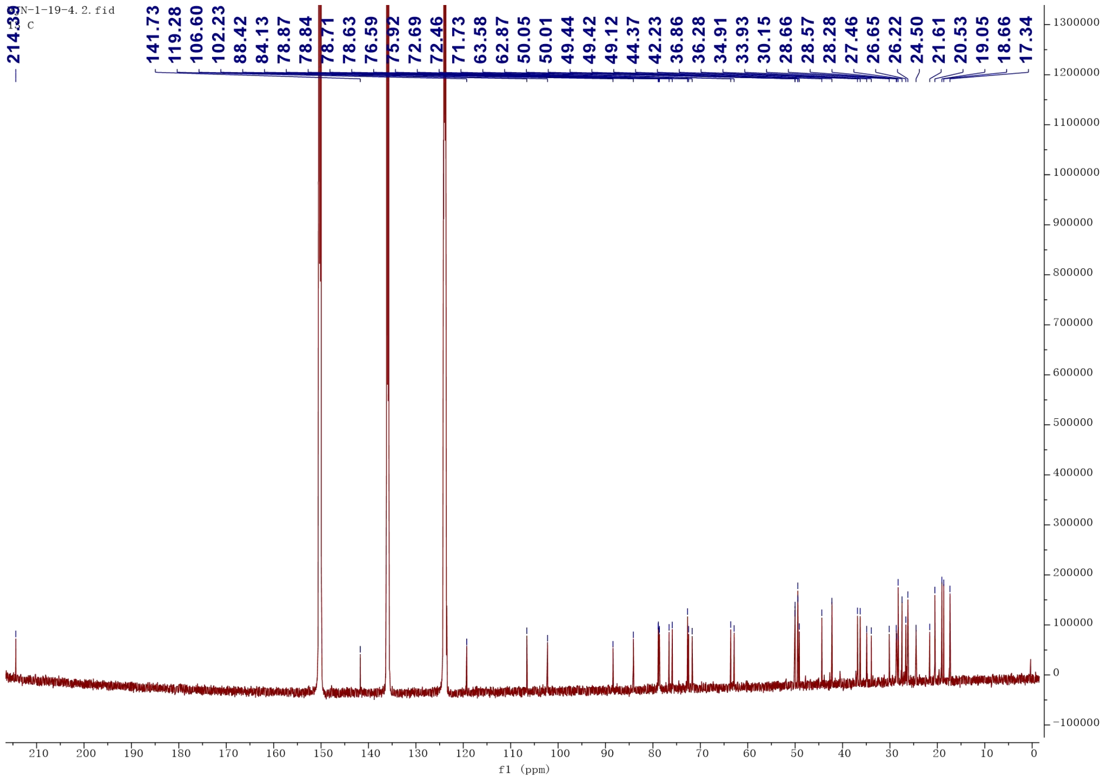

Figure S19. ^{13}C-NMR spectrum of compound **3** in PYRIDINE-d_5

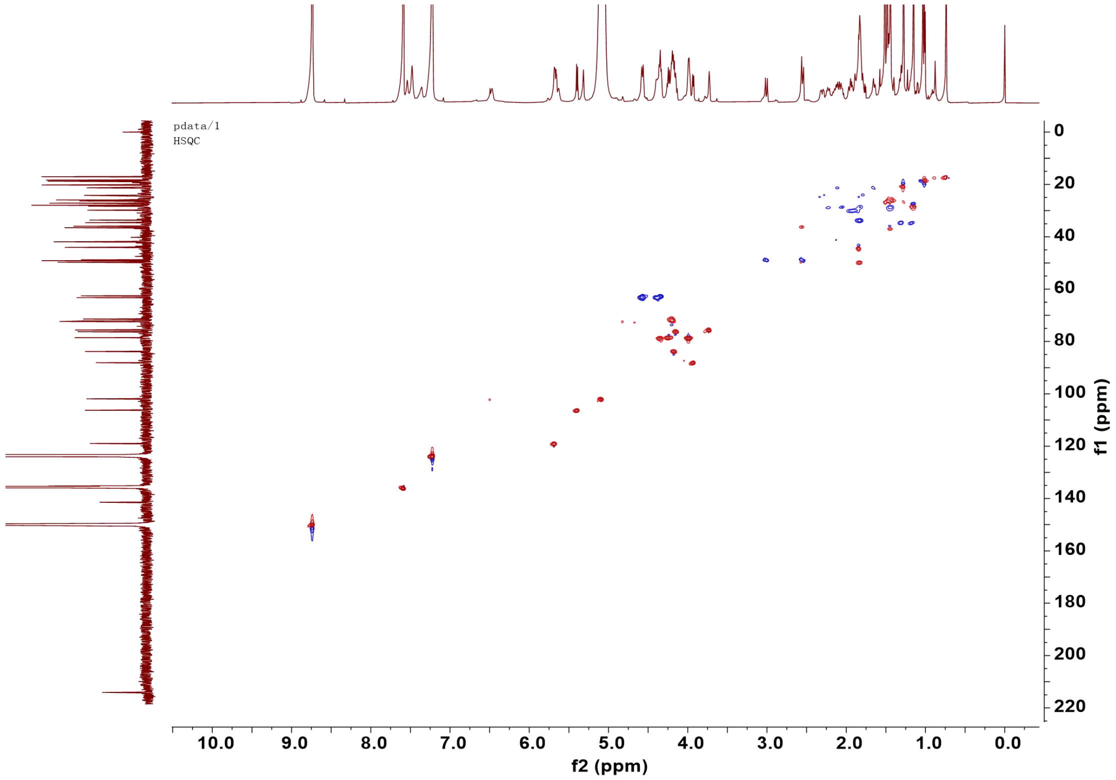

Figure S20. HSQC spectrum of compound **3** in PYRIDINE-d_5

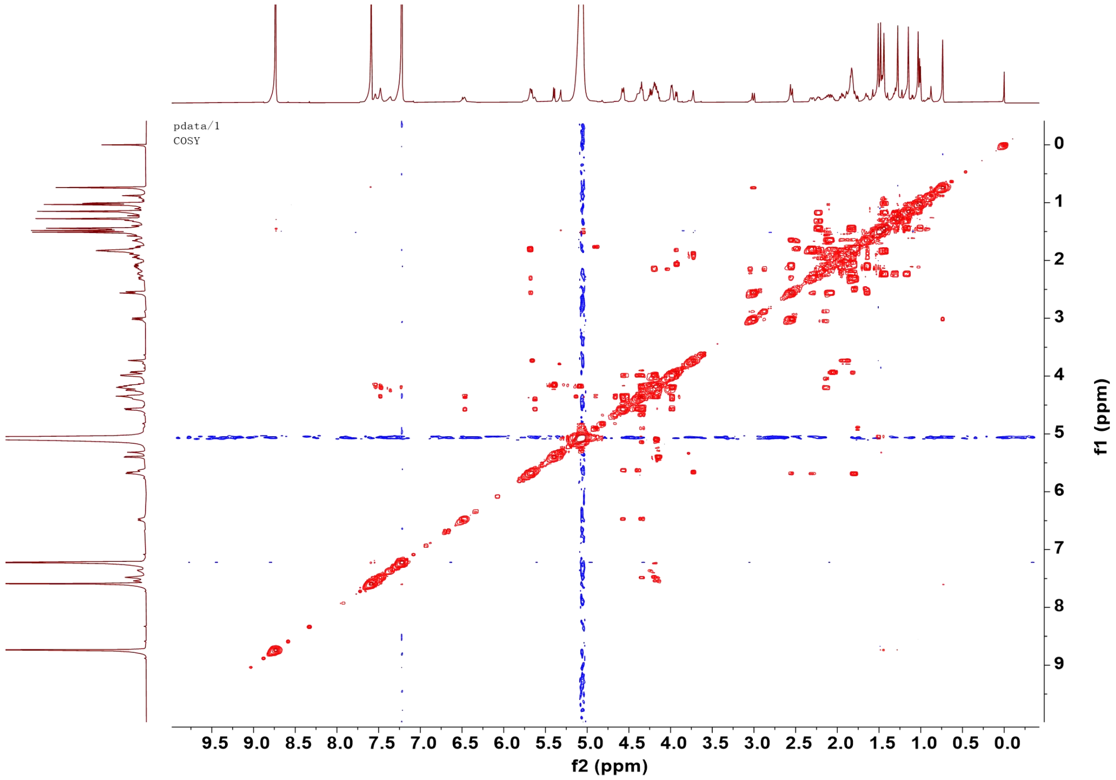

Figure S21. ^1H-^1H Cosy spectrum of **3** in PYRIDINE-d_5

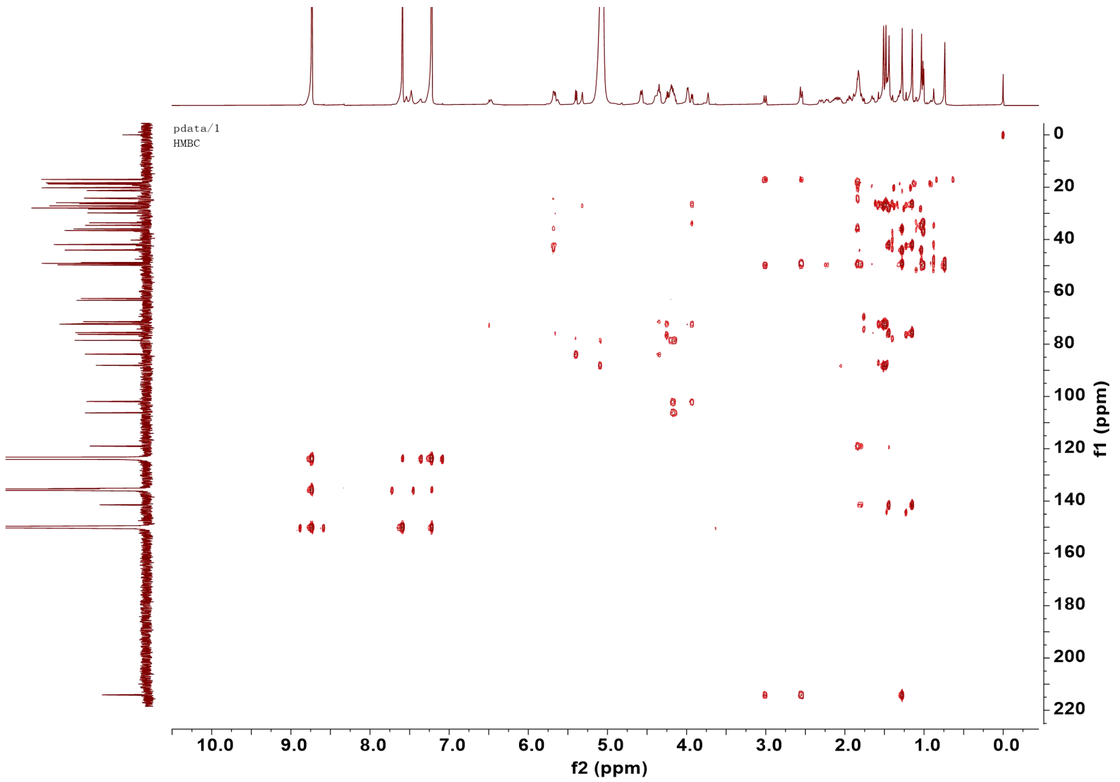

Figure S22. HMBC spectrum of **3** in PYRIDINE-d_5

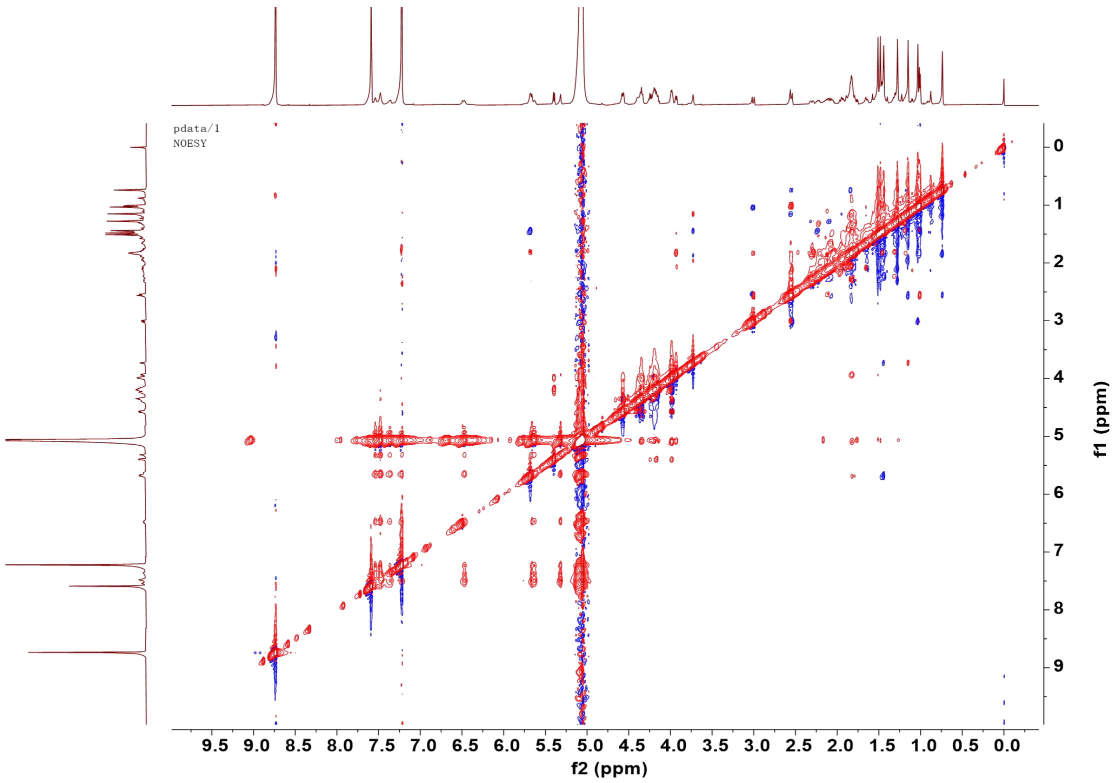

Figure S23. NOESY spectrum of **3** in PYRIDINE-d_5

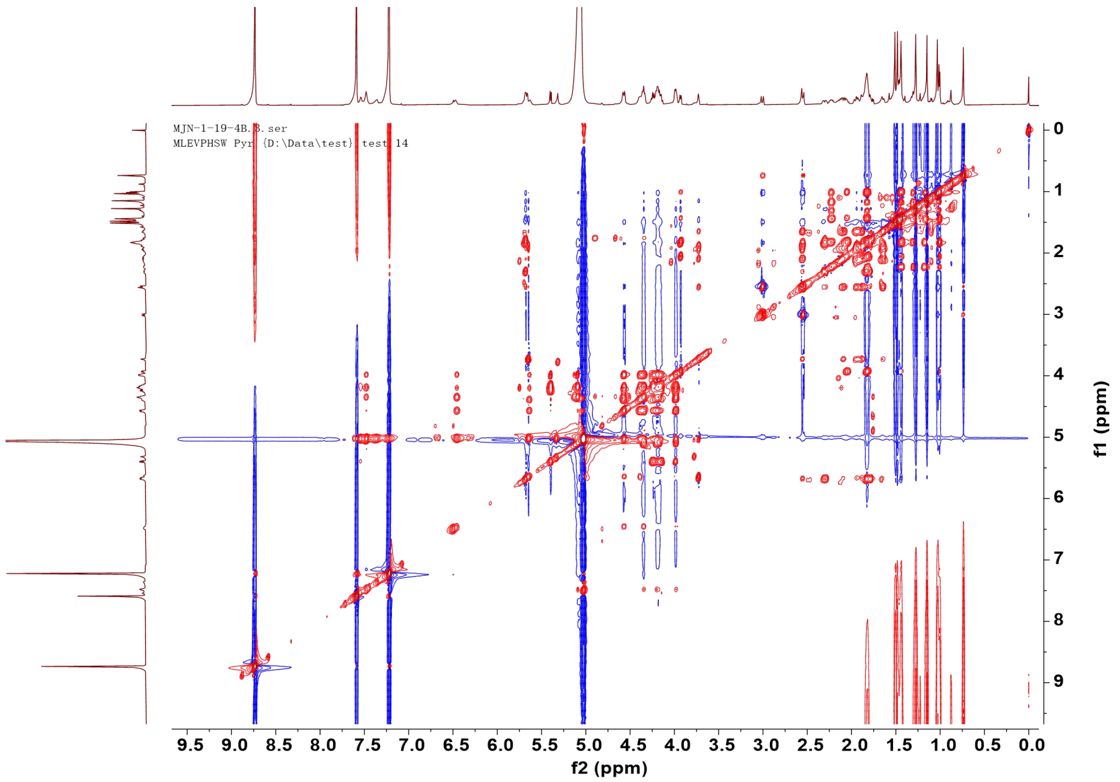

Figure S24. TOCSY spectrum of **3** in PYRIDINE-d_5

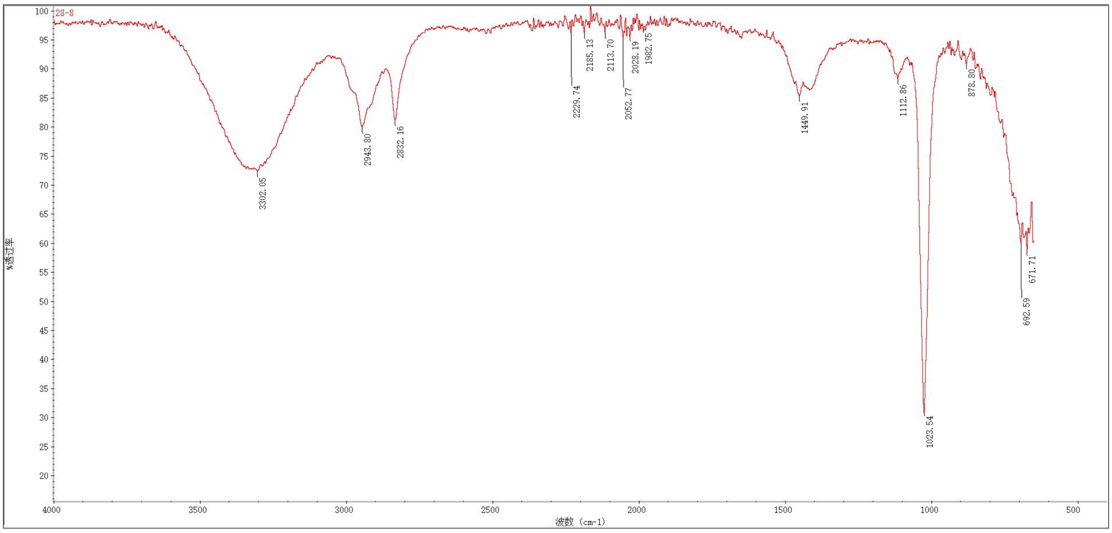

Figure S25. IR spectroscopy of **1**

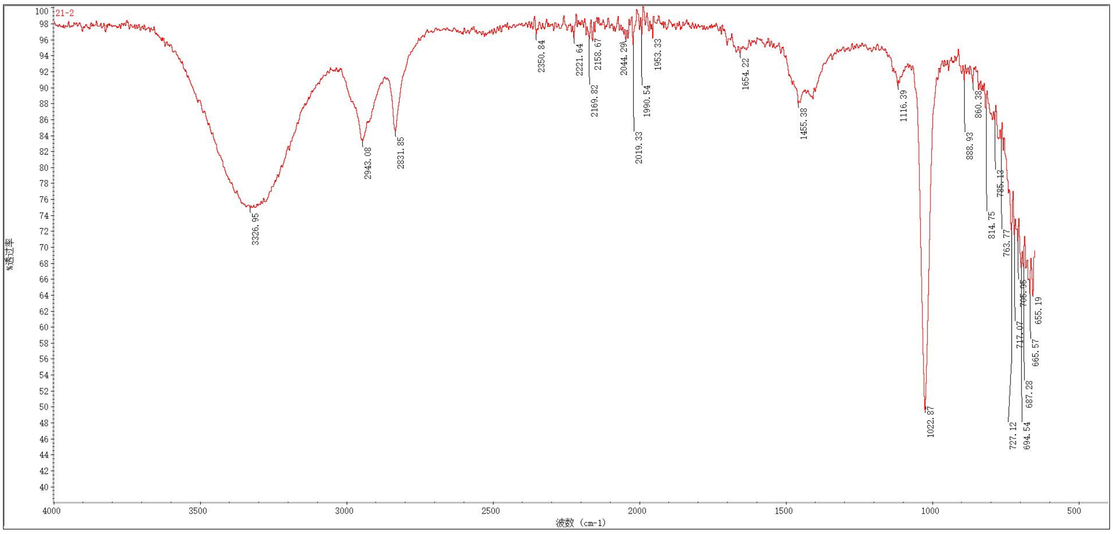

Figure S26. IR spectroscopy of **2**

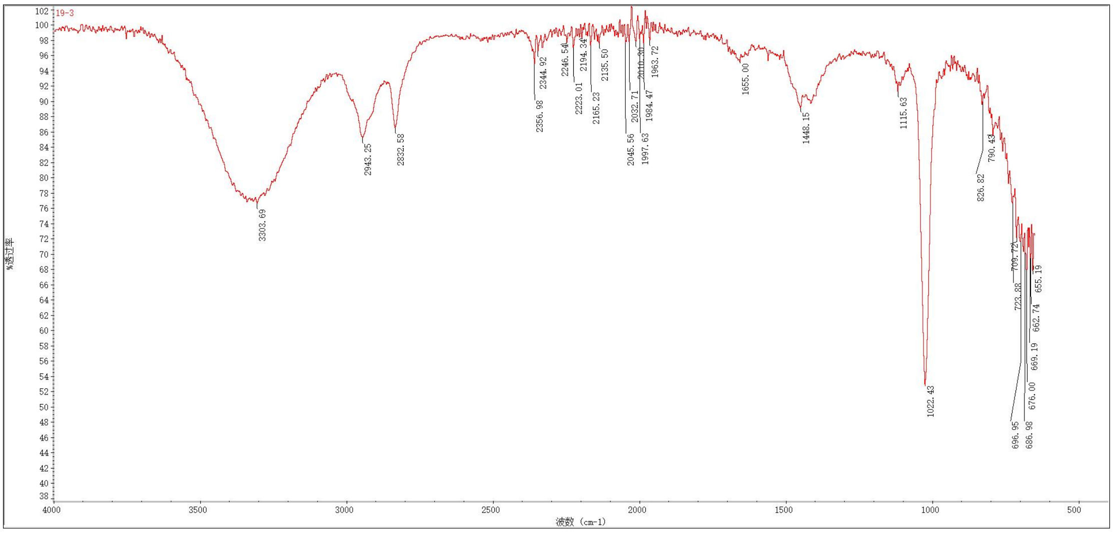

Figure S27. IR spectroscopy of **3**

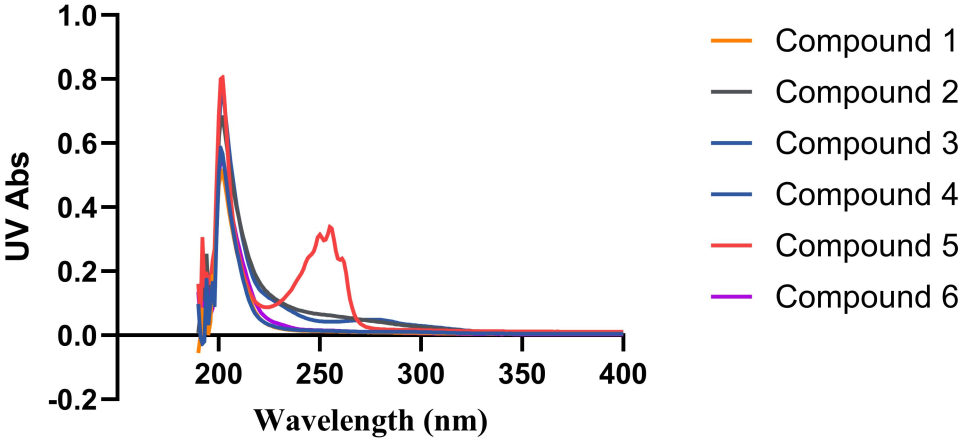

Figure S28. UV spectroscopy of **1-6**

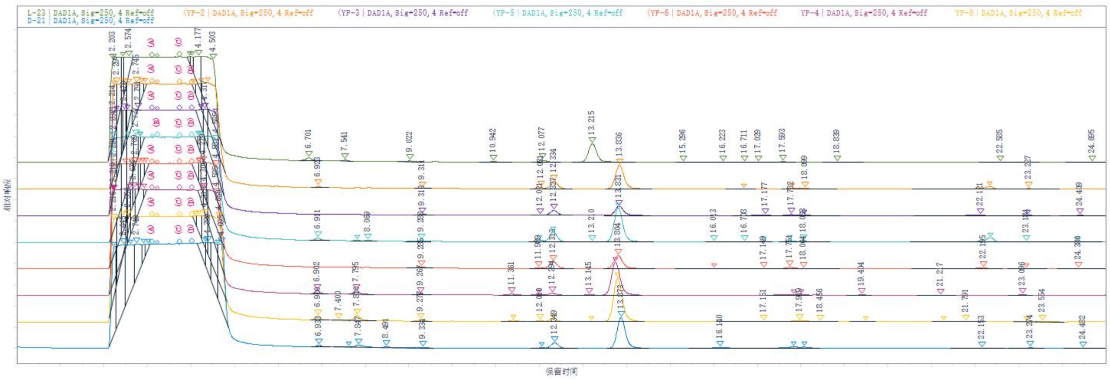

Figure S29. Glycoside Hydrolysis Experiment of **1-6**

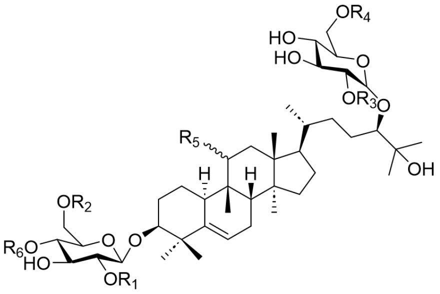

	R_1	R_2	R_3	R_4	R_5	R_6
**1**	H	-Glc	-Glc^{4}—^{1}Glc	-Glc	H_2	H
**4**	-Glc	-Glc	-Glc	-Glc	=O	H
**5**	H	-Glc	-Glc	-Glc	=O	H
**6**	H	-Glc	-Glc	-Glc	β-OH	H
**7**	H	H	-Glc	-Glc	=O	-Glc
**8**	H	H	-Rha	-Glc	α-OH	H
**9**	H	H	H	-Glc	α-OH	H
**10**	H	H	-Glc	H	=O	H
**13**	H	H	-Glc	H	α-OH	H
**15**	H	H	-Glc	-Glc	=O	H

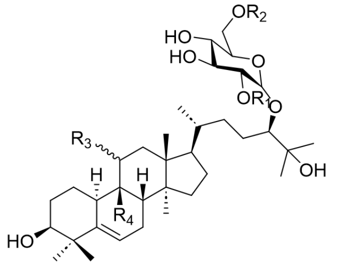

	R_1	R_2	R_3	R_4
**2**	-Glc	-Glc	=O	$-CH_2OH$
**3**	-Glc	H	=O	$-CH_3$
**11**	-Glc	-Glc	=O	$-CH_3$
**14**	-Glc	-Glc	α-OH	$-CH_3$

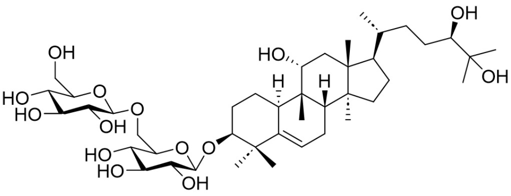

12

Figure S30. Chemical structures of compounds **1**–**15**

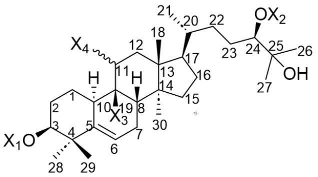

	X_1	X_2	X_3	X_4
**1**	S_2	S_4	$-CH_3$	H_2
**2**	H	S_3	$-CH_2OH$	=O
**3**	H	S_1	$-CH_3$	=O

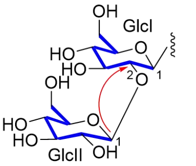

S_1

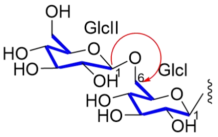

S_2

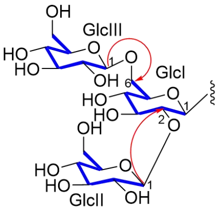

S_3

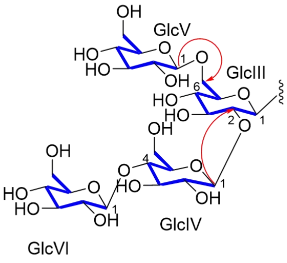

S_4

^{1}H-^{1}H COSY   HMBC

Figure S31. Structures and key 2D-NMR correlations of compounds **1–3** from *S. grosvenorii* fruit.

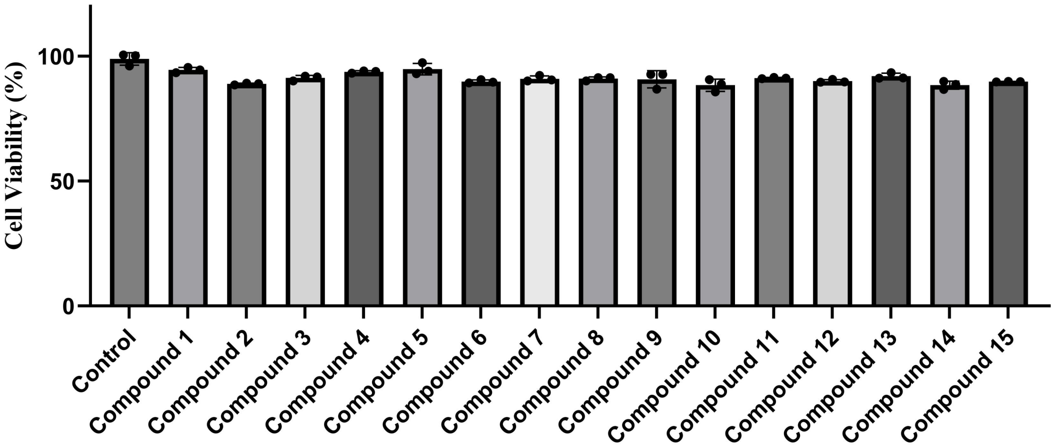

Figure S32. Cytotoxicity of compounds **1−15** (20 μM) to ALM-12 cells. Data was presented with mean± SD (n=3)

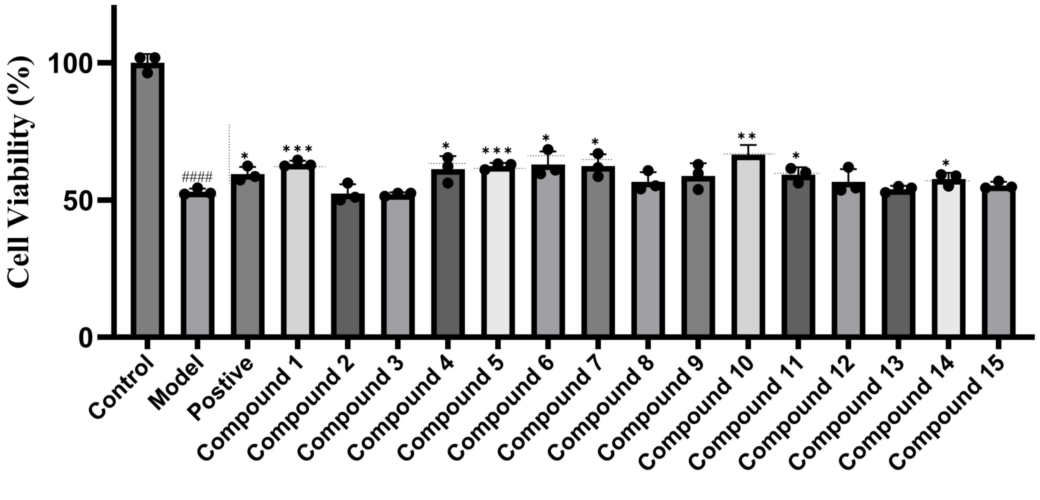

Figure S33. Hepatoprotective activity of compounds **1−15** (20 μM) on ALM-12 cells. Data was shown as mean± SD (n=3). Details: Bicyclol was used as positive control and H_2O_2 was used to cause liver damage model. Compared with control group: #### $p<0.0001$, Compared with model group: *** $p<0.001$, ** $p<0.01$, * $p<0.05$

Table S1. ^{1}H NMR (600 MHz) and ^{13}C NMR (150 MHz) data of the aglycones of compounds **1–3** in C_5D_5N (δ in ppm, *J* in Hz).

Position	**1**		**2**		**3**	
	δ_H	δ_C	δ_H	δ_C	δ_H	δ_C
1	2.02, m	26.9	1.83, m	20.5	1.66, m	21.6
	3.00, m		2.02, m		2.11, m	
2	2.23, m	29.5	1.27, m	30.2	1.85, m	30.2
	2.52, m		1.84, m		1.93, m	
3	3.69, br s	87.6	3.70, br s	75.6	3.73, br s	75.9
4	-	42.4	-	42.0	-	42.2
5	-	144.4	-	141.3	-	141.7
6	5.49, m	118.3	5.77, m	120.1	5.68, m	119.3
7	1.69, m	24.6	1.93, m	24.1	1.82, m	24.5
	2.30, m		2.03, m		2.31, m	
8	1.66, m	43.6	3.21, m	34.4	1.84, m	44.4
9	-	40.2	-	54.2	-	49.4
10	2.85, d (12.2)	36.7	2.67, d (14.7)	35.9	2.55, m	36.3
11	1.46, m	28.6	-	213.0	-	214.4
	2.12, m					
12	1.08, m	34.6	2.68, m	49.2	2.55, m	49.1
	1.15, m		3.08, m		3.01, m	
13	-	49.7	-	49.6	-	49.4
14	-	47.5	-	48.9	-	50.0
15	2.16, m	41.1	1.36, m	35.1	1.17, m	34.9
	2.21, m		1.36, m		1.31, m	
16	1.46, m	28.4	1.57, m	29.3	1.84, m	28.6
	2.12, m		2.24, m		2.05, m	
17	1.79, m	51.2	1.92, m	50.4	1.83, m	50.1
18	0.95, s	17.1	1.12, s	16.2	0.74, s	17.3
19	1.52, s	26.3	3.17, m	60.4	1.27, s	20.5
			4.96, m			
20	1.53, m	36.6	1.46, m	36.3	1.44, m	36.9
21	1.12, d	19.1	1.03, d	18.8	1.01, d	18.7

	(6.4)		(6.4)		(6.5)	
22	1.79, m	33.3	1.96, m	33.2	1.83, m	33.9
	1.87, m		1.77, m		1.83, m	
23	1.58, m	29.8	1.50, m	28.5	2.05, m	28.7
	1.89, m		1.90, m		2.22, m	
24	3.77, m	92.3	3.77, d	92.2	3.93, d	88.4
			(9.5)		(8.3)	
25	-	72.8	-	72.8	-	72.7
26	1.43, s	24.6	1.47, s	24.6	1.48, s	26.2
27	1.34, s	27.0	1.35, s	27.1	1.51, s	26.6
28	1.17, s	27.9	1.14, s	27.8	1.15, s	28.3
29	1.52, s	26.3	1.43, s	26.6	1.44, s	27.5
30	0.94, s	19.4	1.16, s	18.9	1.03, s	19.1

Table S2. ^{1}H NMR (600 MHz) and ^{13}C NMR (150 MHz) data of sugar residues of compounds **1–3** in C_5D_5N (*δ* in ppm, *J* in Hz).

Position	**1**		**2**		**3**	
	δ_H	δ_C	δ_H	δ_C	δ_H	δ_C
GlcI						
G_I-1	4.80, d (7.5)	107.0	4.93, d (7.5)	103.7	5.08, overlap	102.2
G_I-2	3.92, m	75.6	4.23, m	82.2	4.17, m	84.1
G_I-3	4.15, m	78.6	4.24, m	78.6	4.34, m	78.6
G_I-4	4.04, m	71.7	3.95, m	71.6	4.20, m	71.7
G_I-5	4.07, m	77.4	4.12, m	76.4	3.98, m	78.9
G_I-6	4.34, m	70.3	3.98, m	70.2	4.36, m	62.8
	4.80, m		4.93, m		4.57, m	
GlcII						
G_{II}-1	5.19, d (7.8)	105.4	5.54, d (7.8)	105.5	5.40, d (7.7)	106.6
G_{II}-2	4.05, m	75.3	4.13, m	75.6	4.15, m	76.6
G_{II}-3	4.27, m	78.2	4.25, m	78.4	4.25, m	78.7
G_{II}-4	4.26, m	71.5	4.15, m	72.5	4.19, m	72.5
G_{II}-5	3.97, m	78.5	3.97, m	78.2	3.98, m	78.8
G_{II}-6	4.40, m	62.6	4.35, m	63.5	4.40, m	63.6
	4.53, m		4.54, m		4.57, m	
GlcIII						
G_{III}-1	4.93, d (7.6)	103.7	4.88, d (7.7)	104.9		
G_{III}-2	4.15, m	82.6	4.08, m	75.5		
G_{III}-3	4.23, m	76.3	4.27, m	78.1		
G_{III}-4	3.94, m	71.5	4.27, m	71.5		
G_{III}-5	4.08, m	76.4	3.93, m	78.8		
G_{III}-6	3.98, m	70.2	4.37, m	62.6		
	4.92, m		4.52, m			
GlcIV						
G_{IV}-1	5.44, d (7.9)	105.4				

G_{IV}-2	4.11, m	75.4
G_{IV}-3	4.24, m	76.7
G_{IV}-4	4.23, m	82.4
G_{IV}-5	3.94, m	78.6
G_{IV}-6	4.48, m	63.2
	4.50, m	
GlcV		
G_{V}-1	4.88, d (7.6)	104.9
G_{V}-2	4.07, m	75.6
G_{V}-3	4.27, m	78.3
G_{V}-4	4.26, m	71.7
G_{V}-5	3.93, m	78.5
G_{V}-6	4.31, m	62.5
	4.55, m	
GlcVI		
G_{VI}-1	5.13, d (7.9)	105.0
G_{VI}-2	4.09, m	75.4
G_{VI}-3	4.20, m	78.1
G_{VI}-4	4.23, m	71.5
G_{VI}-5	4.01, m	78.1
G_{VI}-6	4.31, m	62.5
	4.55, m	

Appendix A: 1D-NMR, 2D-NMR and HRESIMS of compounds **4-15**
Compound **4**

HRESIMS

$[M+H]^+$

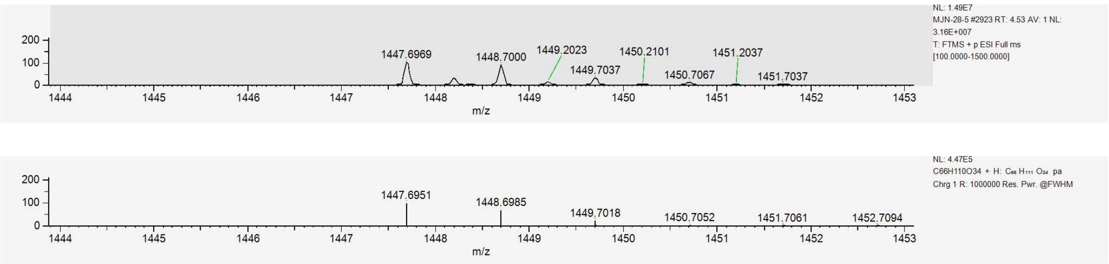

$[M-H]^-$

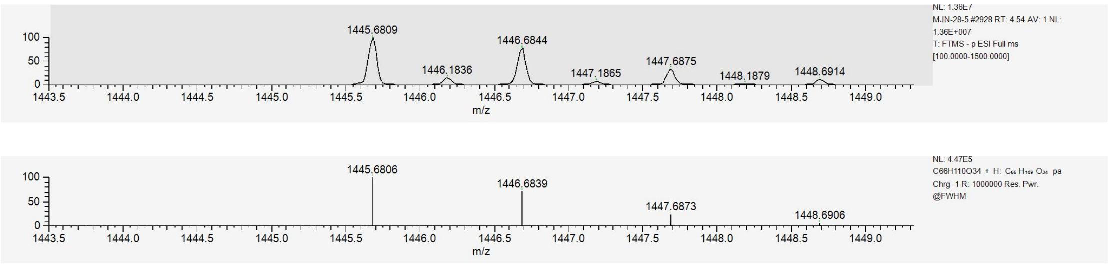

$[M+Na]^+$

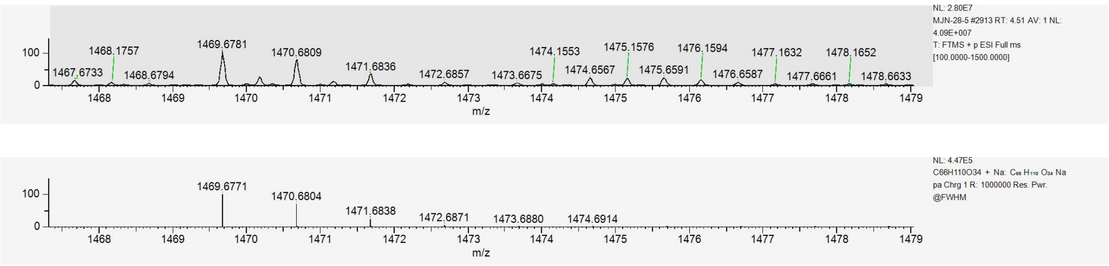

^{1}H-NMR

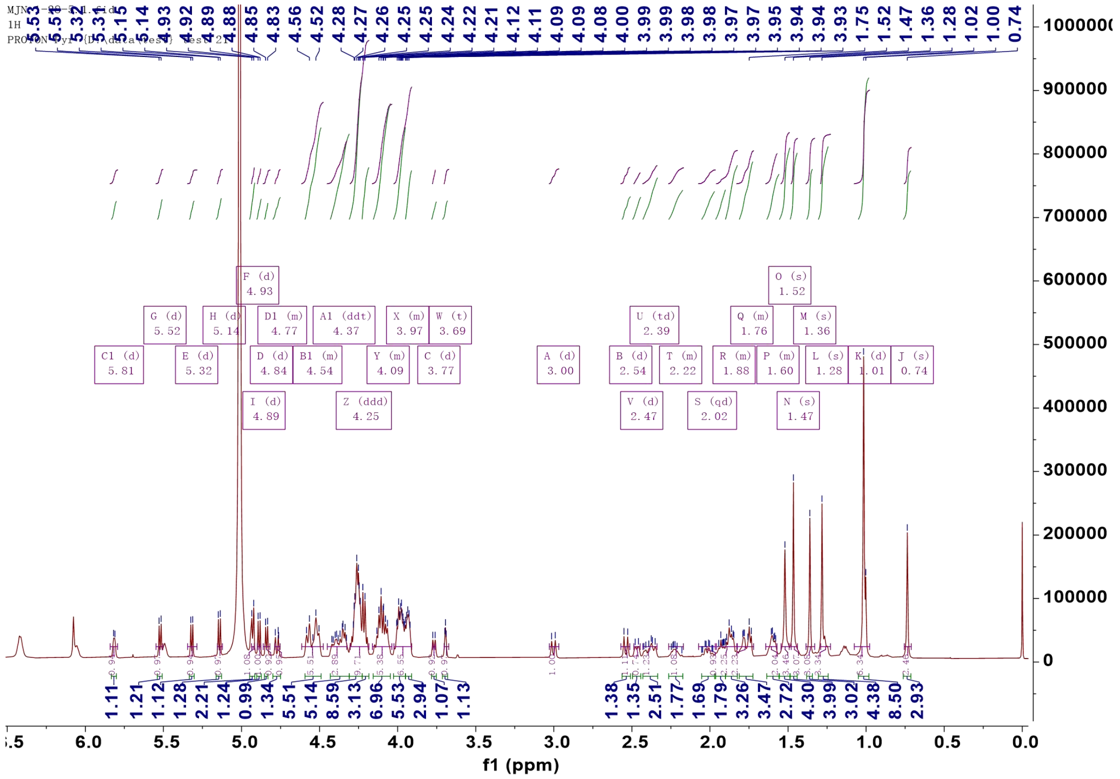

^{13}C-NMR

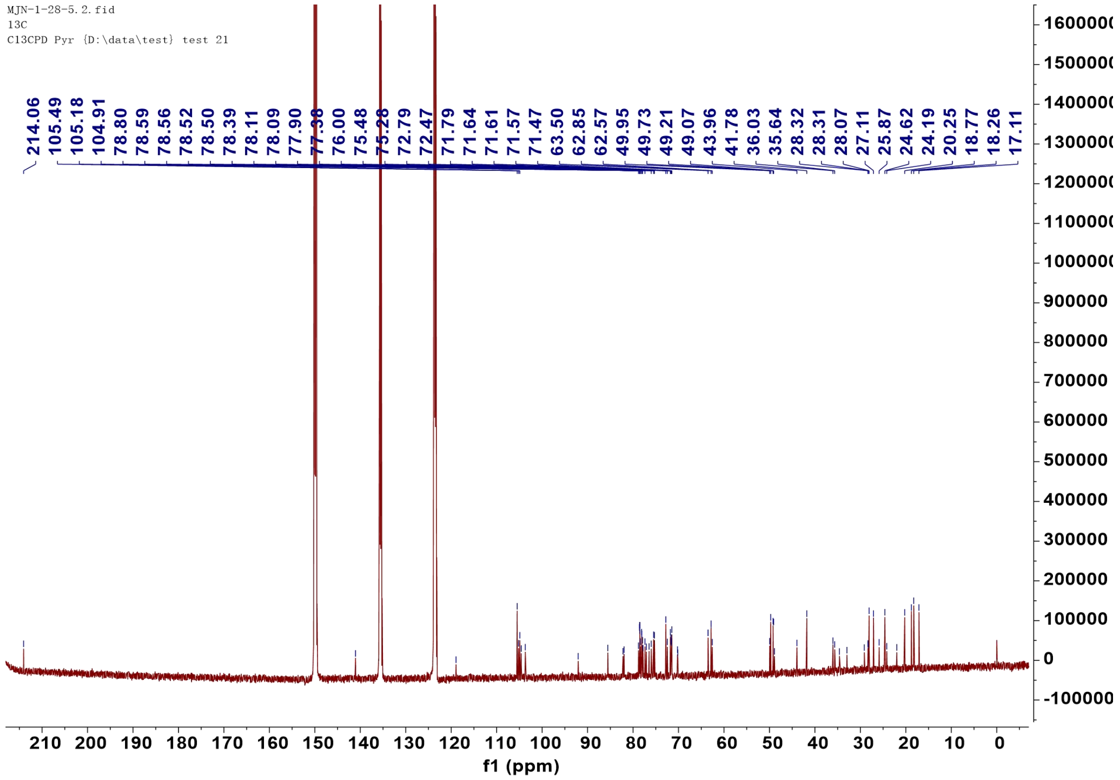

HSQC

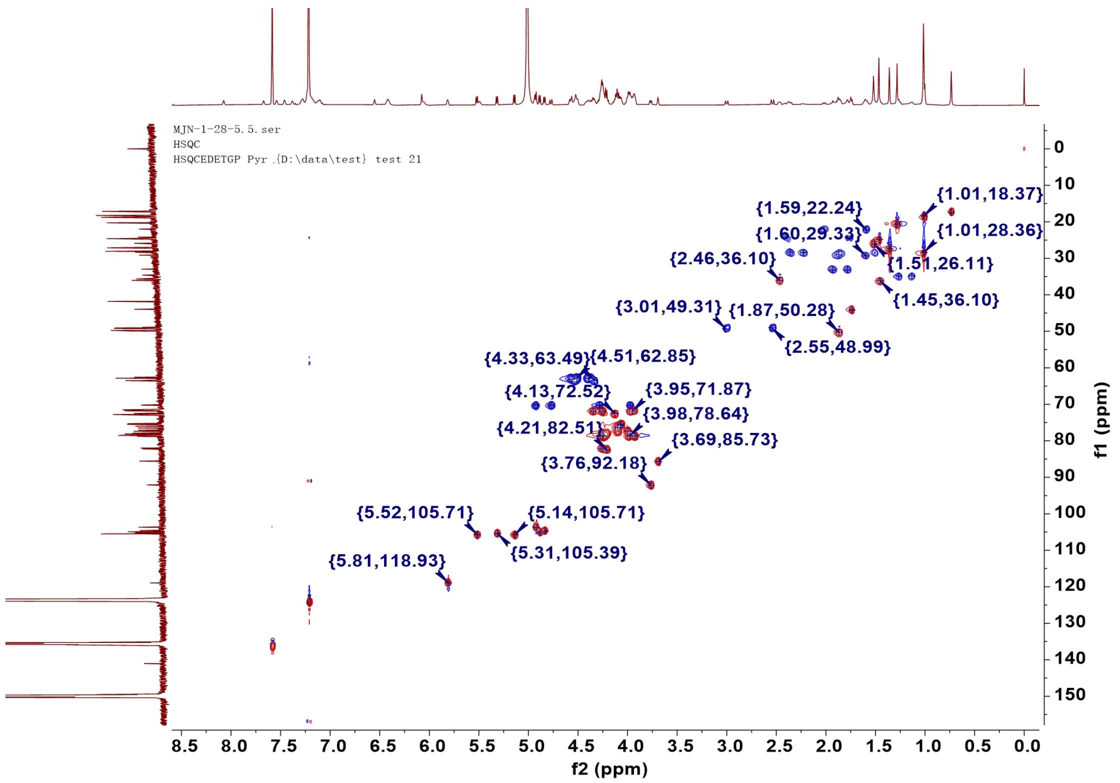

^1H-^1H Cosy

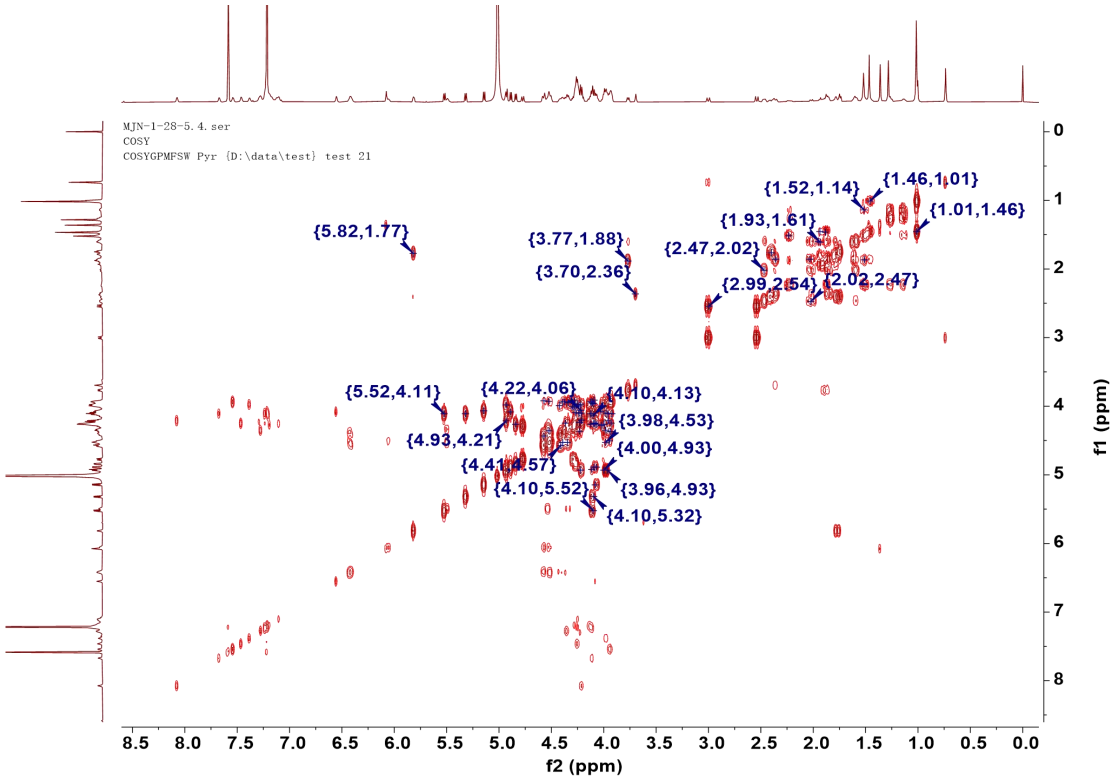

HMBC

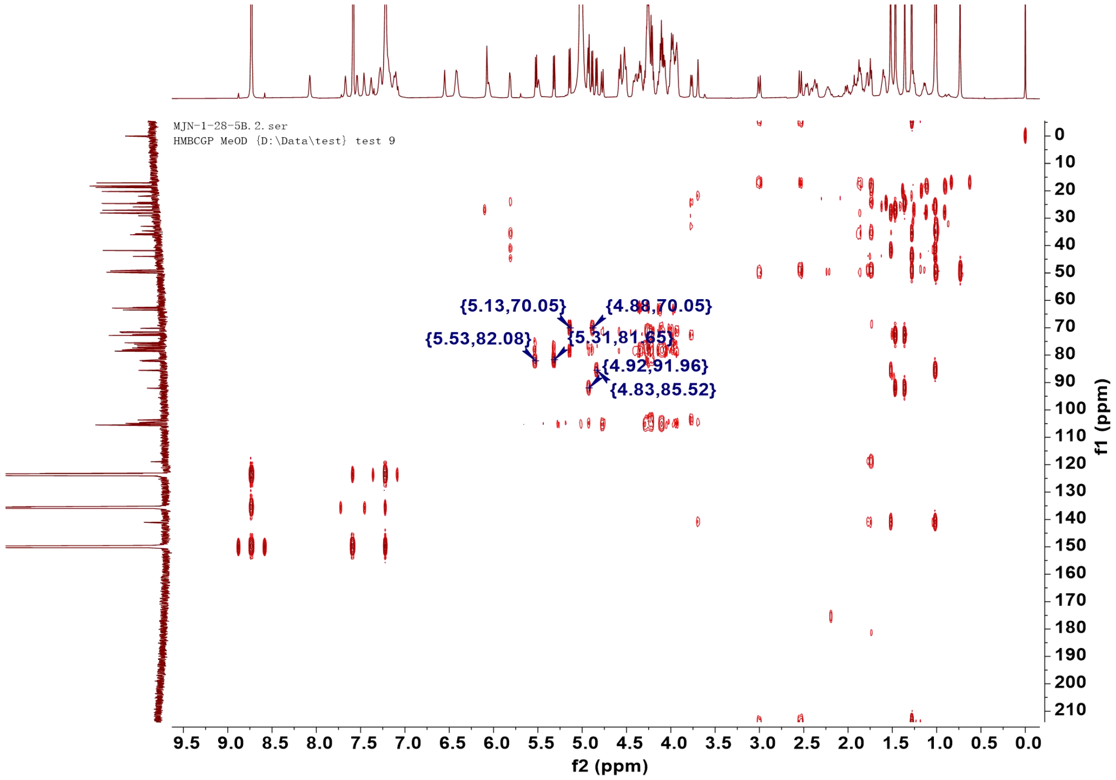

MJN-1-28-5B.2.ser
HMBCGP MeOD {D:\Data\test} test 9
{5.13,70.05}
{4.88,70.05}
{5.53,82.08}
{5.31,81.65}
{4.92,91.96}
{4.83,85.52}
f2 (ppm)
f1 (ppm)

NOESY

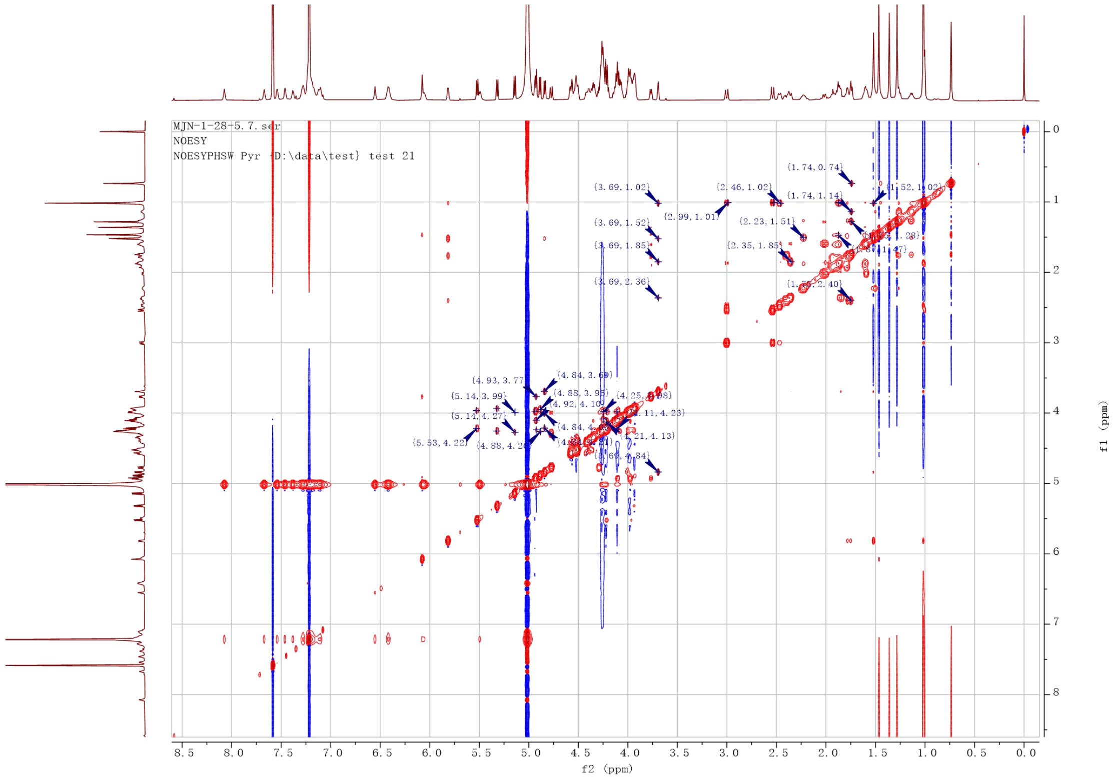

MJN-1-28-5.7.ser
NOESY
NOESYPHSW Pyr {D:\data\test} test 21
f2 (ppm)
f1 (ppm)

Compound **5**

HRESIMS

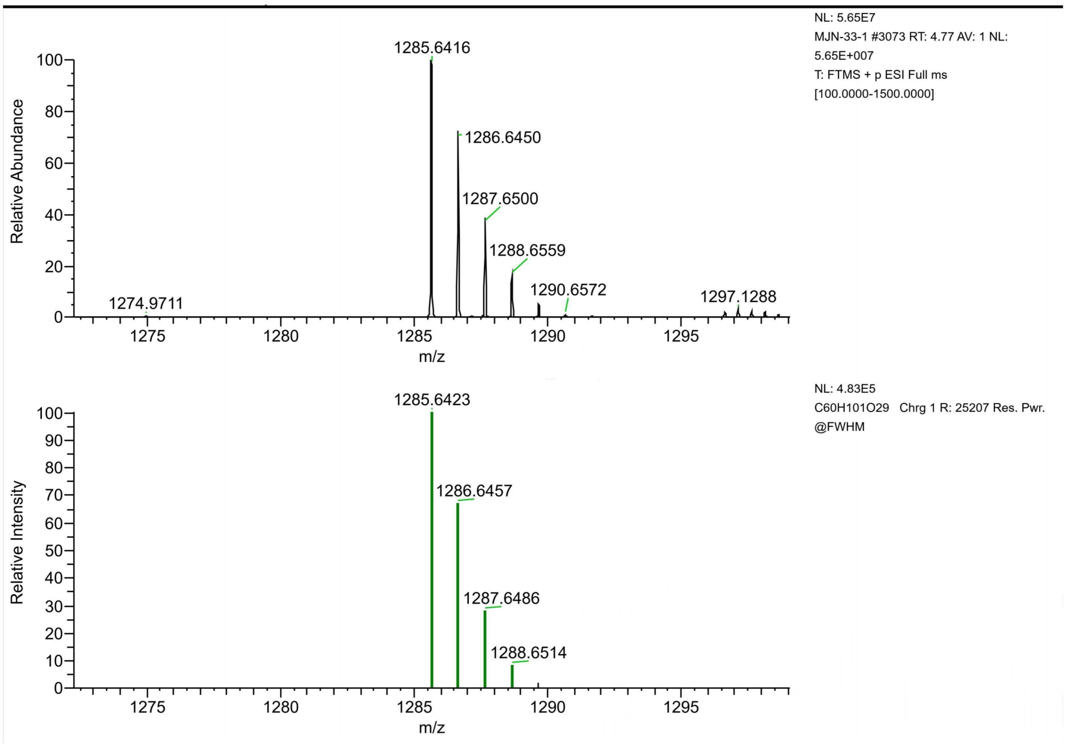

[M+Na]$^{+}$

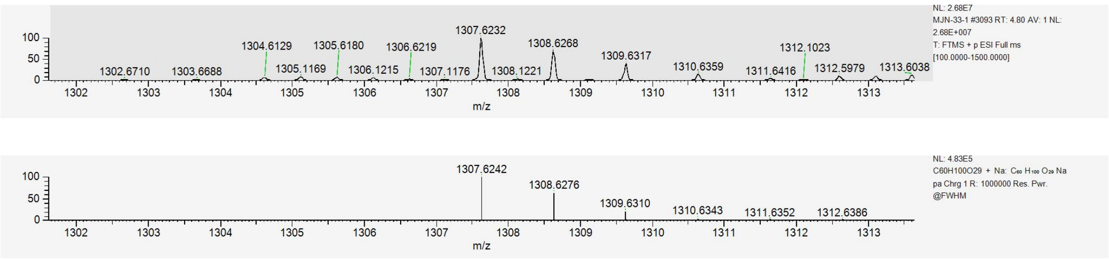

[M+H]$^{+}$

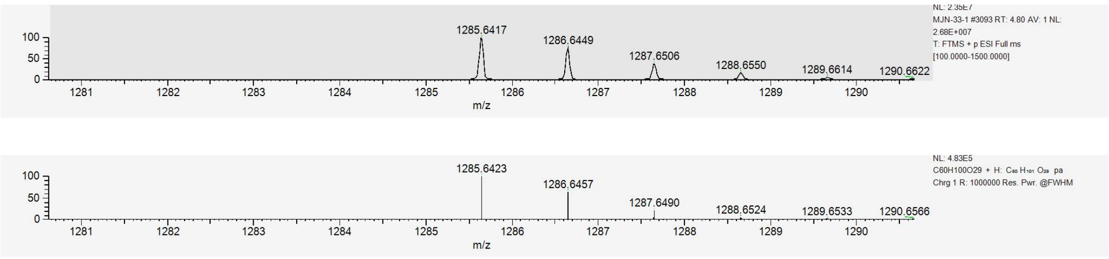

^{1}H-NMR

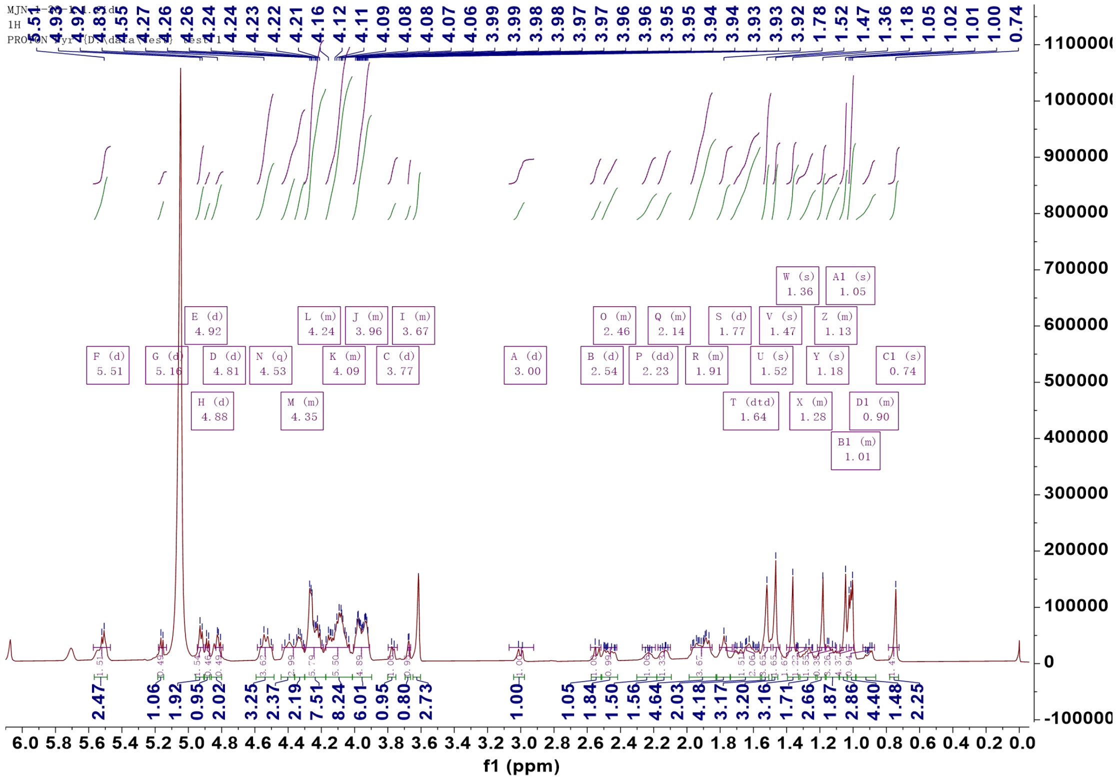

^{13}C-NMR

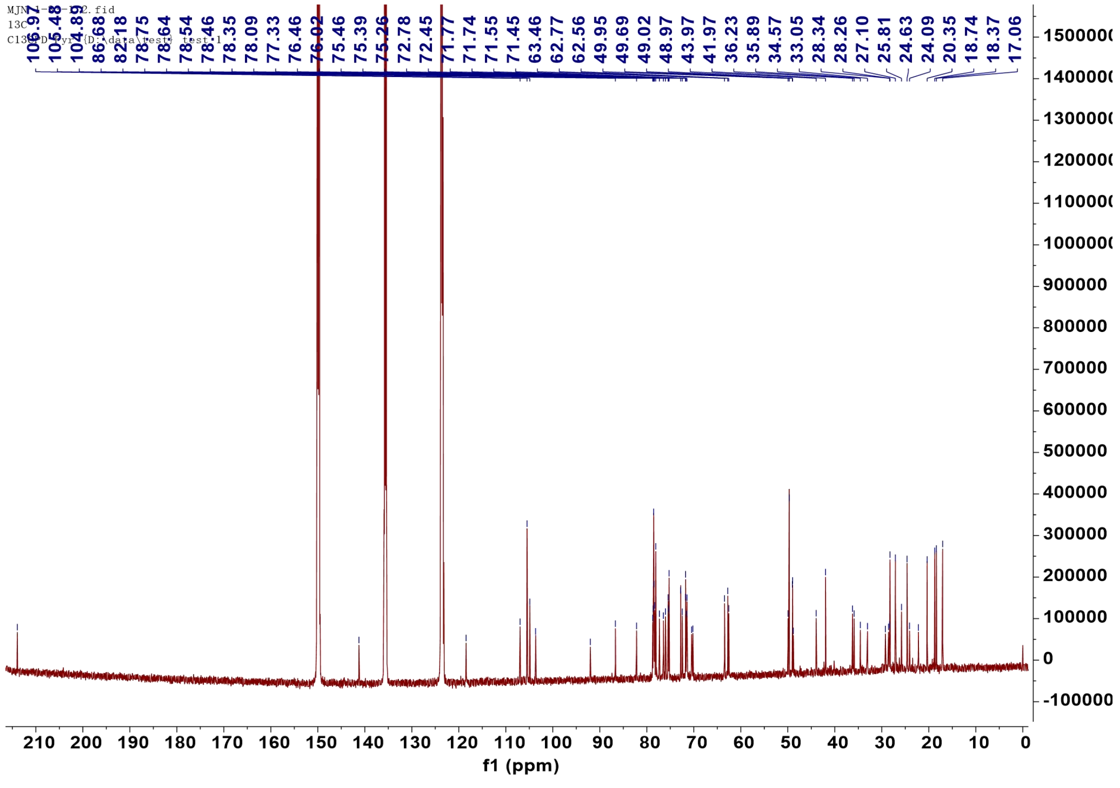

HSQC

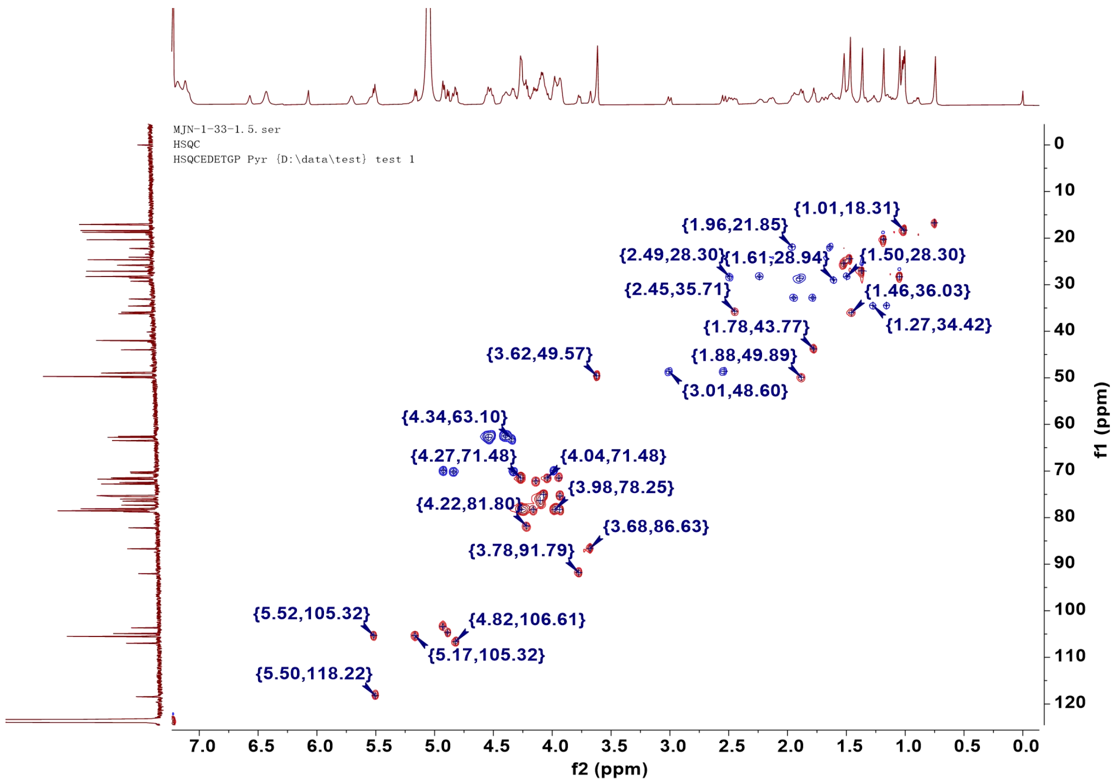

^{1}H-^{1}H Cosy

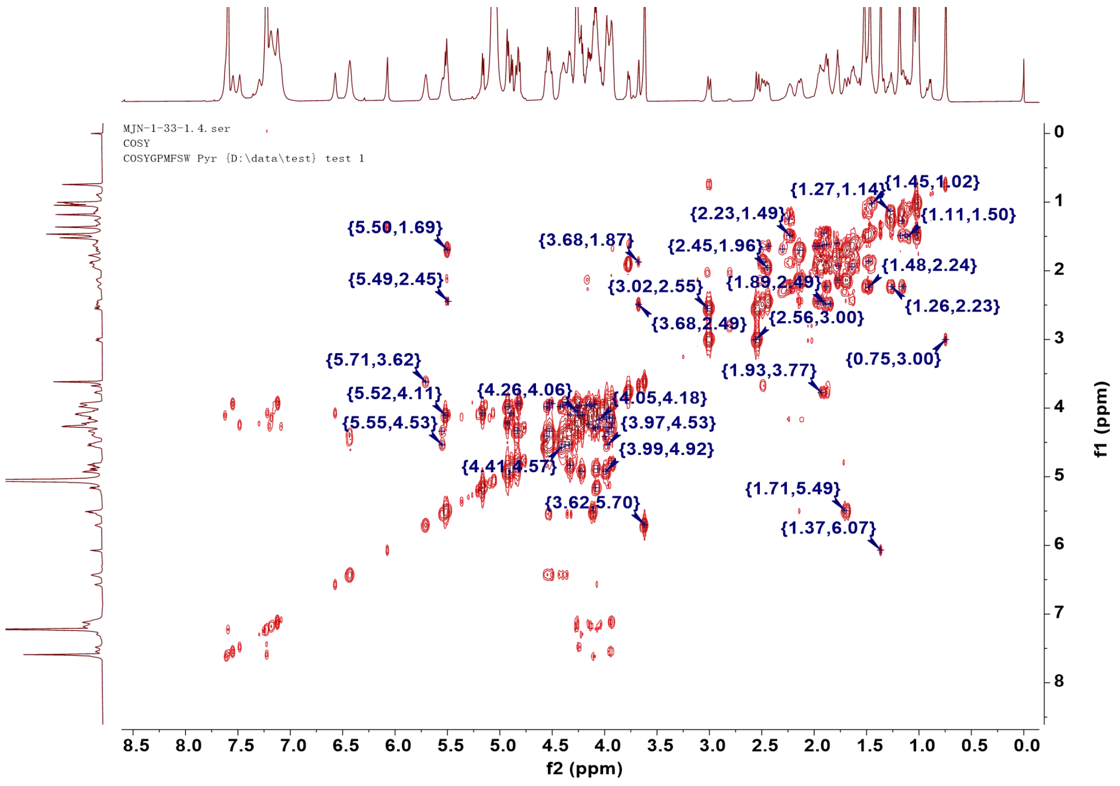

HMBC

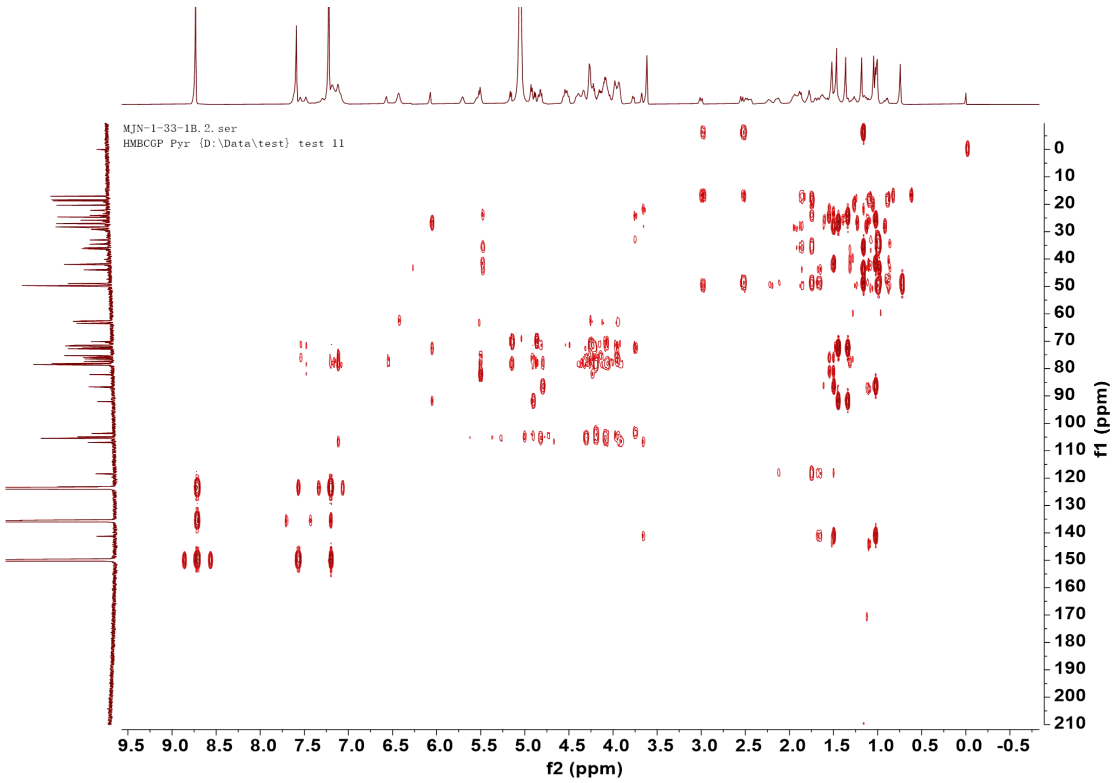

MJN-1-33-1B. 2. ser
HMBCGP Pyr {D:\Data\test} test 11
f1 (ppm)
f2 (ppm)

NOESY

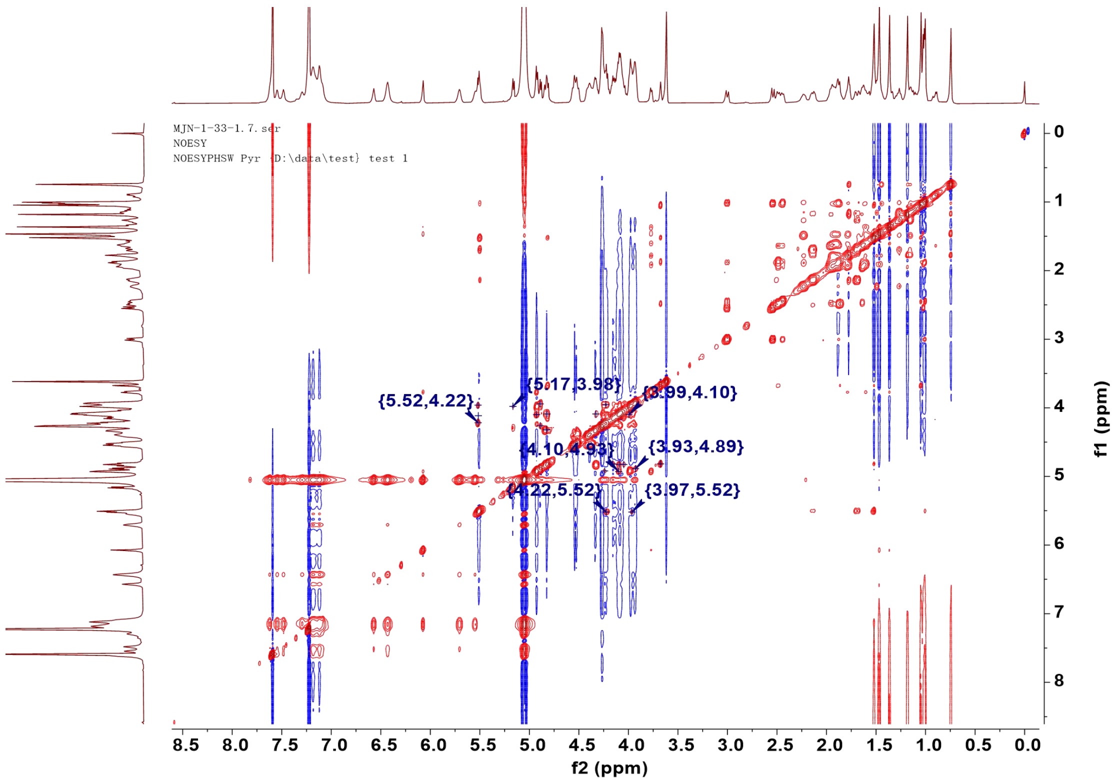

MJN-1-33-1. 7. ser
NOESY
NOESYPHSW Pyr {D:\data\test} test 1
{5.52,4.22}
{5.17,3.98}
{3.99,4.10}
{4.10,4.93}
{3.93,4.89}
{4.22,5.52}
{3.97,5.52}
f1 (ppm)
f2 (ppm)

Compound **6**

HRESIMS

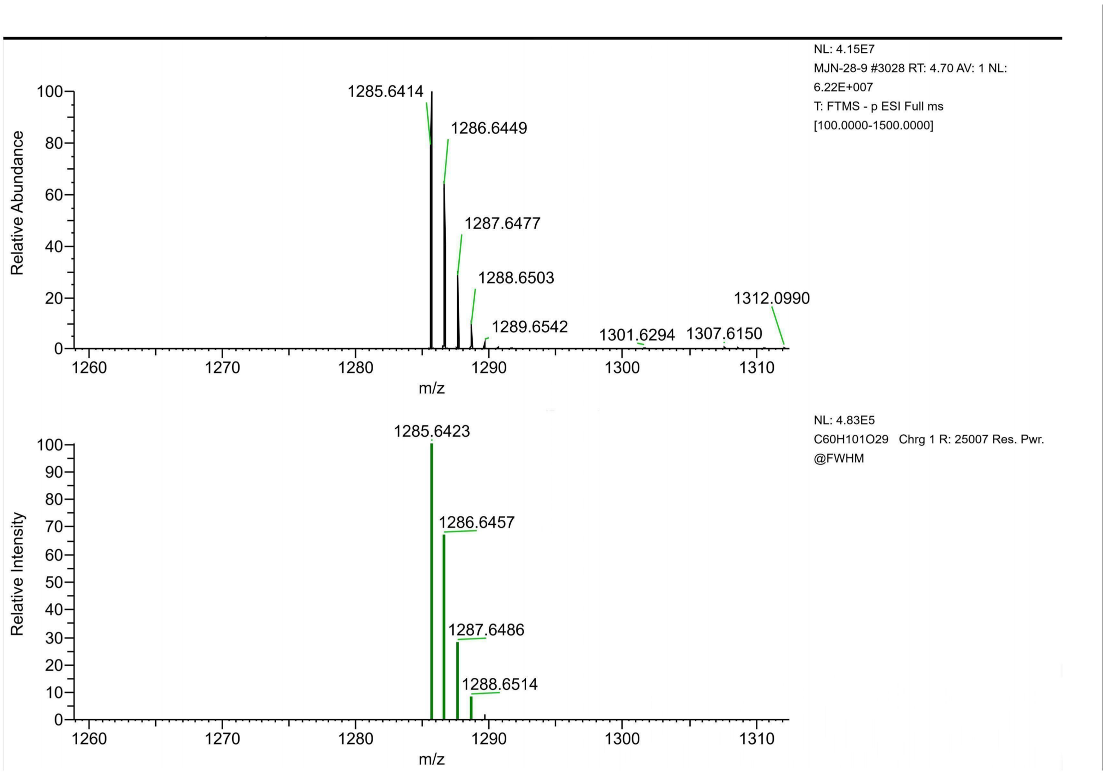

[M-H]$^-$

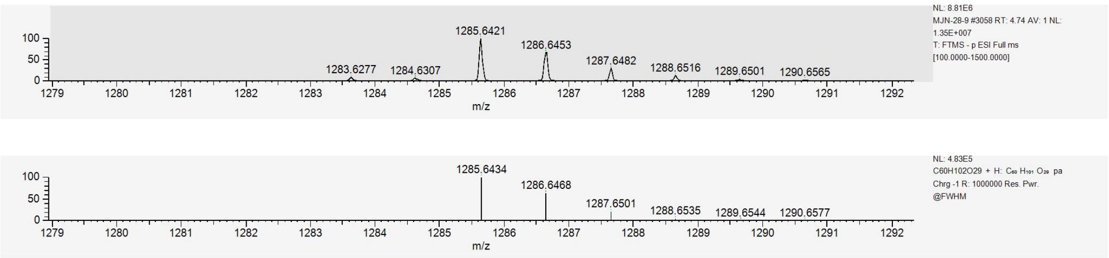

^{1}H-NMR

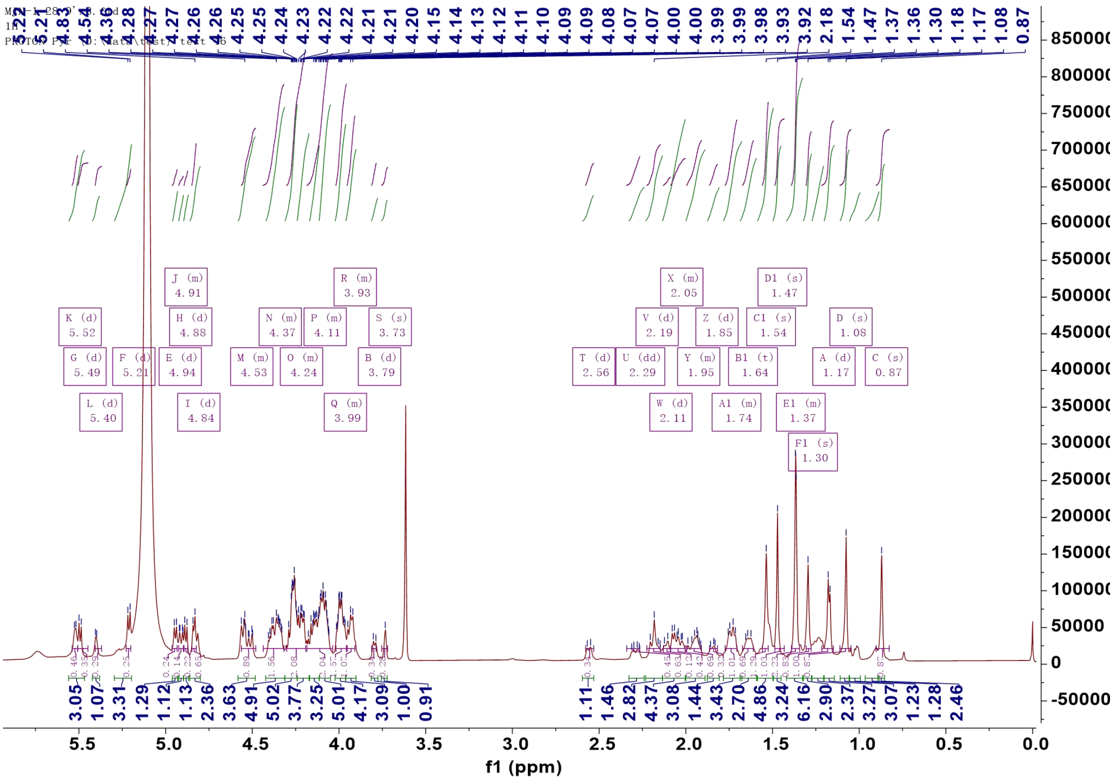

^{13}C-NMR

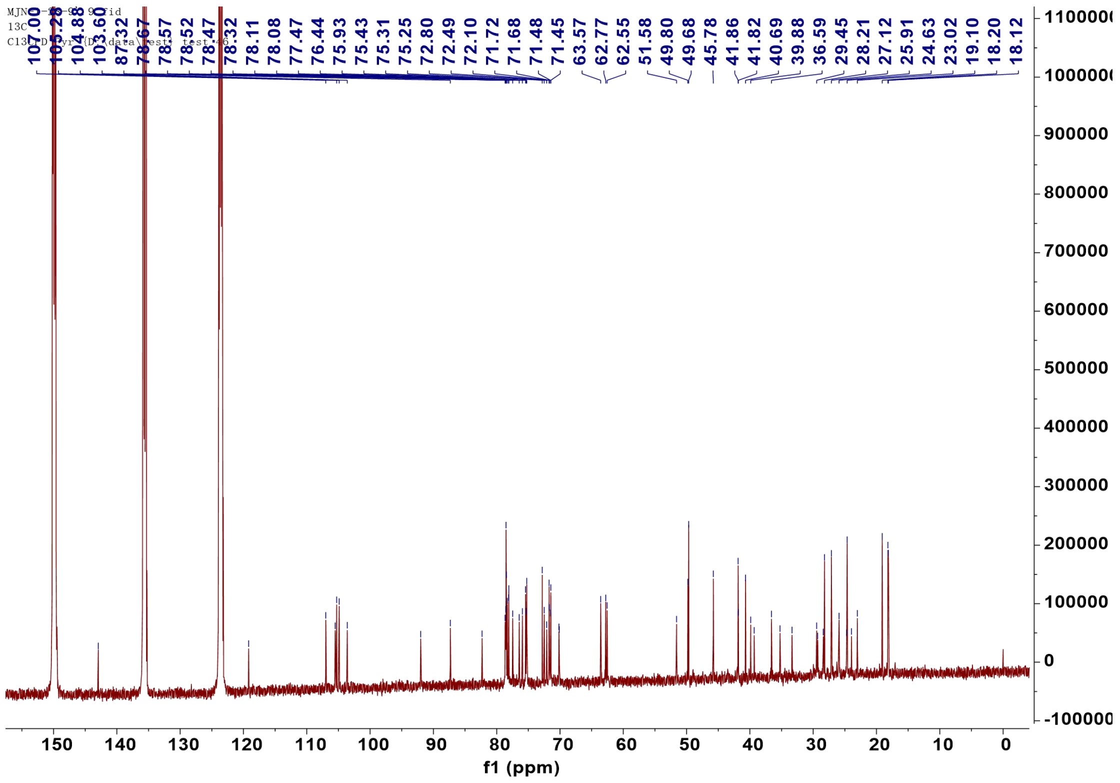

HSQC

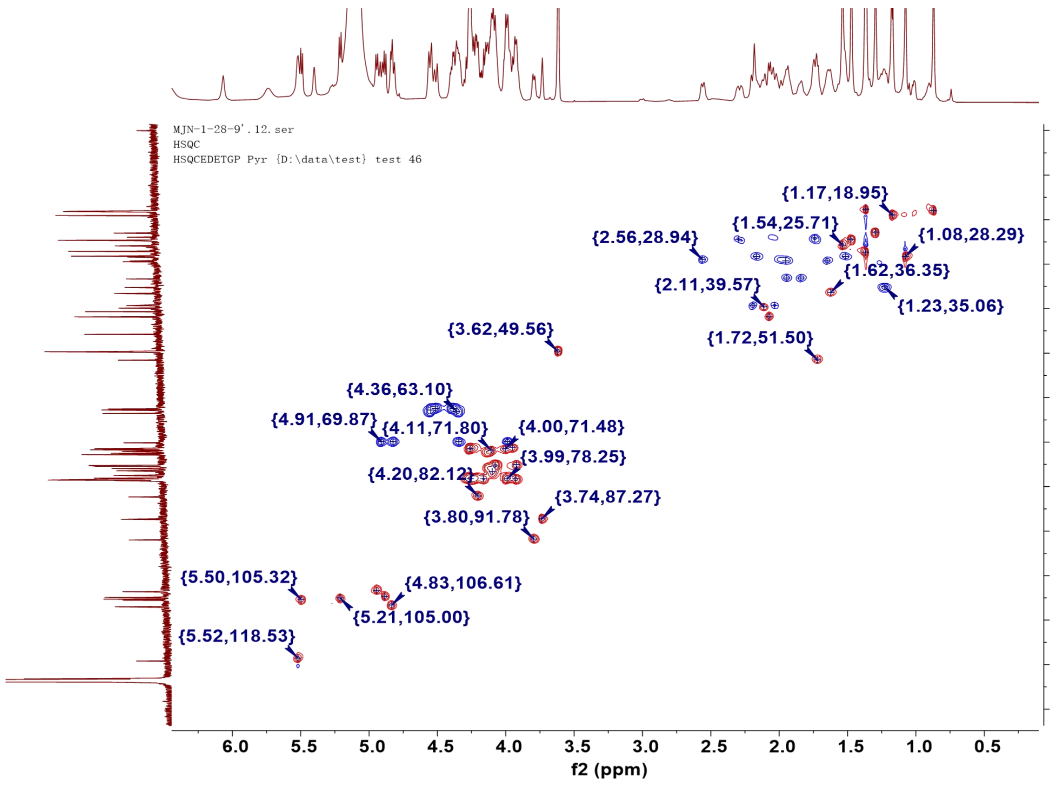

^{1}H-^{1}H Cosy

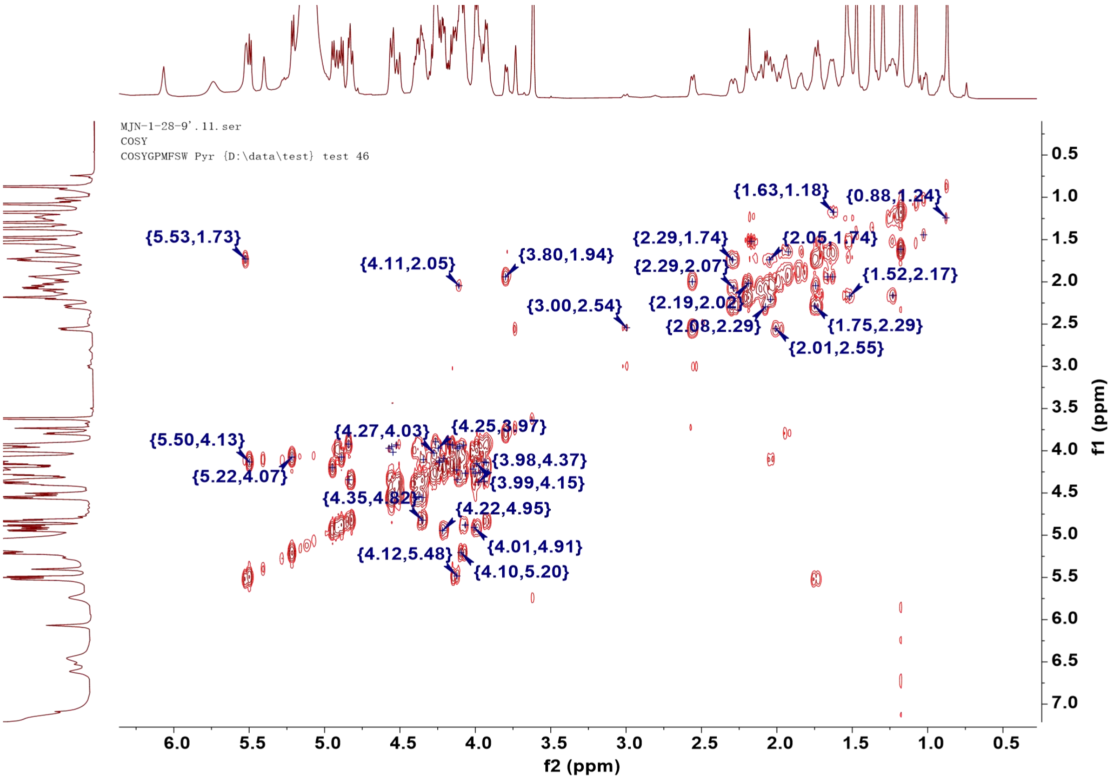
MJN-1-28-9'.11.ser
COSY
COSYGPMFSW Pyr {D:\data\test} test 46
{1.63,1.18}
{0.88,1.24}
{5.53,1.73}
{2.29,1.74}
{2.05,1.74}
{4.11,2.05}
{3.80,1.94}
{2.29,2.07}
{1.52,2.17}
{3.00,2.54}
{2.19,2.02}
{2.08,2.29}
{1.75,2.29}
{2.01,2.55}
{4.27,4.03}
{4.25,3.97}
{5.50,4.13}
{3.98,4.37}
{5.22,4.07}
{3.99,4.15}
{4.35,4.82}
{4.22,4.95}
{4.01,4.91}
{4.12,5.48}
{4.10,5.20}
f2 (ppm)
f1 (ppm)

HMBC

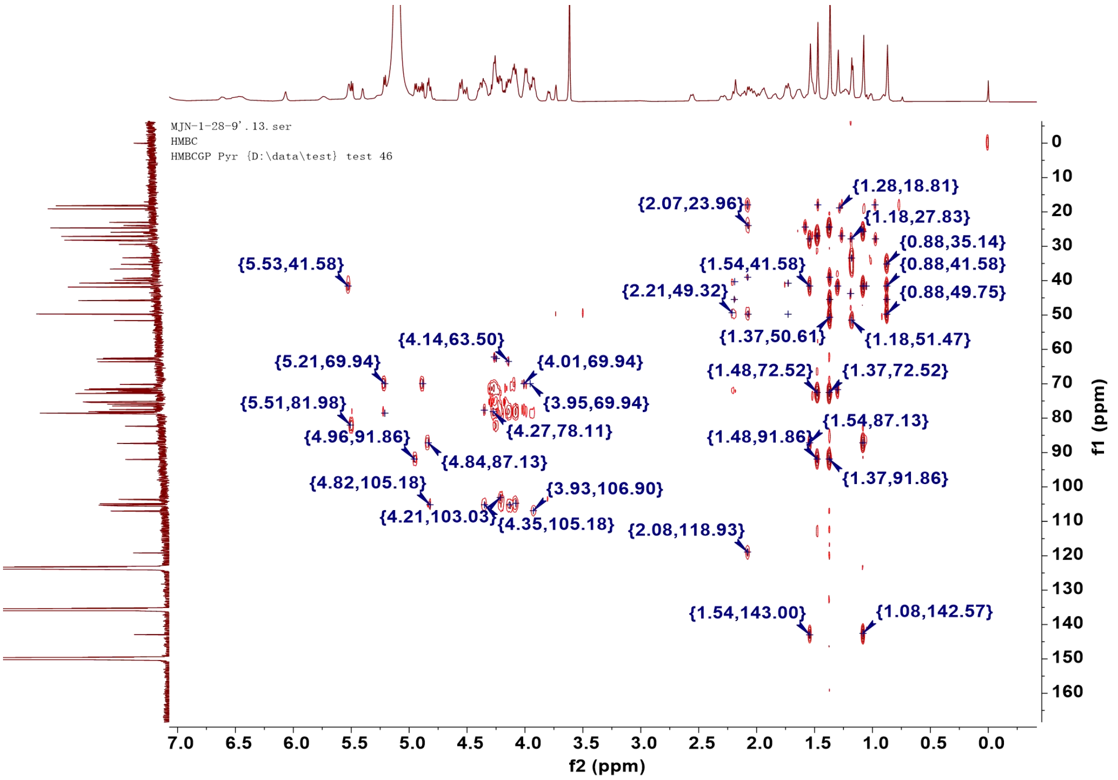
MJN-1-28-9'.13.ser
HMBC
HMBCGP Pyr {D:\data\test} test 46
{1.28,18.81}
{2.07,23.96}
{1.18,27.83}
{0.88,35.14}
{5.53,41.58}
{1.54,41.58}
{0.88,41.58}
{2.21,49.32}
{0.88,49.75}
{1.37,50.61}
{1.18,51.47}
{4.14,63.50}
{5.21,69.94}
{4.01,69.94}
{1.48,72.52}
{1.37,72.52}
{3.95,69.94}
{5.51,81.98}
{4.27,78.11}
{1.54,87.13}
{4.96,91.86}
{1.48,91.86}
{4.84,87.13}
{1.37,91.86}
{4.82,105.18}
{3.93,106.90}
{4.21,103.03}
{4.35,105.18}
{2.08,118.93}
{1.54,143.00}
{1.08,142.57}
f2 (ppm)
f1 (ppm)

NOESY

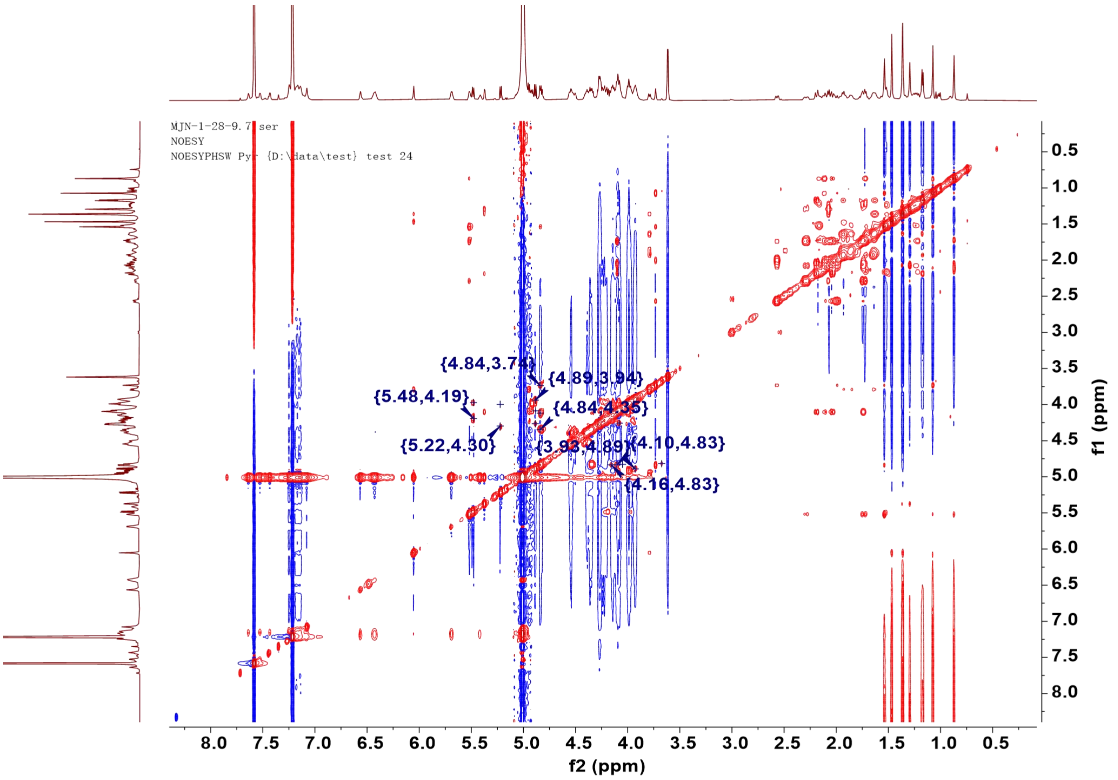

Compound **7**

^{1}H NMR

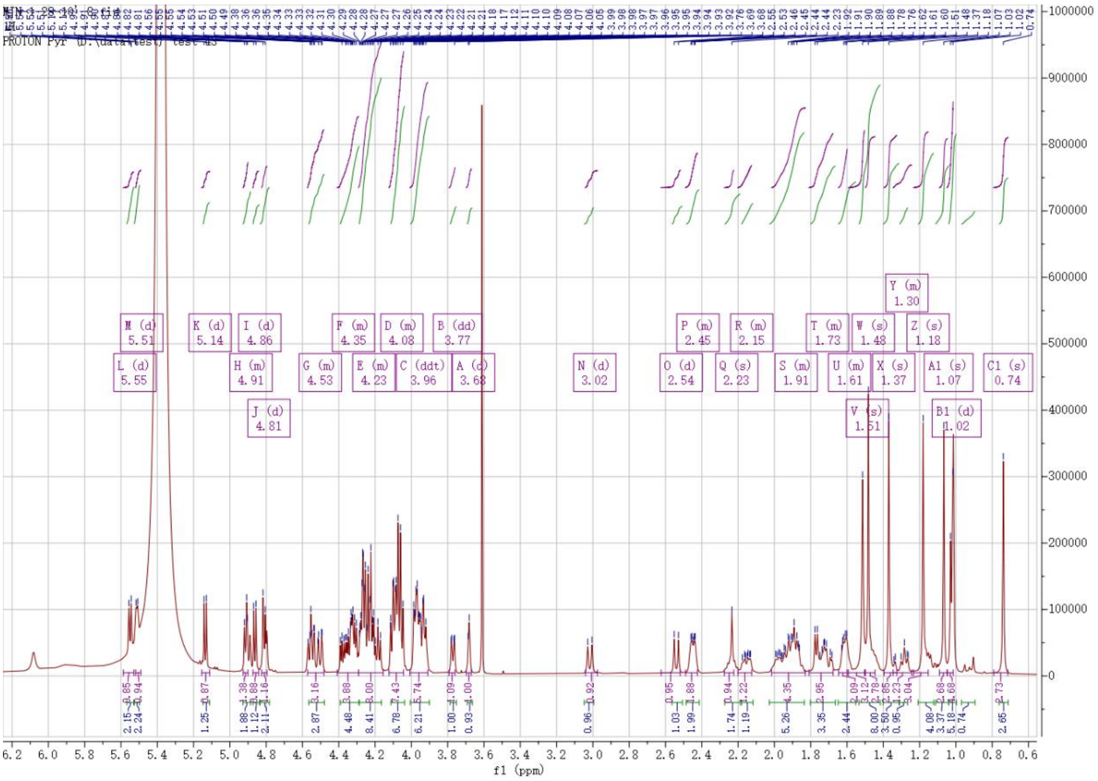

^{13}C NMR

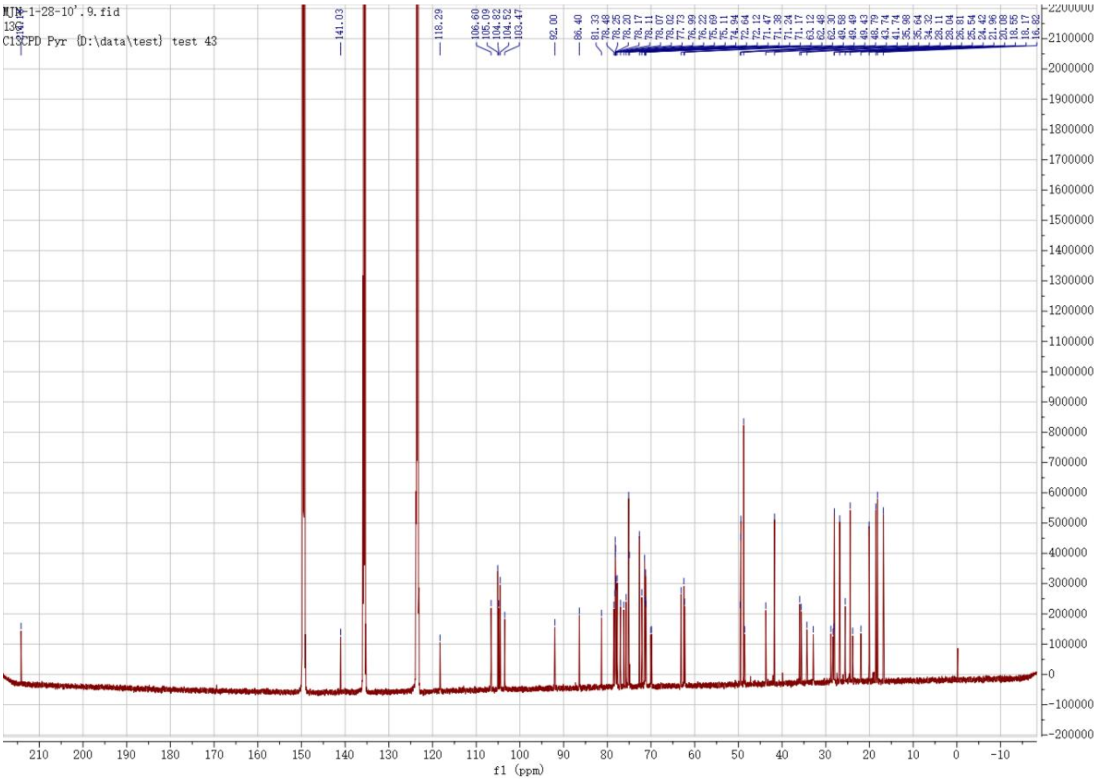

HSQC

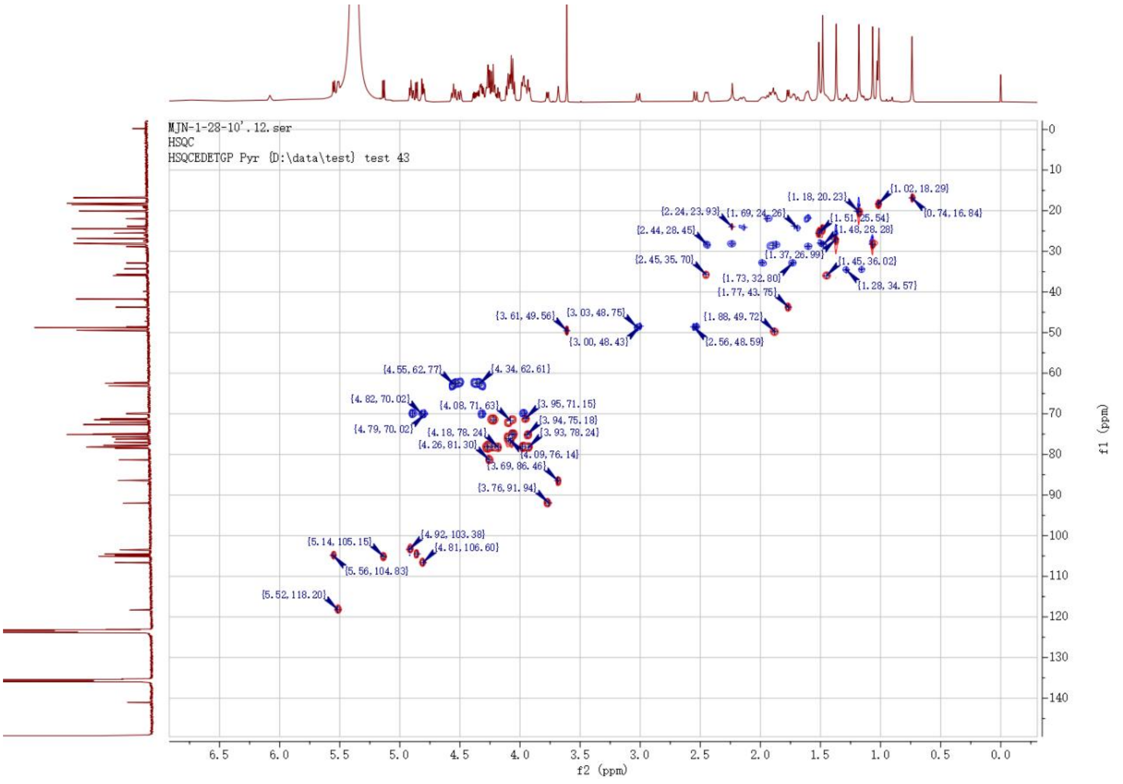

^{1}H-^{1}H COSY

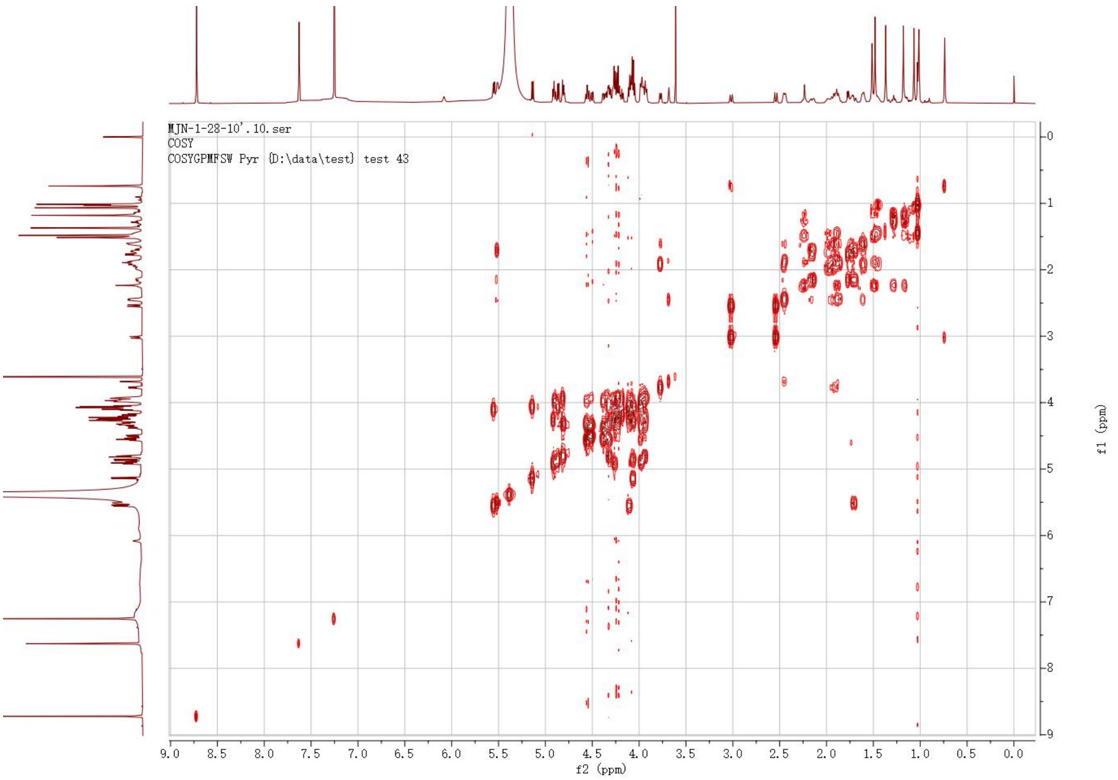
MJN-1-28-10'.10.ser
COSY
COSYGPMFSW Pyr {D:\data\test} test 43
f2 (ppm)
f1 (ppm)

HMBC

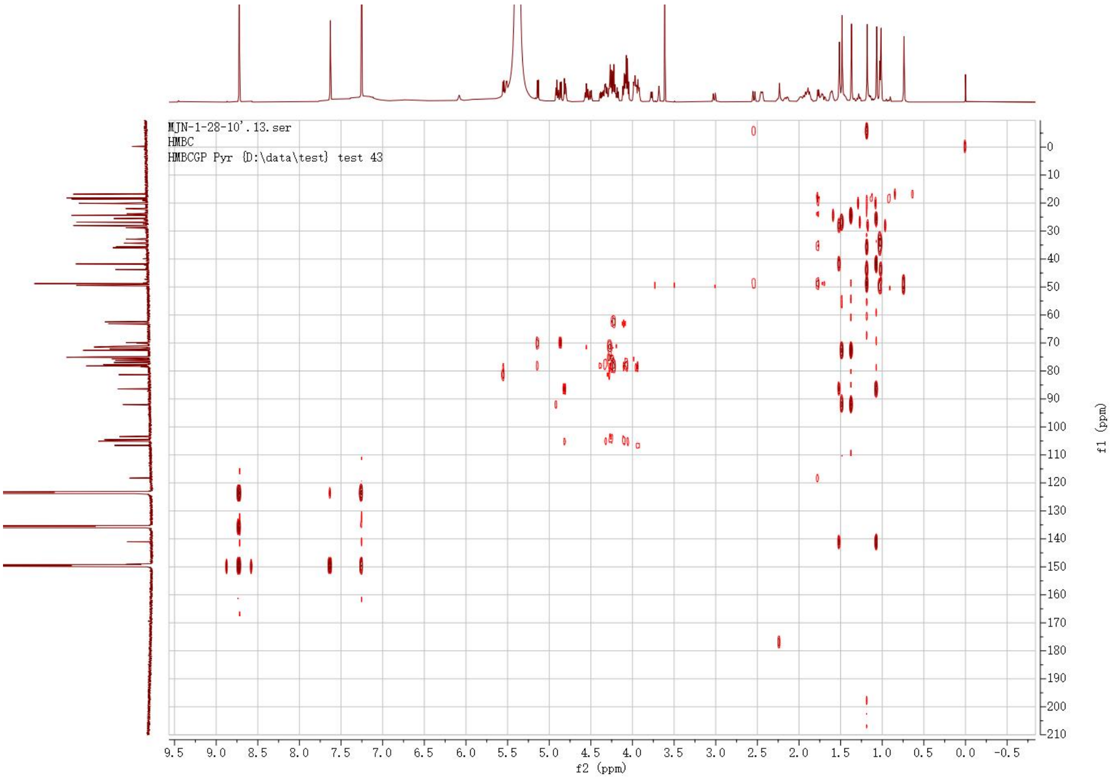
MJN-1-28-10'.13.ser
HMBC
HMBCGP Pyr {D:\data\test} test 43
f2 (ppm)
f1 (ppm)

ROESY

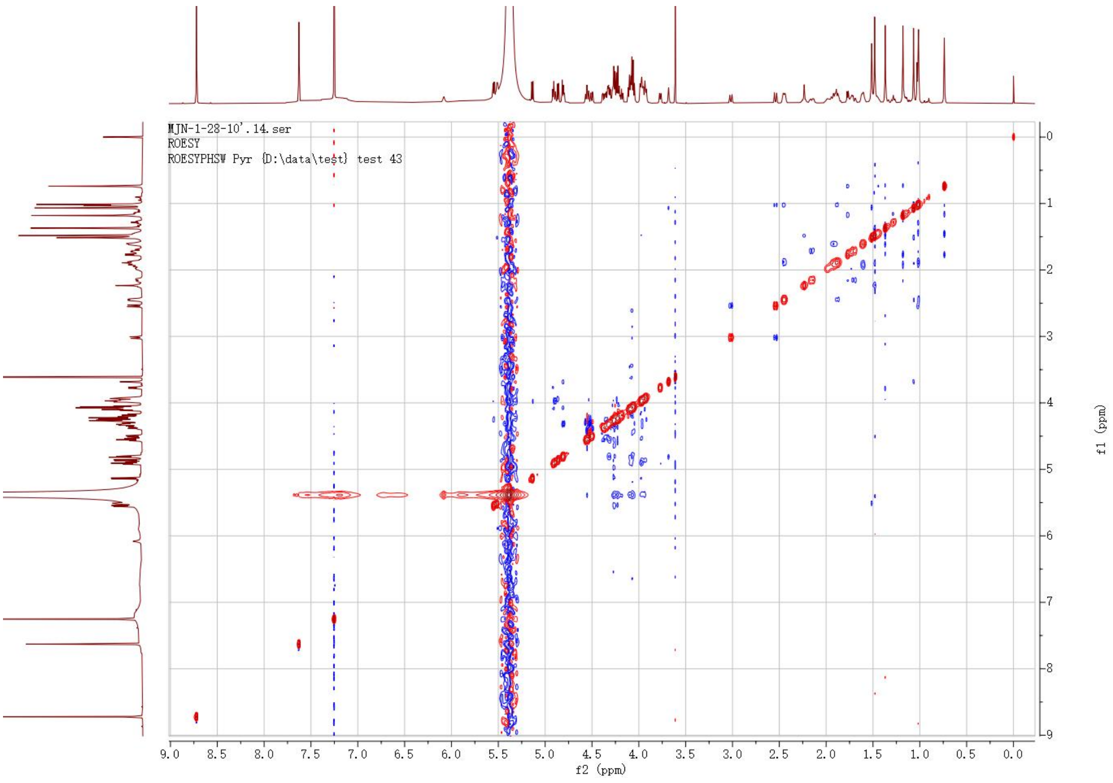
MJN-1-28-10'.14.ser
ROESY
ROESYPHSW Pyr {D:\data\test} test 43
f2 (ppm)
f1 (ppm)

HRESIMS $[M+Na]^+$

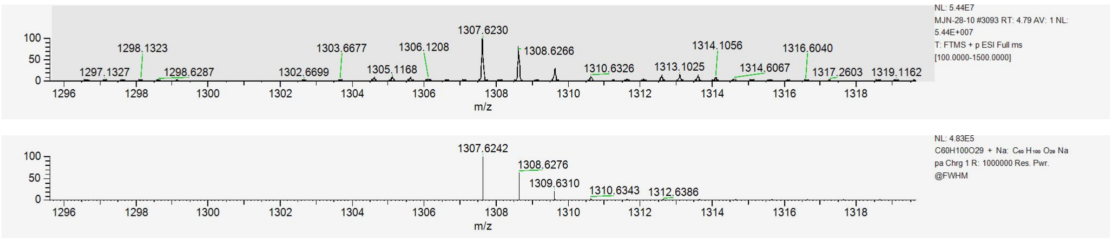
NL: 5.44E7
MJN-28-10 #3093 RT: 4.79 AV: 1 NL: 5.44E+007
T: FTMS + p ESI Full ms [100.0000-1500.0000]
1307.6230
1308.6266
NL: 4.83E5
C60H100O29 + Na
pa Chrg 1 R: 1000000 Res. Pwr. @FWHM
1307.6242
1308.6276
1309.6310
1310.6343
1312.6386
m/z

Compound **8**

^{1}H NMR

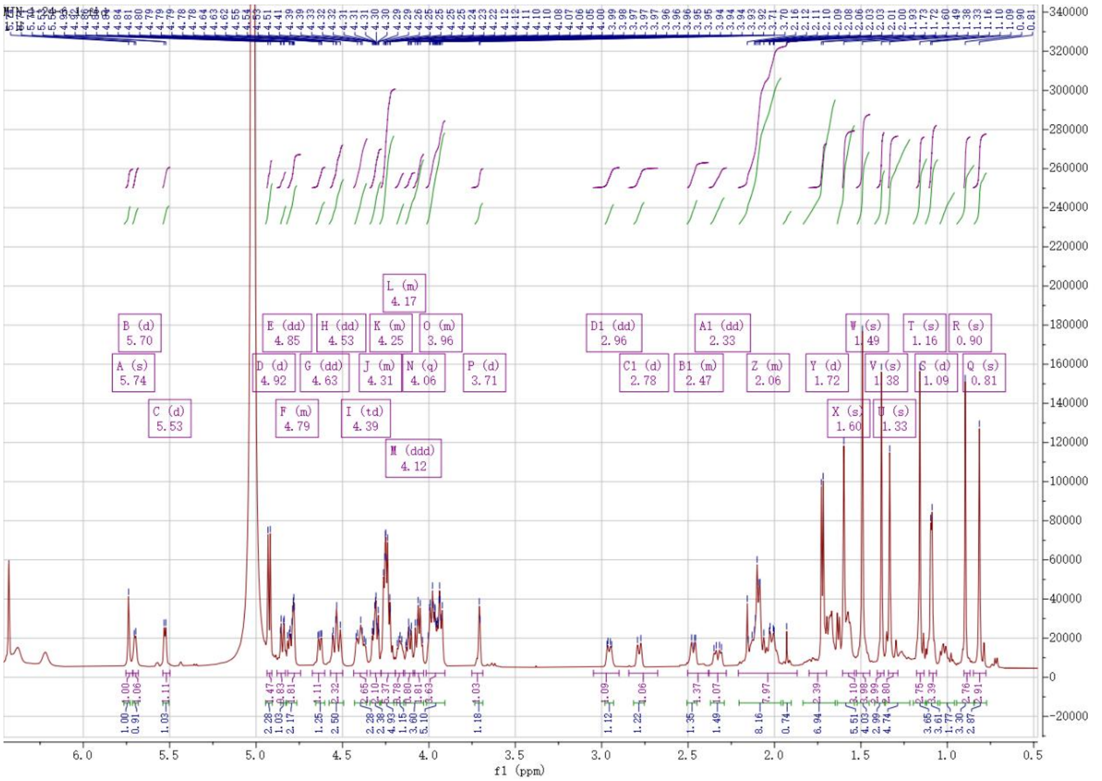

^{13}C NMR

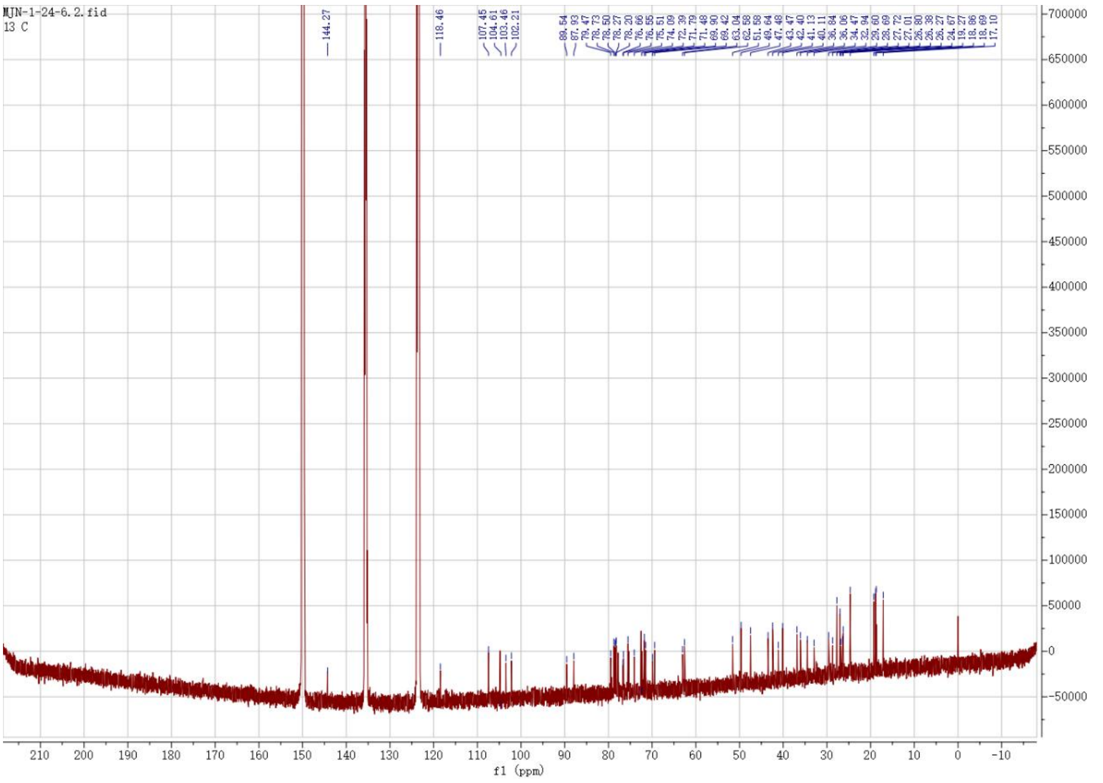

HSQC

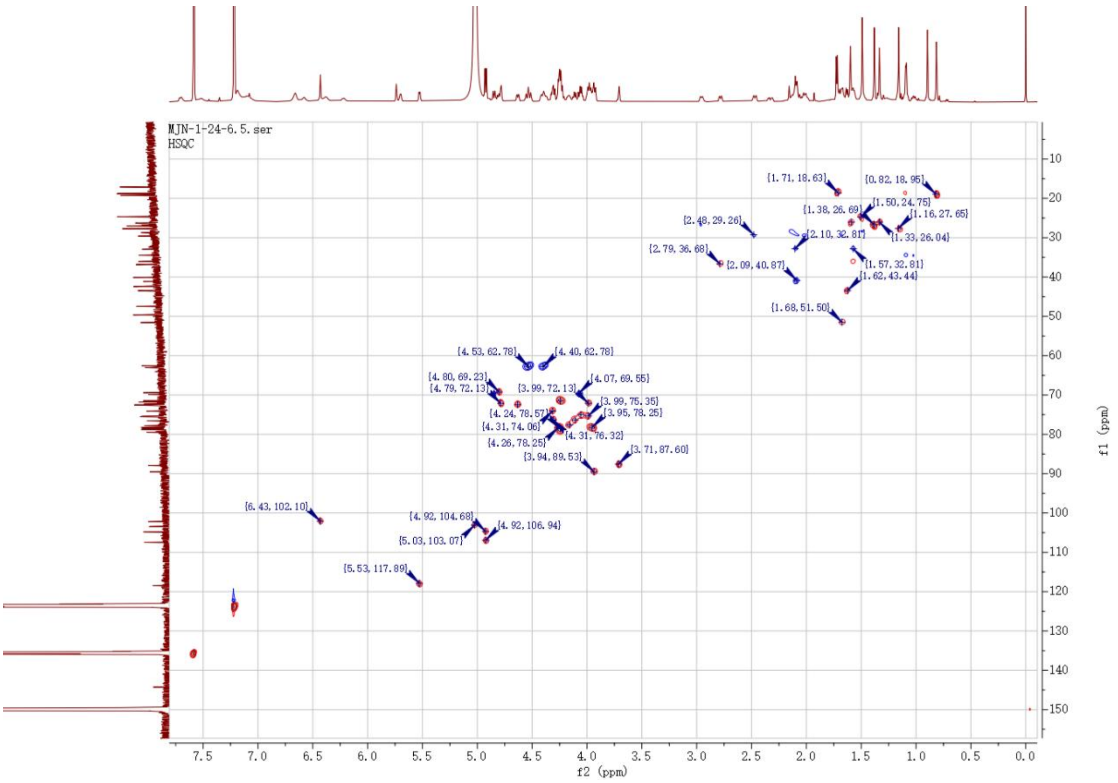

^1H-^1H COSY

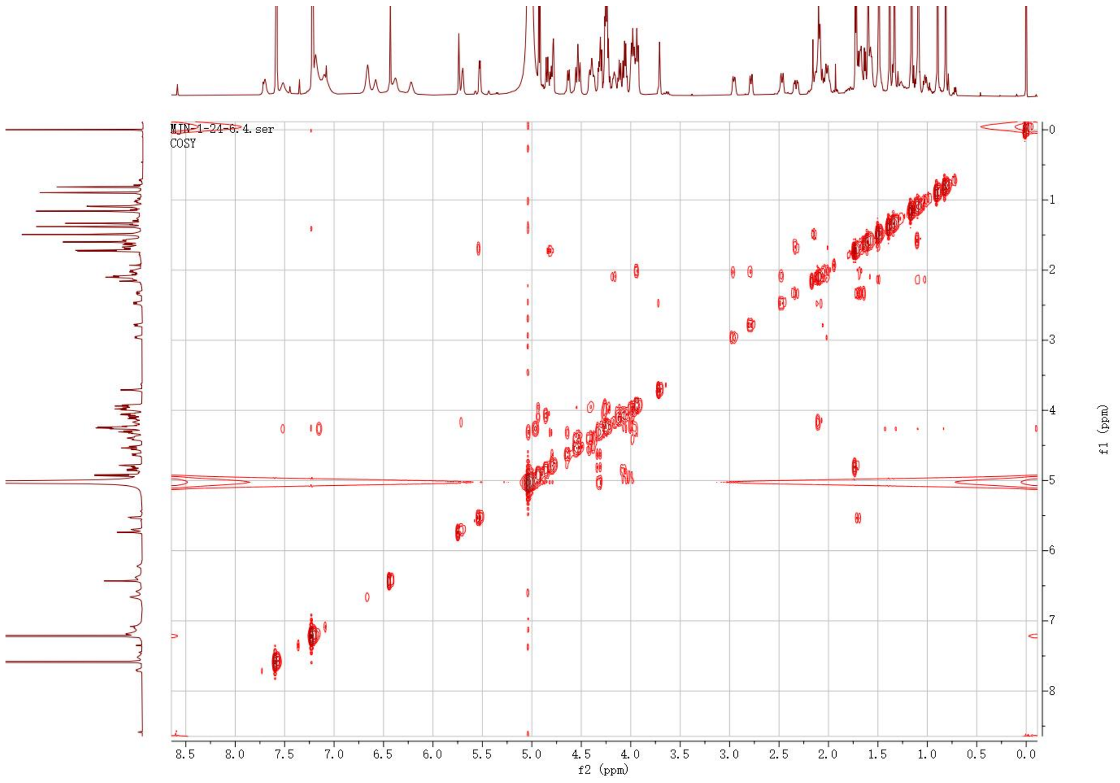
MJN-1-24-6.4.ser
COSY
f2 (ppm)
f1 (ppm)

HMBC

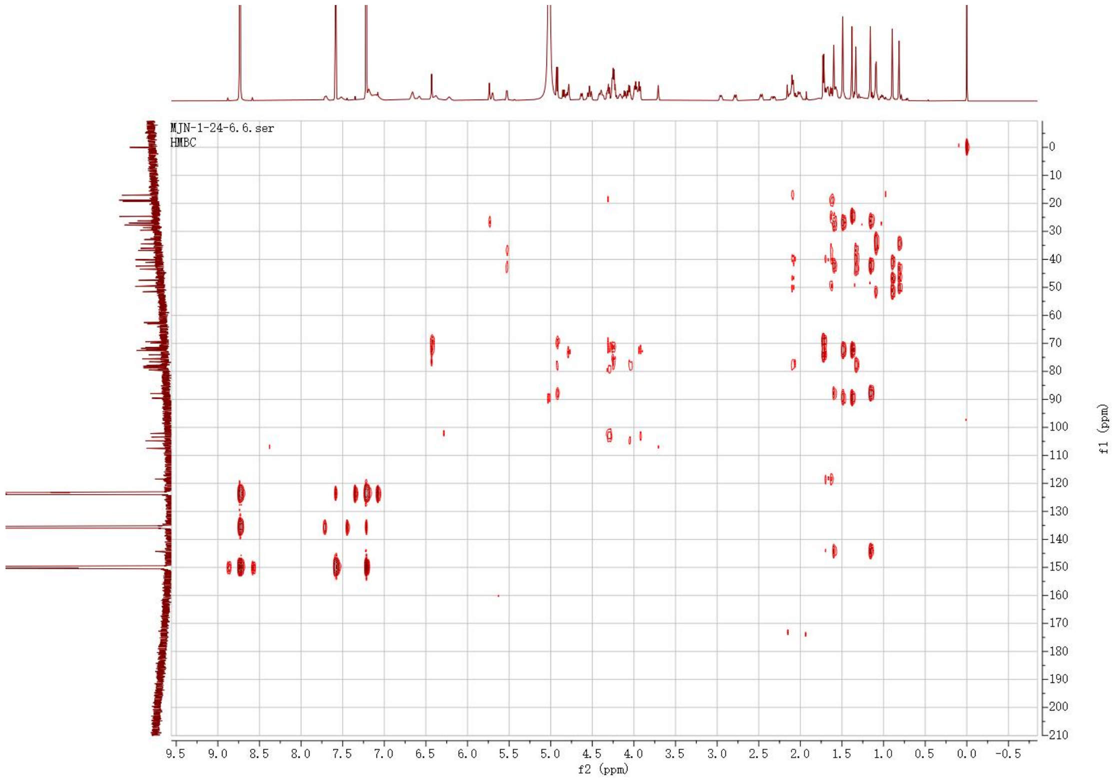
MJN-1-24-6.6.ser
HMBC
f2 (ppm)
f1 (ppm)

NOESY

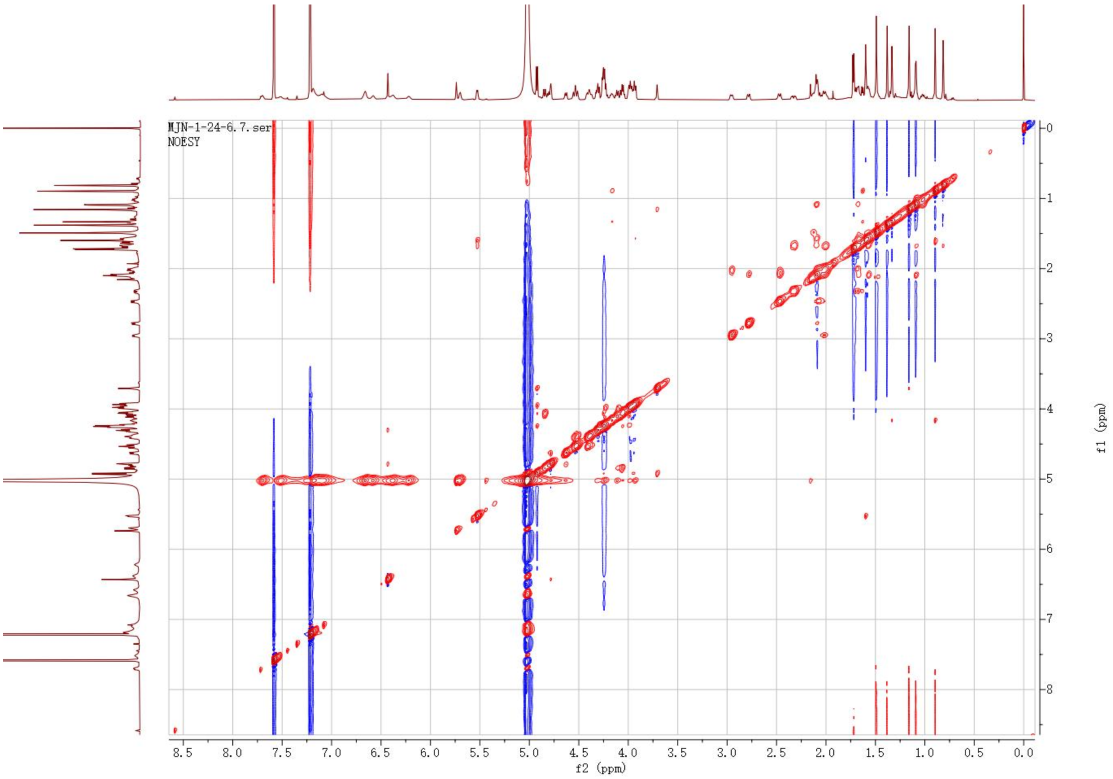

HRESIMS

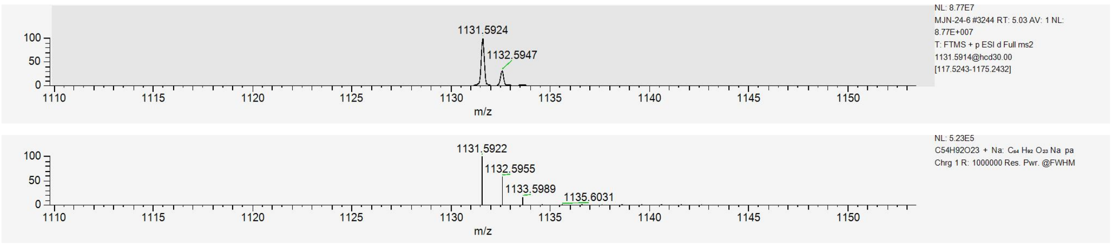

Compound **9**

^{1}H NMR

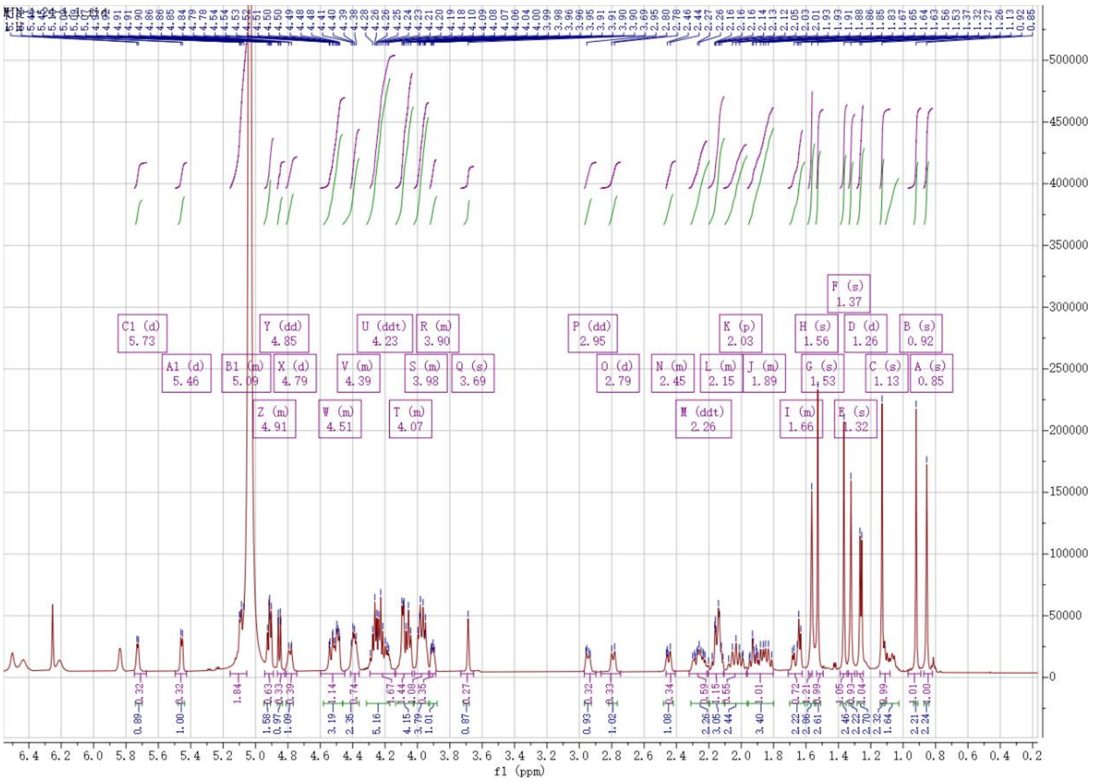

^{13}C NMR

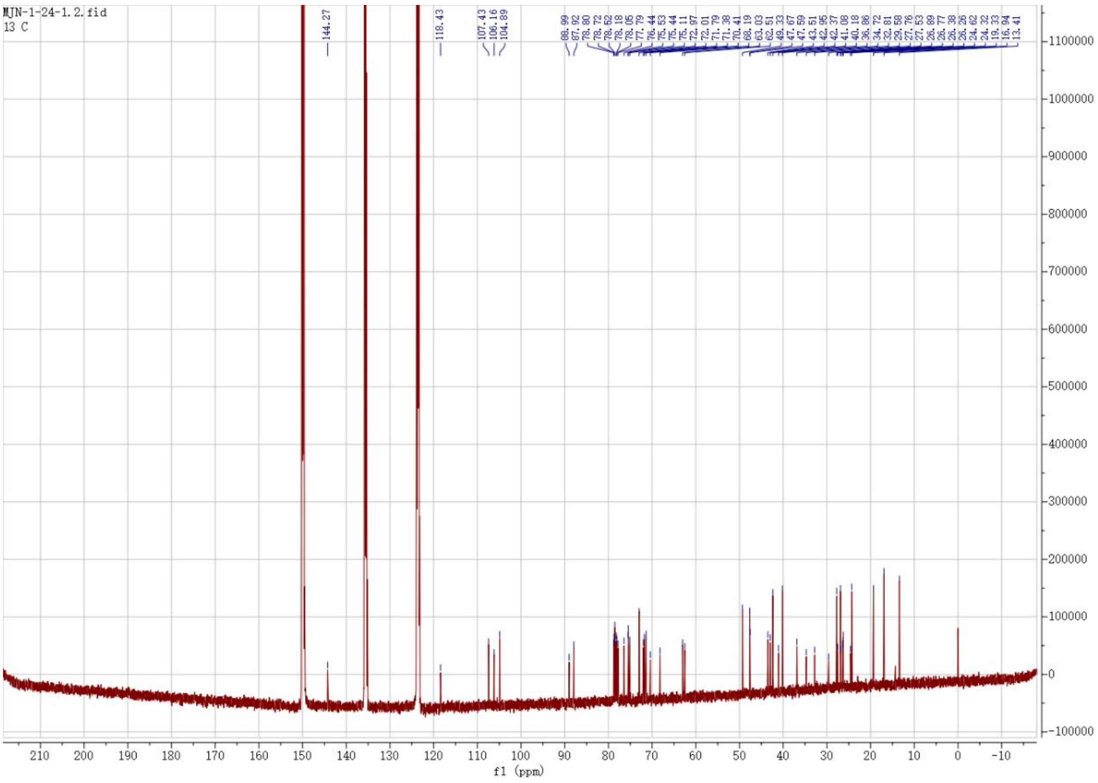

HSQC

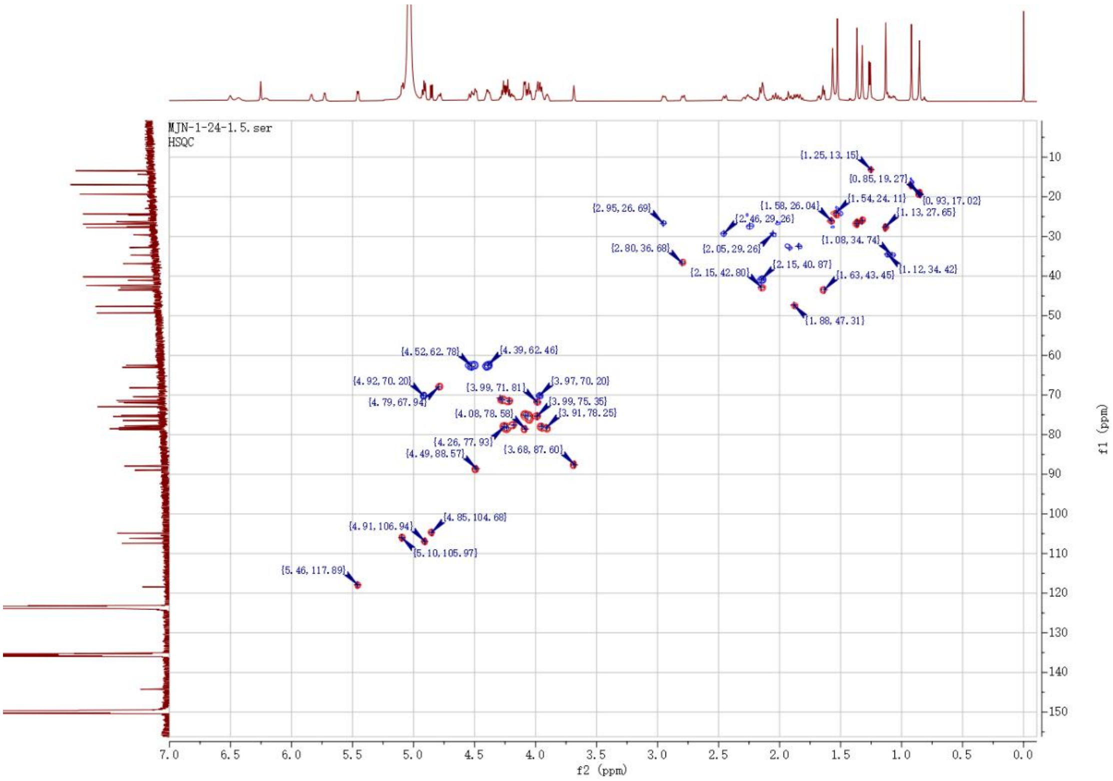

^1H-^1H COSY

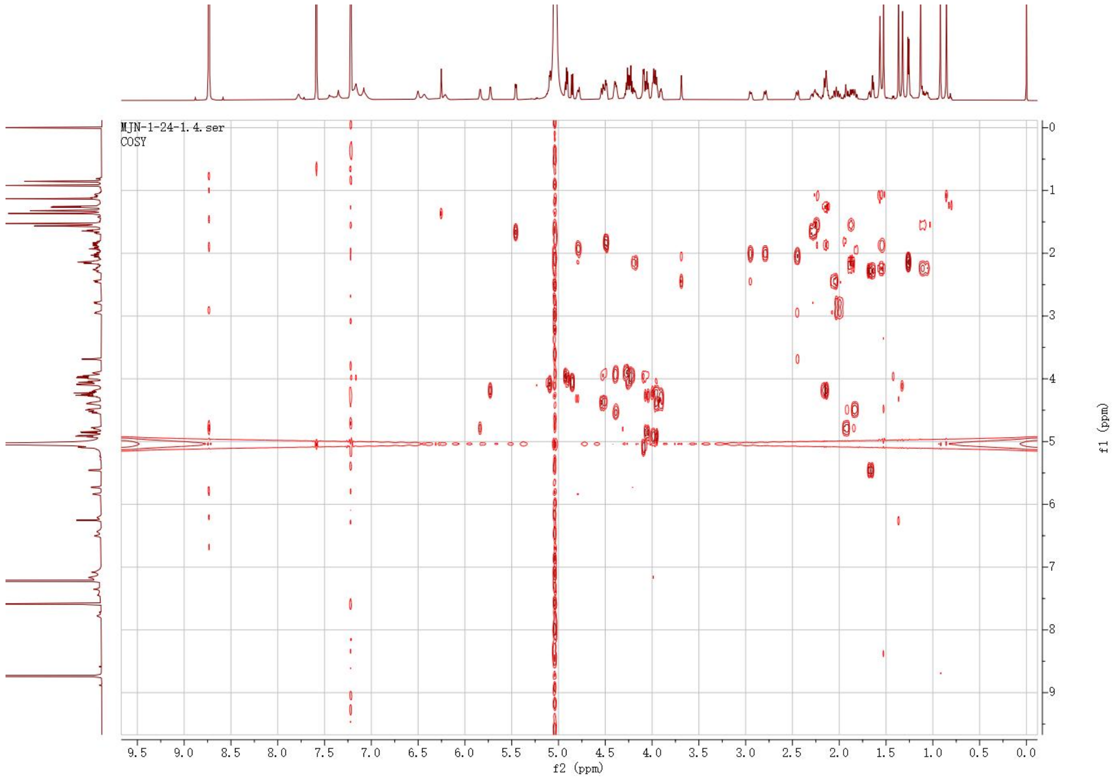

HMBC

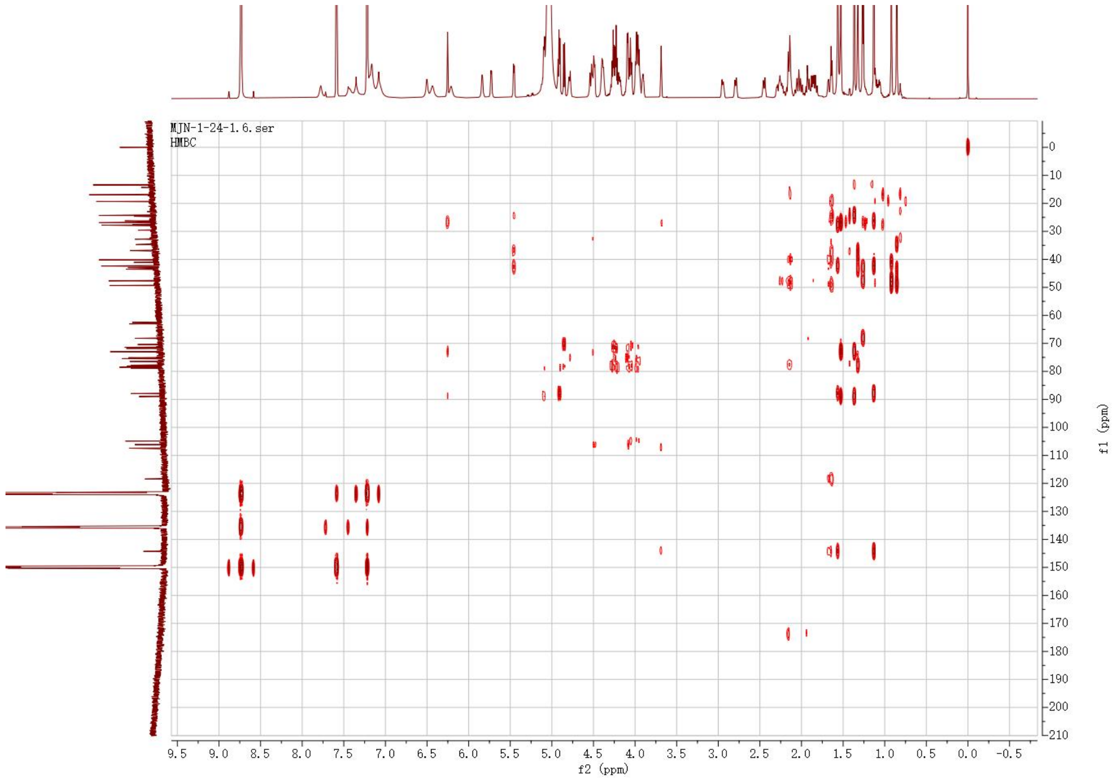

NOESY

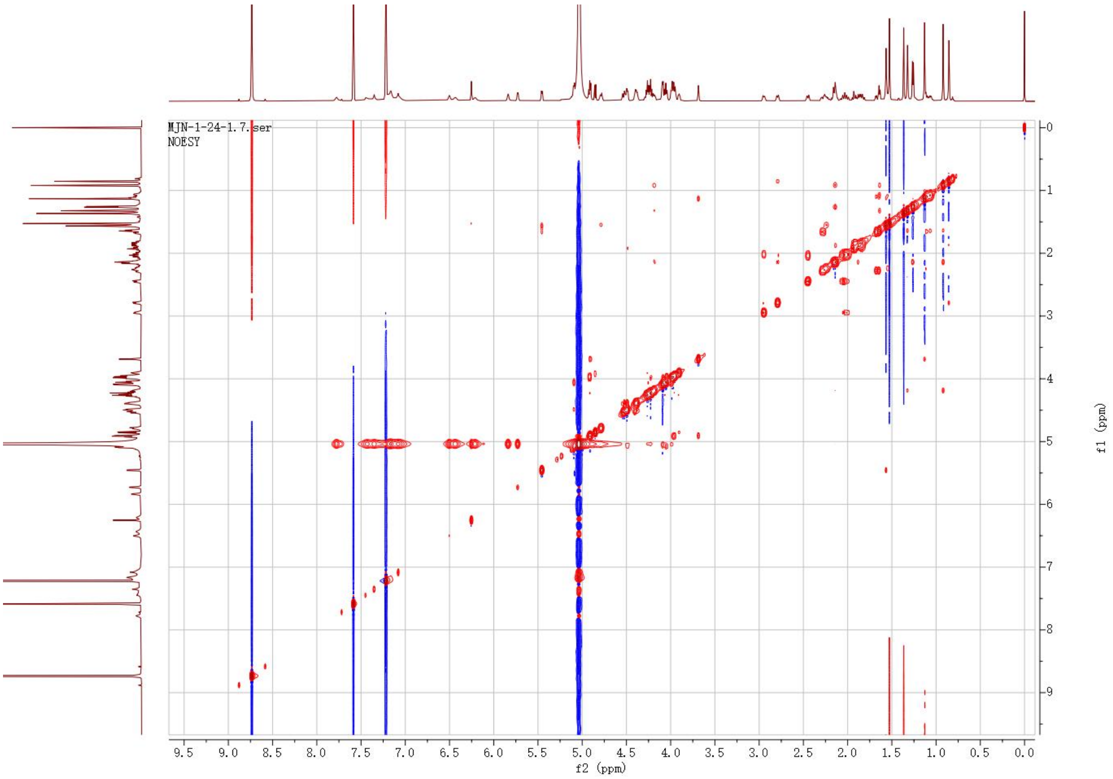

HRESIMS [M-H]$^-$

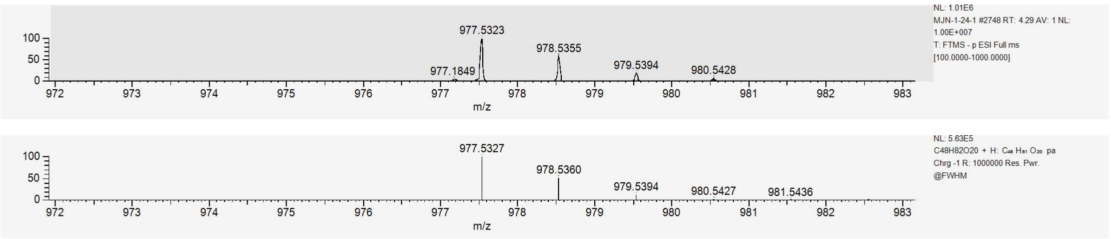

Compound **10**

^{1}H NMR

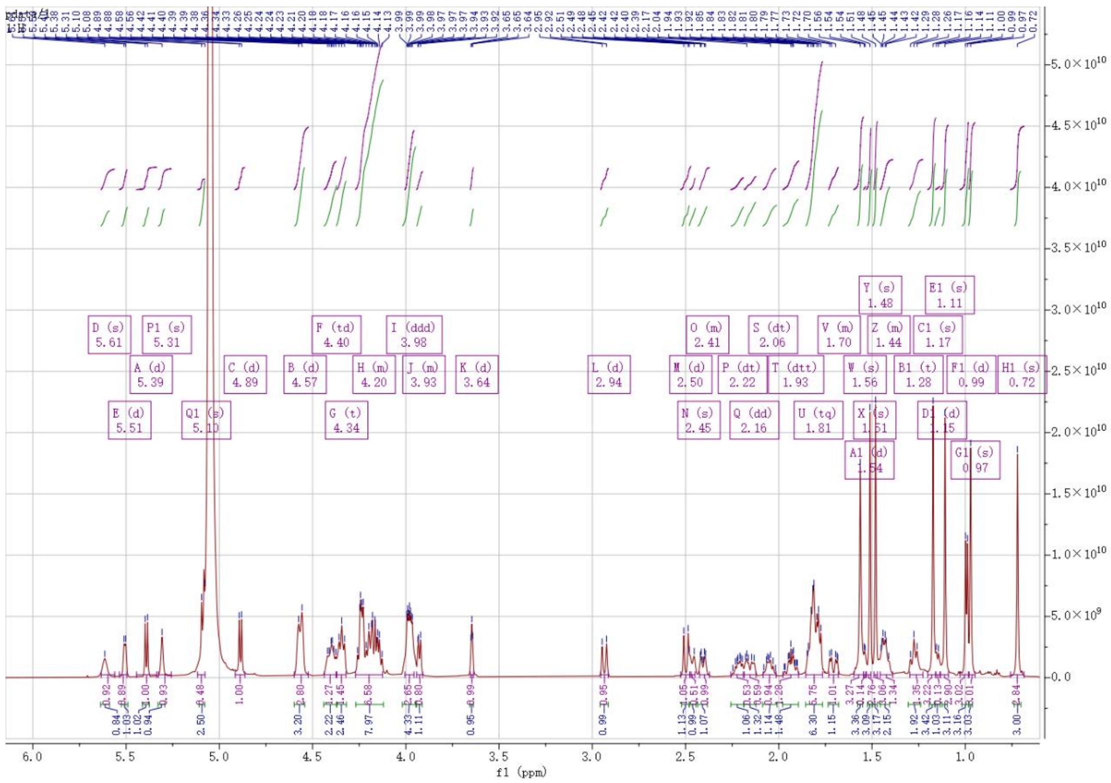

^{13}C NMR

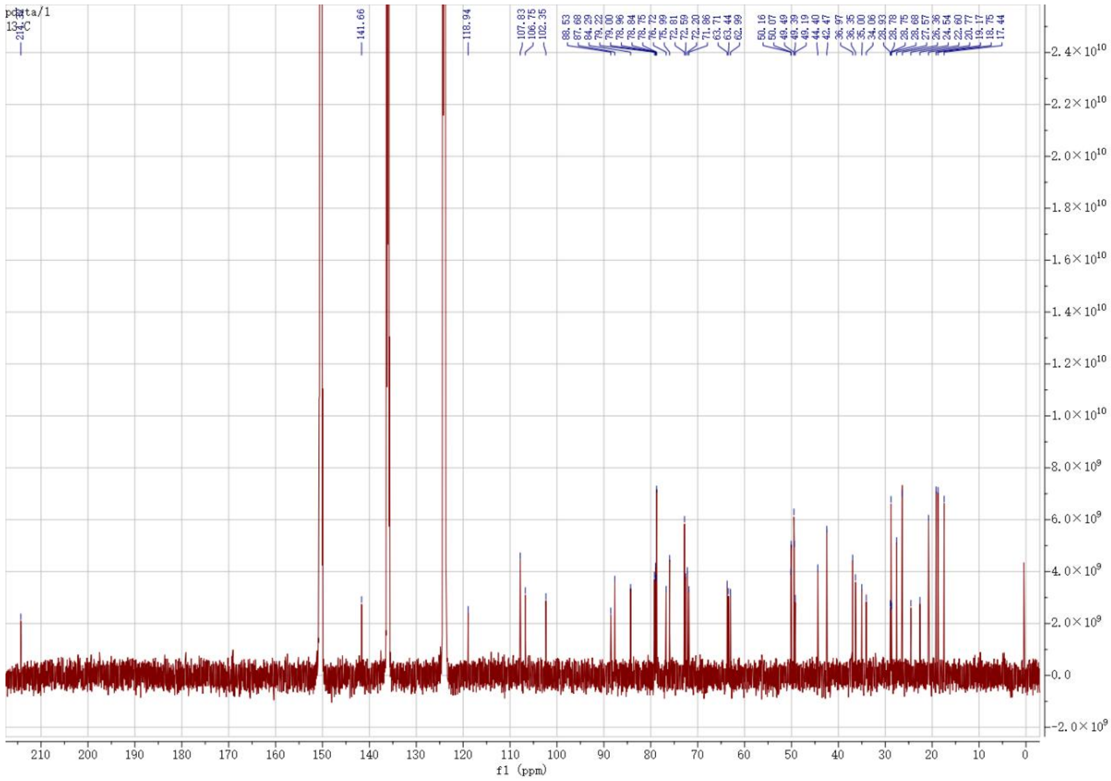
pdata/1
13C
141.66
118.94
107.83
106.75
102.35
f1 (ppm)

HSQC

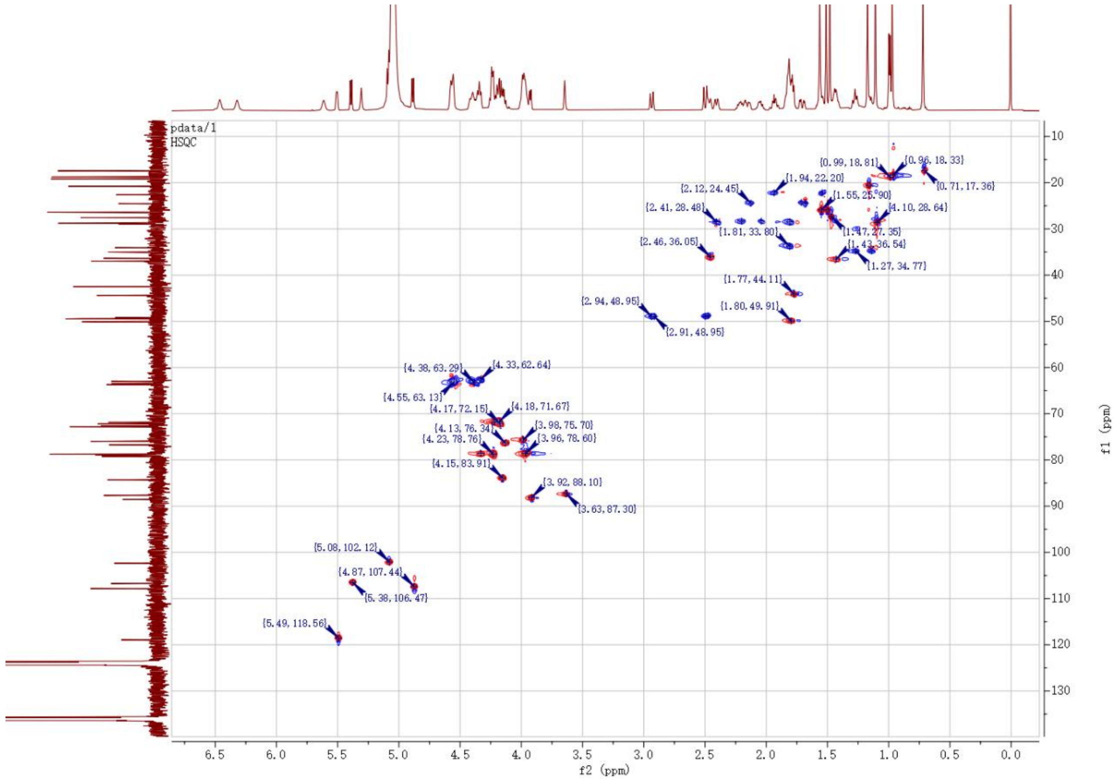
pdata/1
HSQC
[0.99, 18.81]
[0.96, 18.33]
[0.71, 17.36]
[1.94, 22.20]
[2.12, 24.45]
[1.55, 25.90]
[2.41, 28.48]
[4.10, 28.64]
[1.81, 33.80]
[1.47, 27.35]
[2.46, 36.05]
[1.43, 36.54]
[1.27, 34.77]
[1.77, 44.11]
[2.94, 48.95]
[1.80, 49.91]
[2.91, 48.95]
[4.38, 63.29]
[4.33, 62.64]
[4.55, 63.13]
[4.17, 72.15]
[4.18, 71.67]
[4.13, 76.34]
[3.98, 75.70]
[4.23, 78.76]
[3.96, 78.60]
[4.15, 83.91]
[3.92, 88.10]
[3.63, 87.30]
[5.08, 102.12]
[4.87, 107.44]
[5.38, 106.47]
[5.49, 118.56]
f2 (ppm)
f1 (ppm)

^{1}H-^{1}H COSY

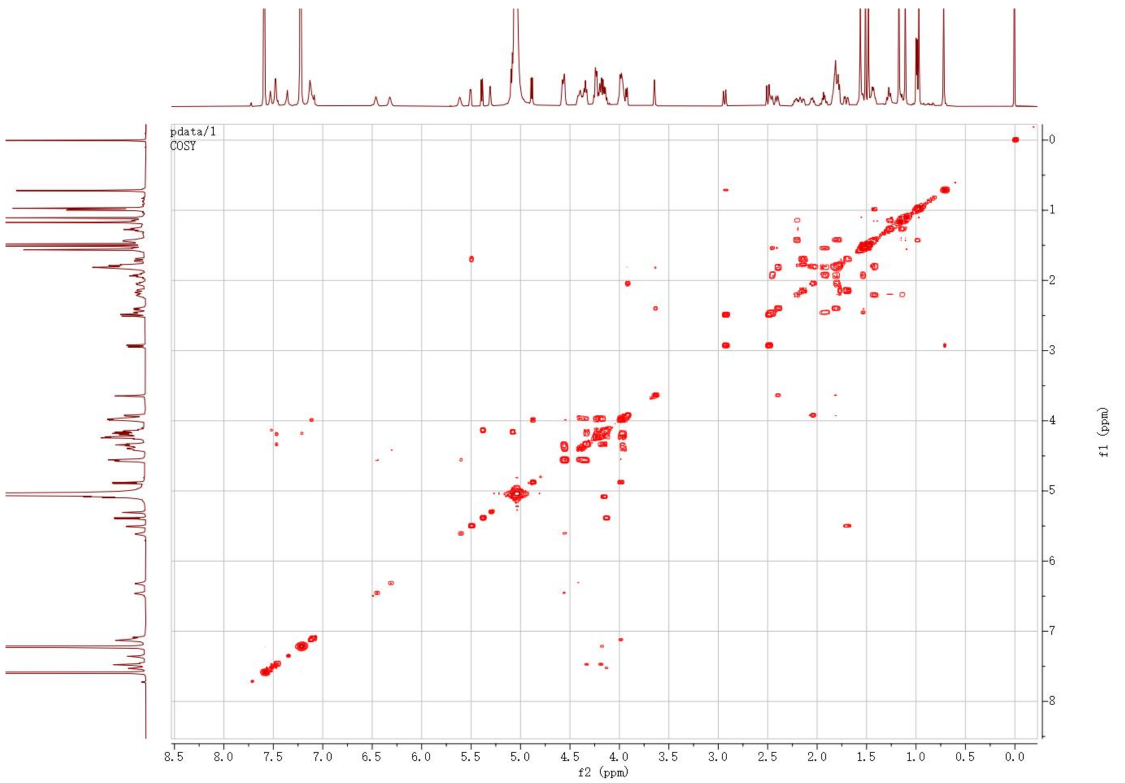

HMBC

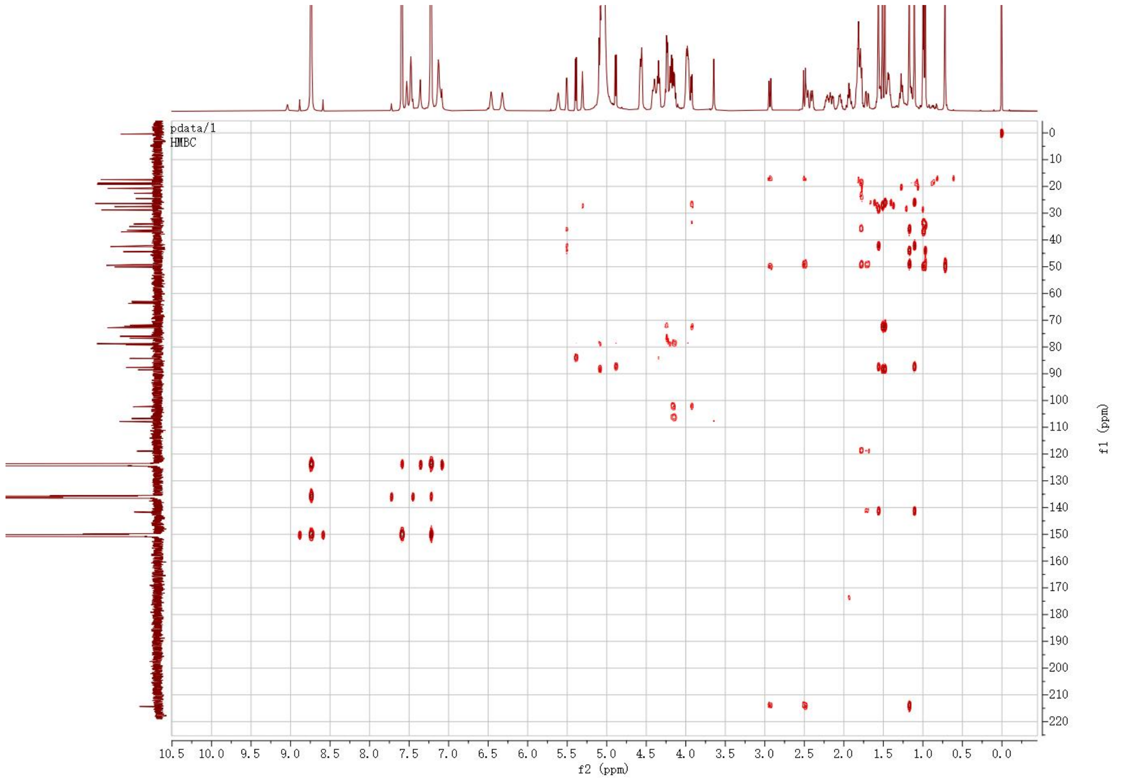

ROESY

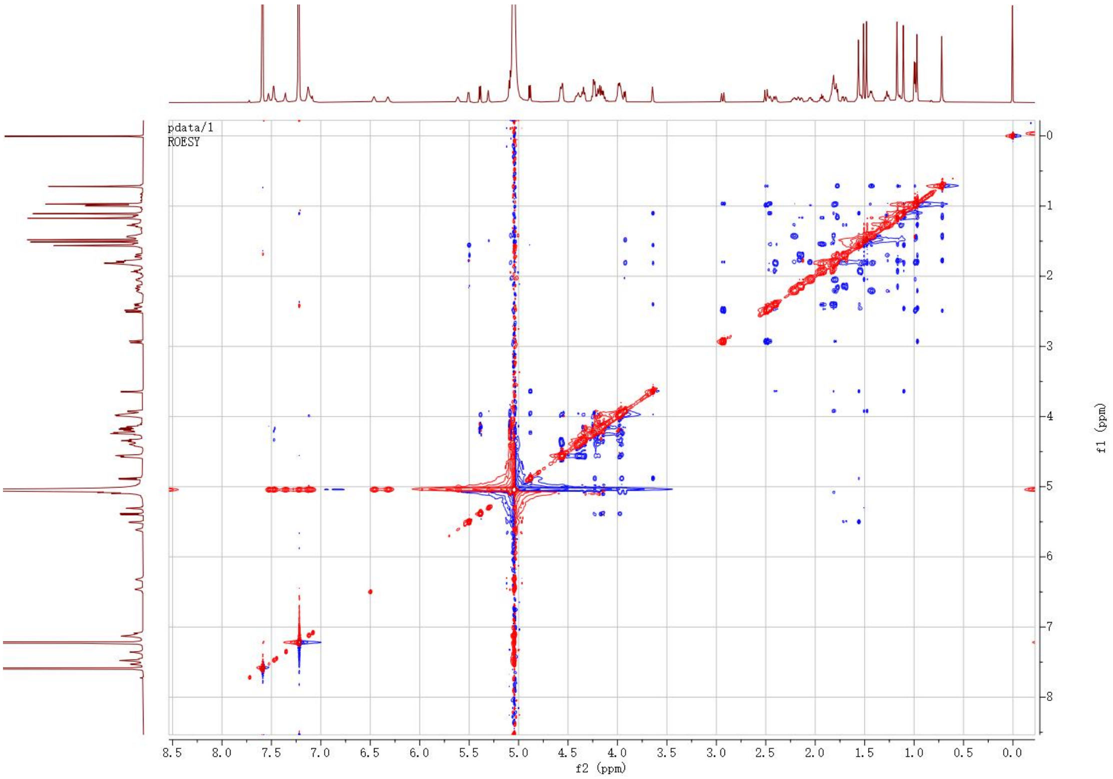

HRESIMS [M+Na]$^+$

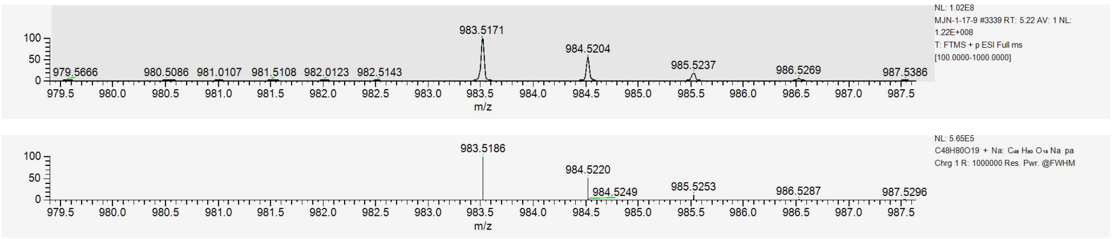

Compound **11**

^{1}H NMR

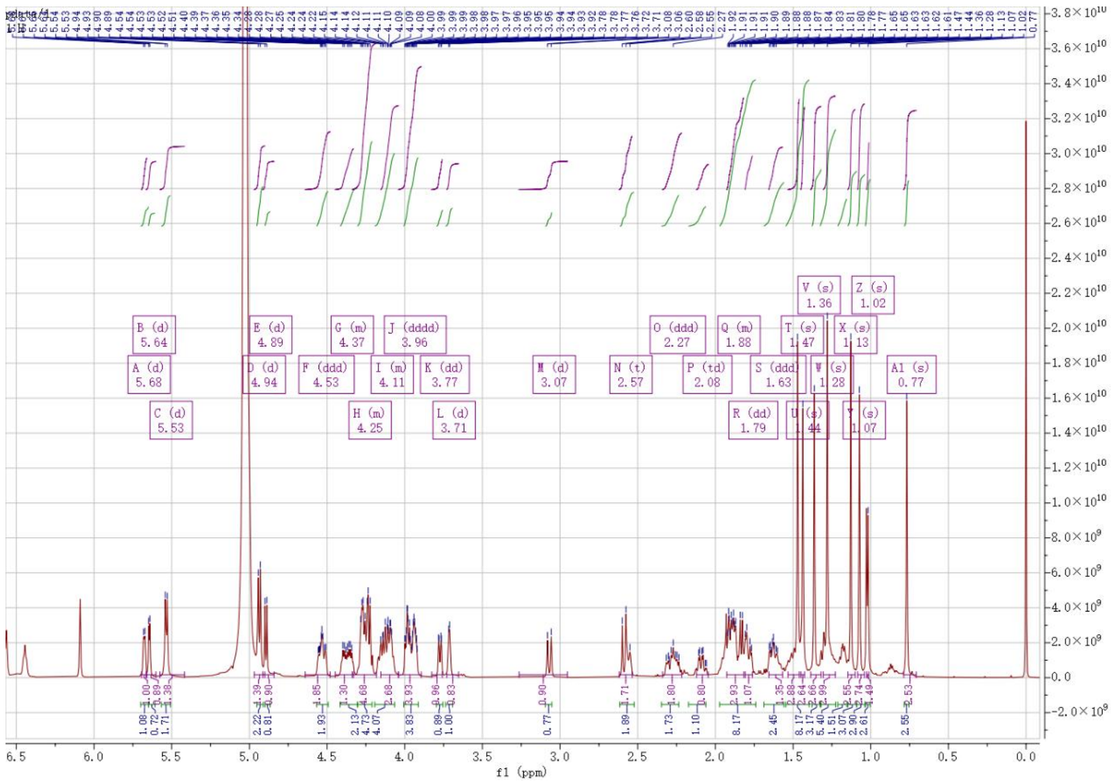

^{13}C NMR

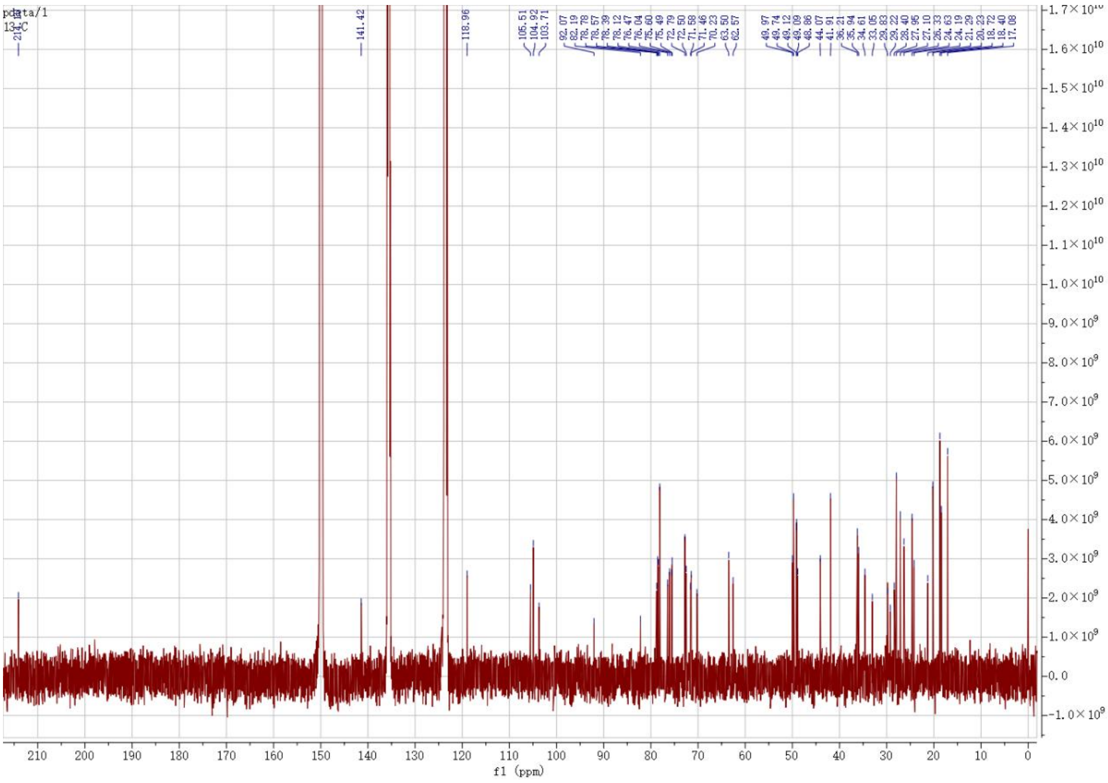

HSQC

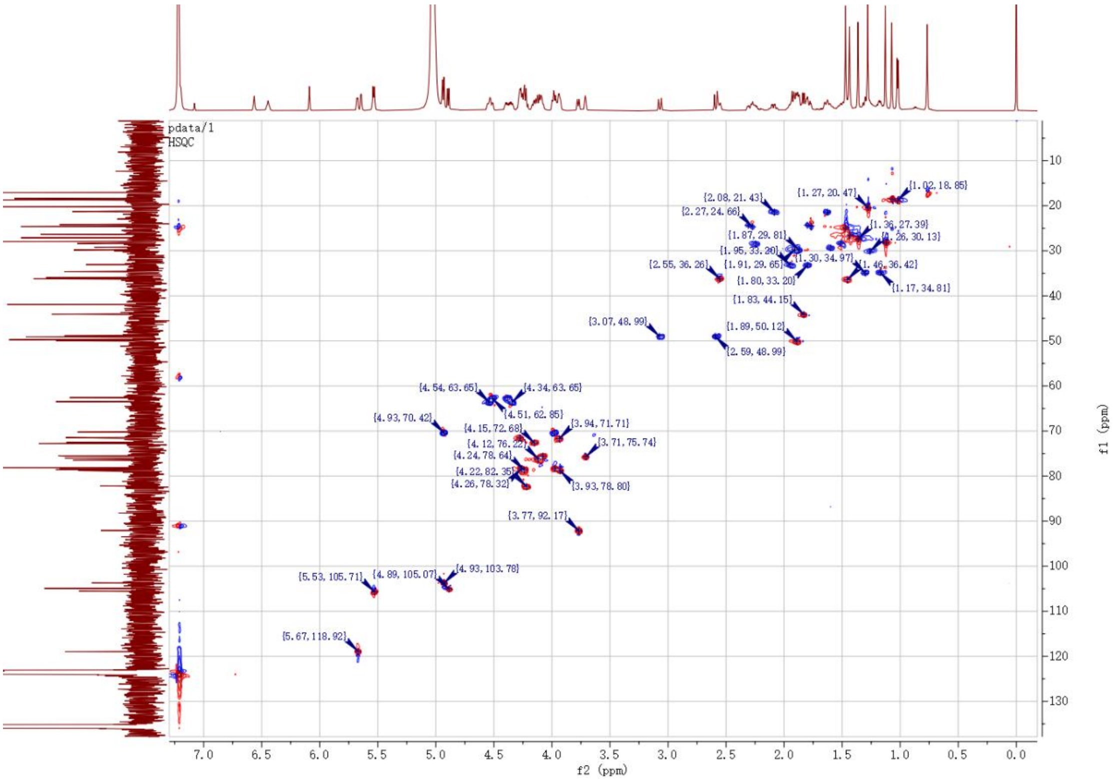

^{1}H-^{1}H COSY

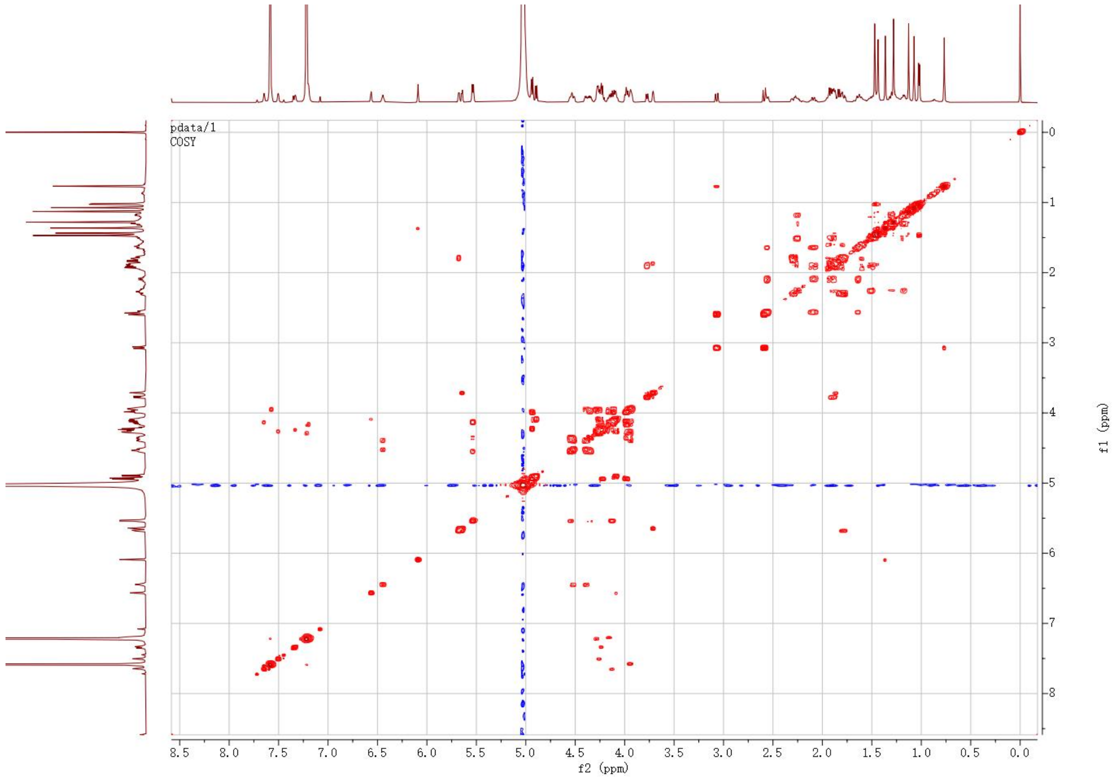

HMBC

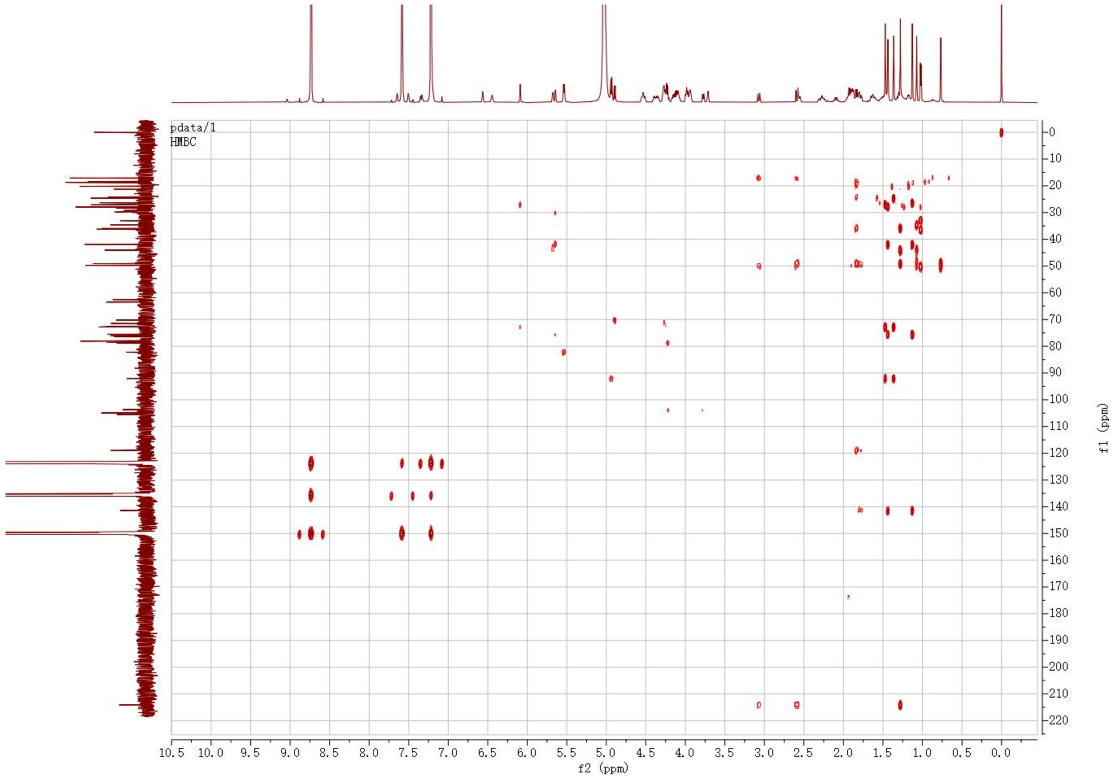

ROESY

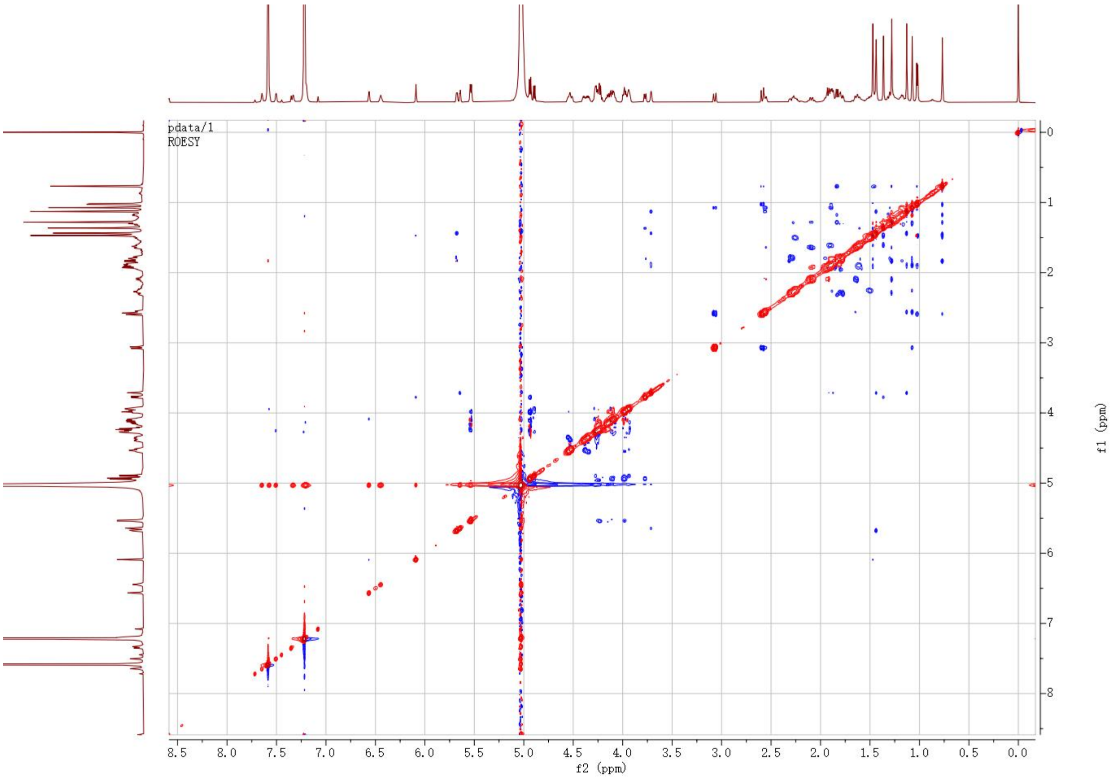

HRESIMS [M+Na]$^{+}$

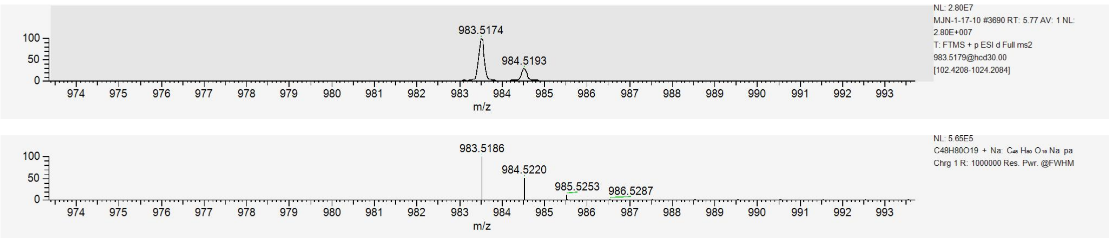

Compound **12**

^{1}H NMR

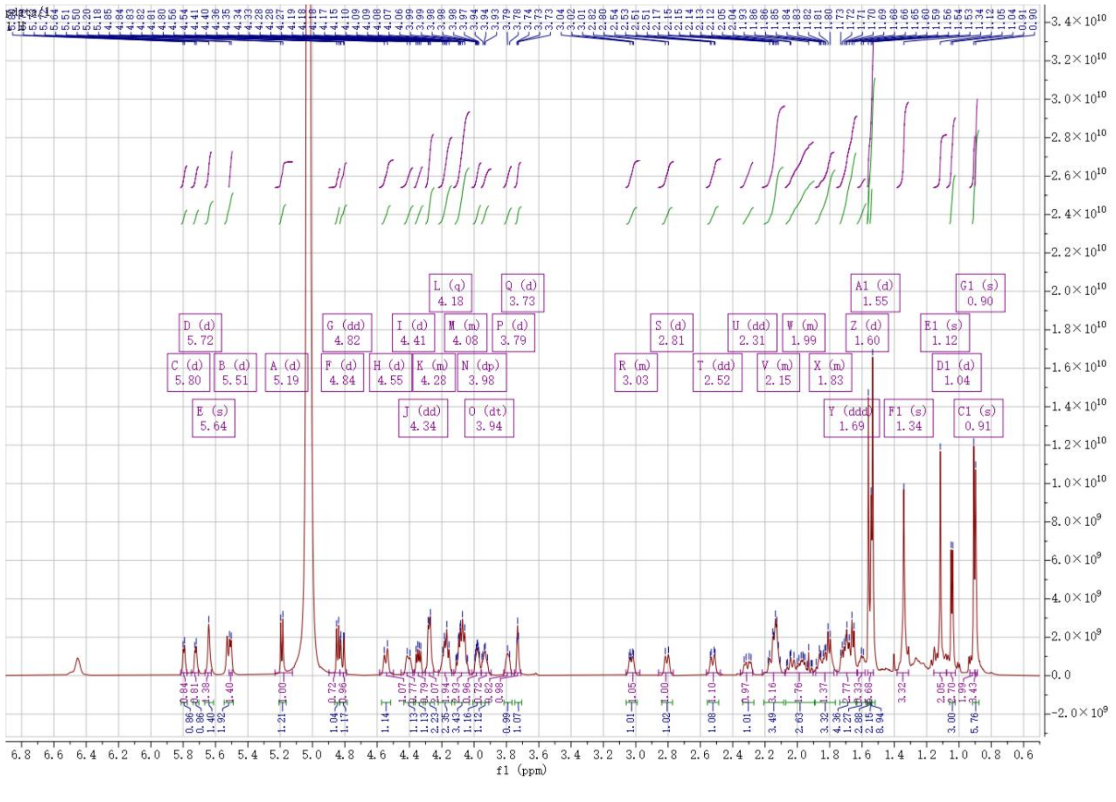

^{13}C NMR

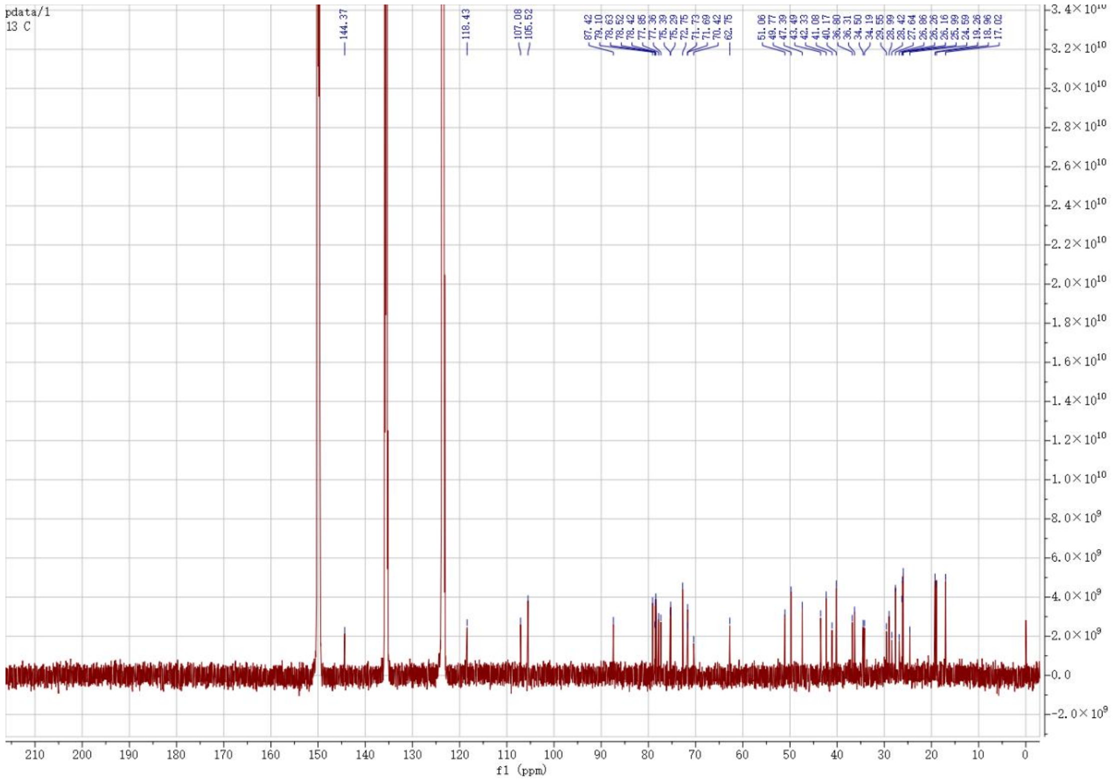

HSQC

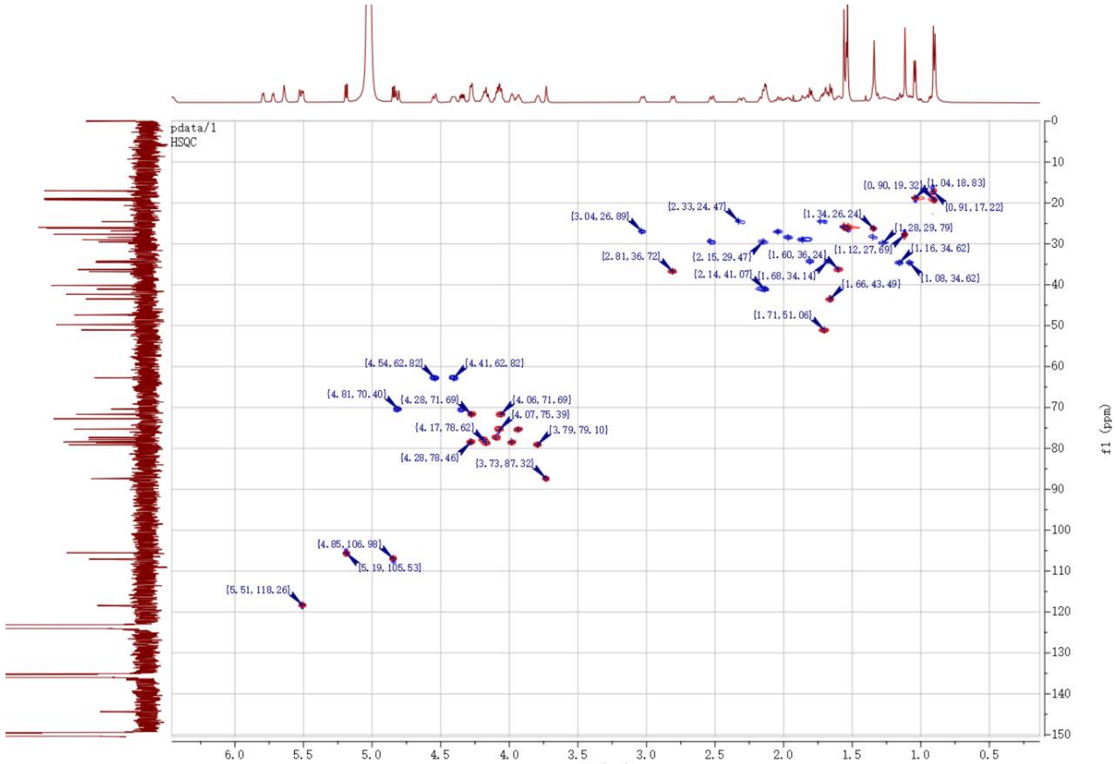

^{1}H-^{1}H COSY

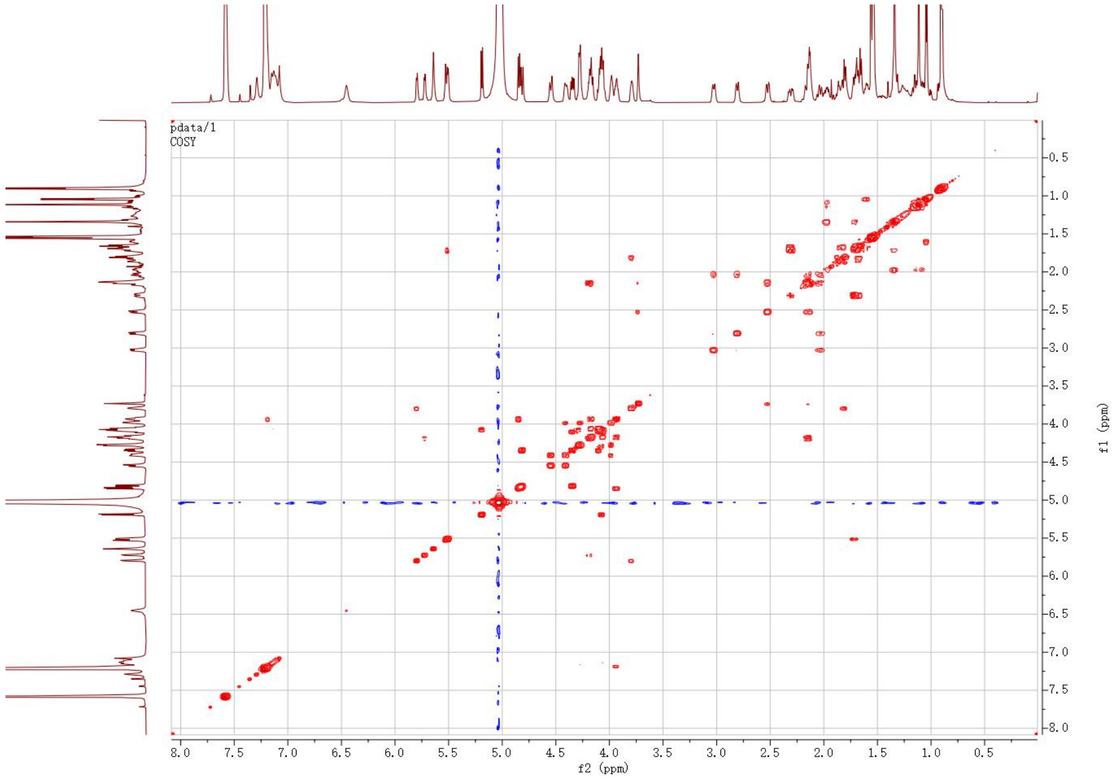

HMBC

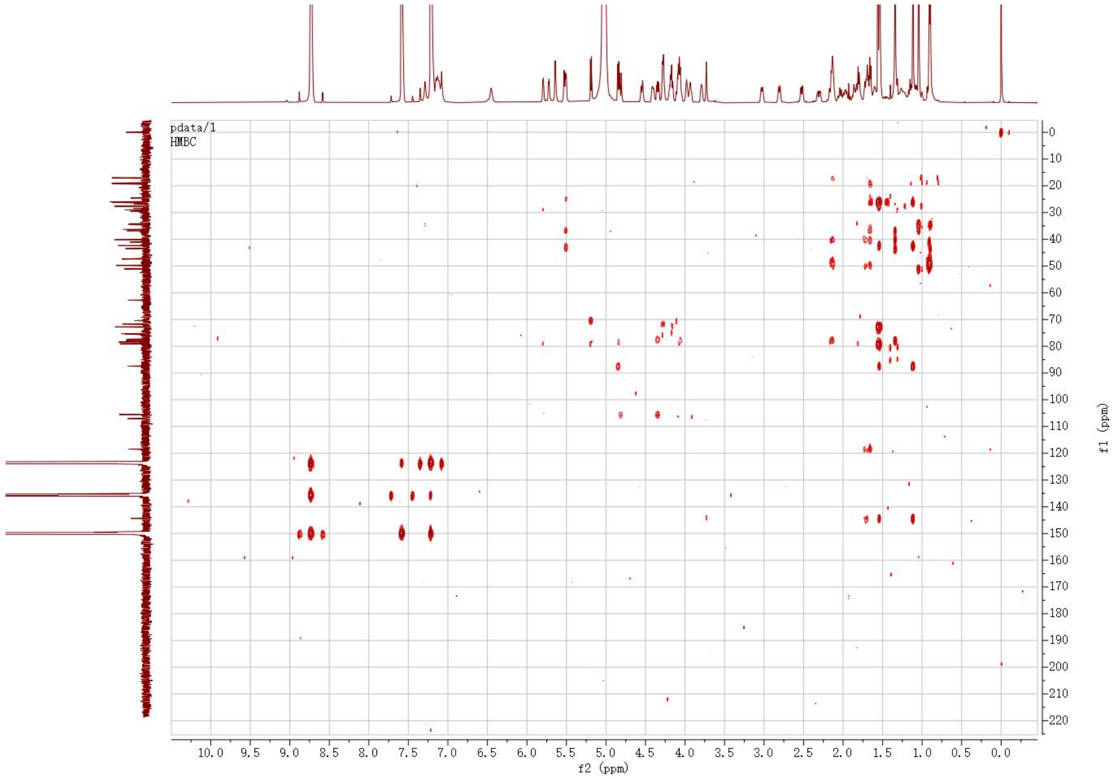

ROESY

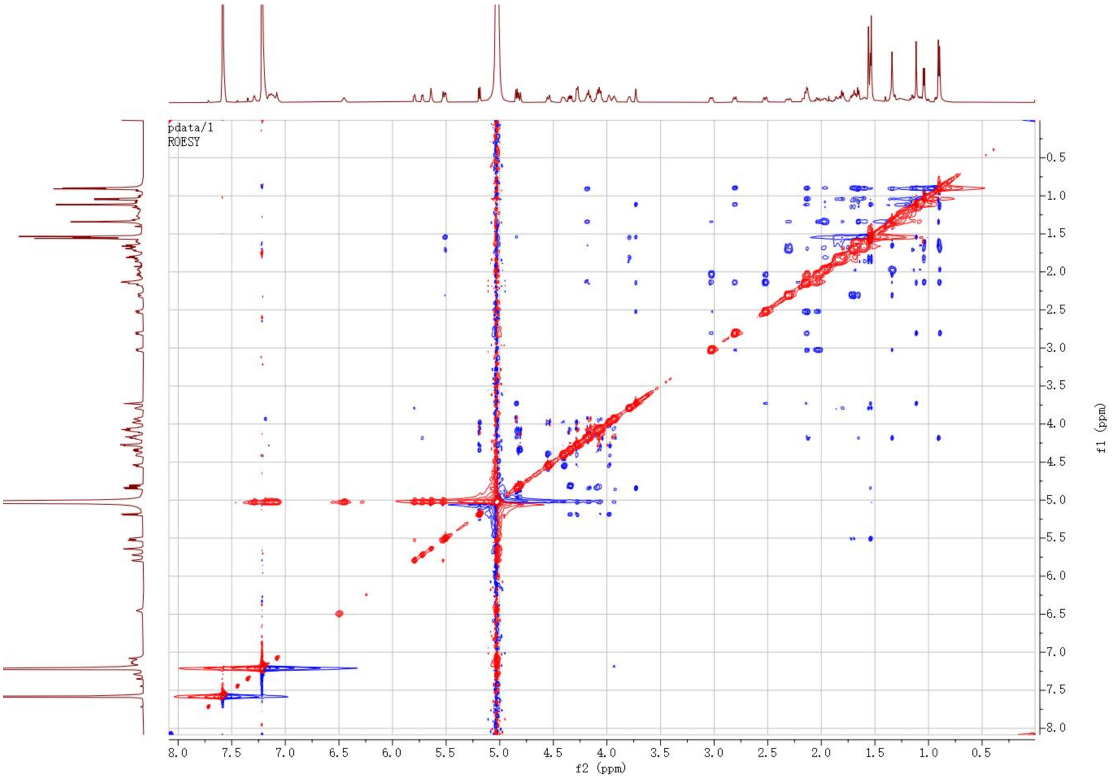

HRESIMS [M-H]$^-$

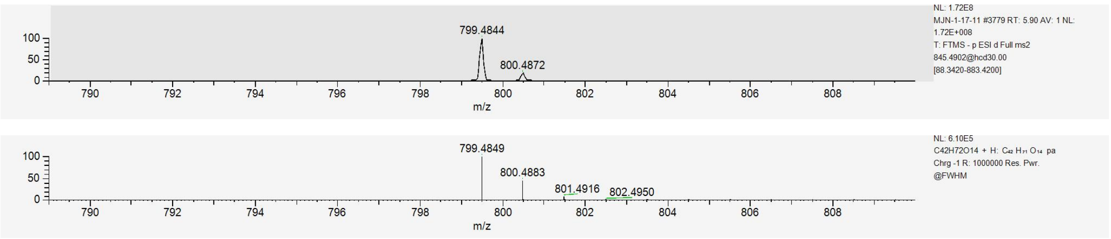

Compound **13**

^{1}H NMR

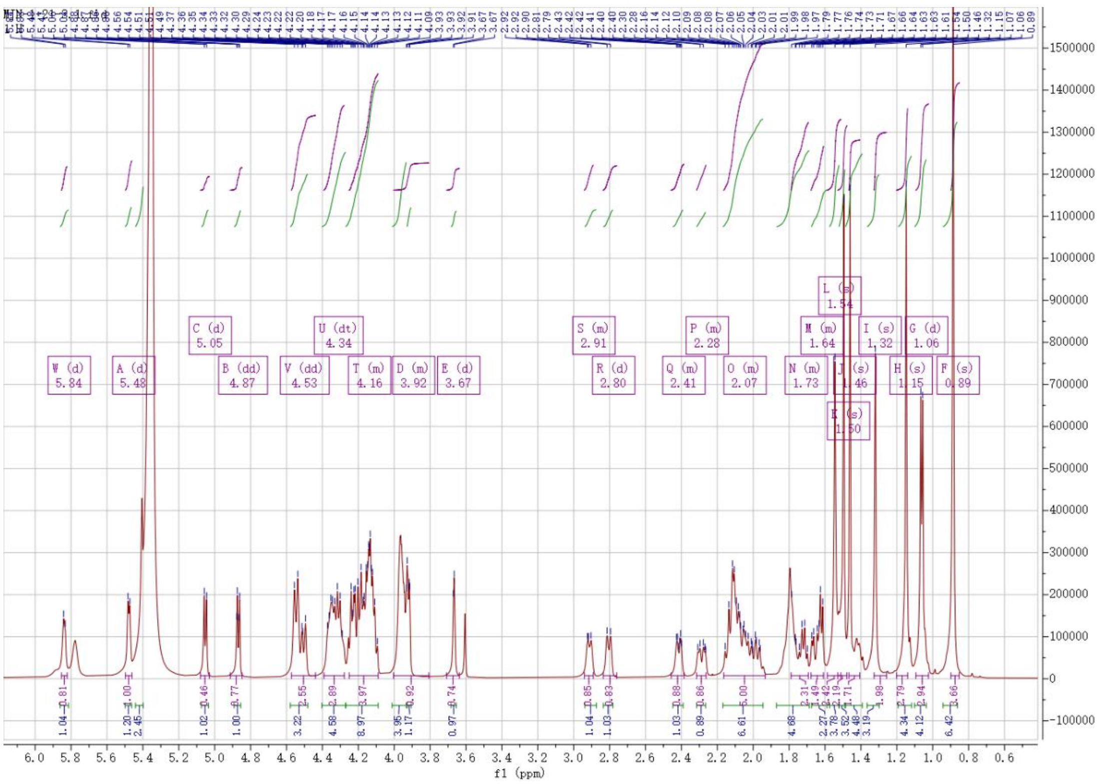

^{13}C NMR

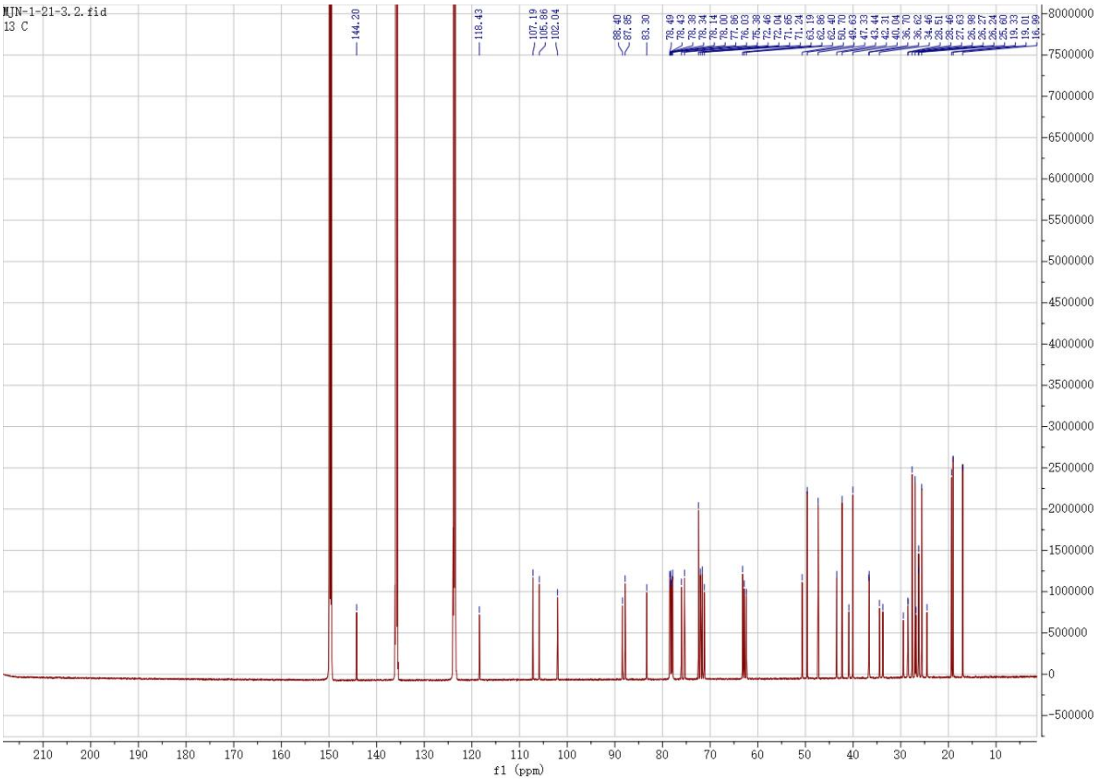

MJN-1-21-3.2.fid
13 C
144.20
118.43
107.19
105.86
102.04
88.40
87.85
83.30
78.49
78.43
78.38
78.34
78.14
78.00
77.86
76.03
75.38
72.46
72.04
71.65
71.24
63.19
62.86
62.40
50.70
49.63
47.33
43.44
42.31
40.04
36.70
36.62
34.46
28.51
28.46
27.63
26.98
26.27
26.24
25.60
19.33
19.01
16.99
f1 (ppm)

HSQC

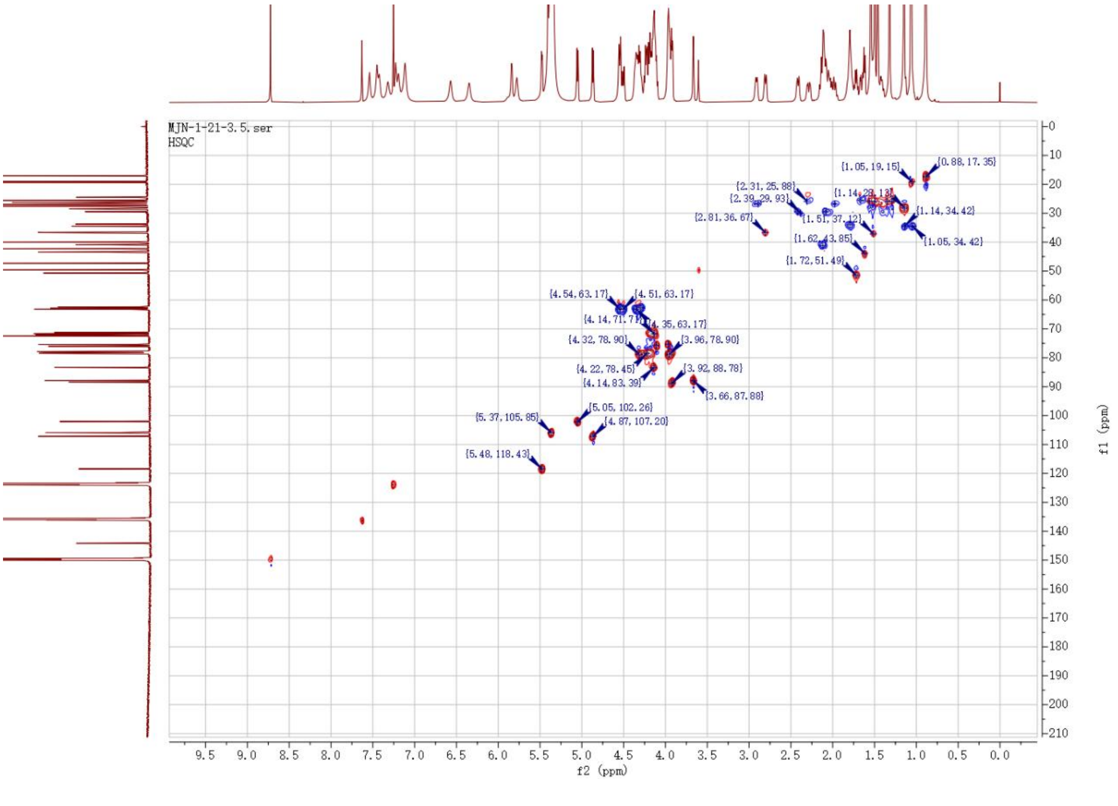

MJN-1-21-3.5.ser
HSQC
{1.05, 19.15}
{0.88, 17.35}
{2.31, 25.88}
{2.39, 29.93}
{1.14, 34.42}
{2.81, 36.67}
{1.51, 37.12}
{1.62, 43.85}
{1.05, 34.42}
{1.72, 51.49}
{4.54, 63.17}
{4.51, 63.17}
{4.35, 63.17}
{3.96, 78.90}
{4.32, 78.90}
{4.22, 78.45}
{3.92, 88.78}
{4.14, 83.39}
{3.66, 87.88}
{5.05, 102.26}
{5.37, 105.85}
{4.87, 107.20}
{5.48, 118.43}
f2 (ppm)
f1 (ppm)

^{1}H-^{1}H COSY

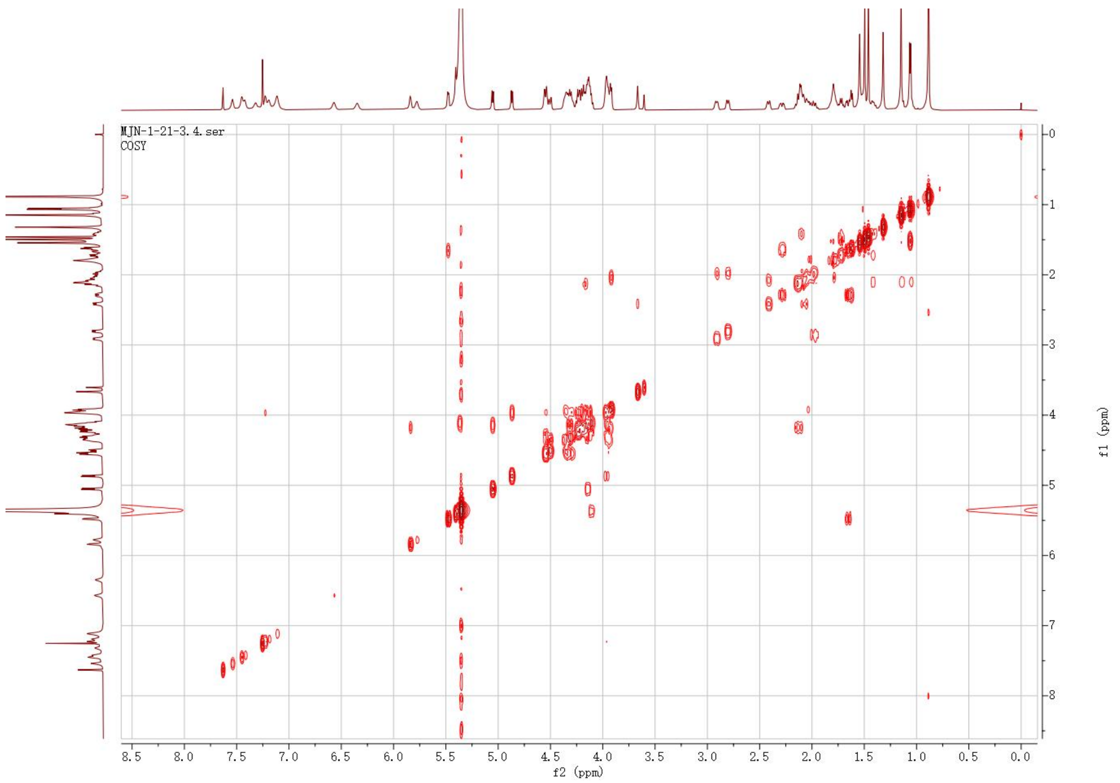

HMBC

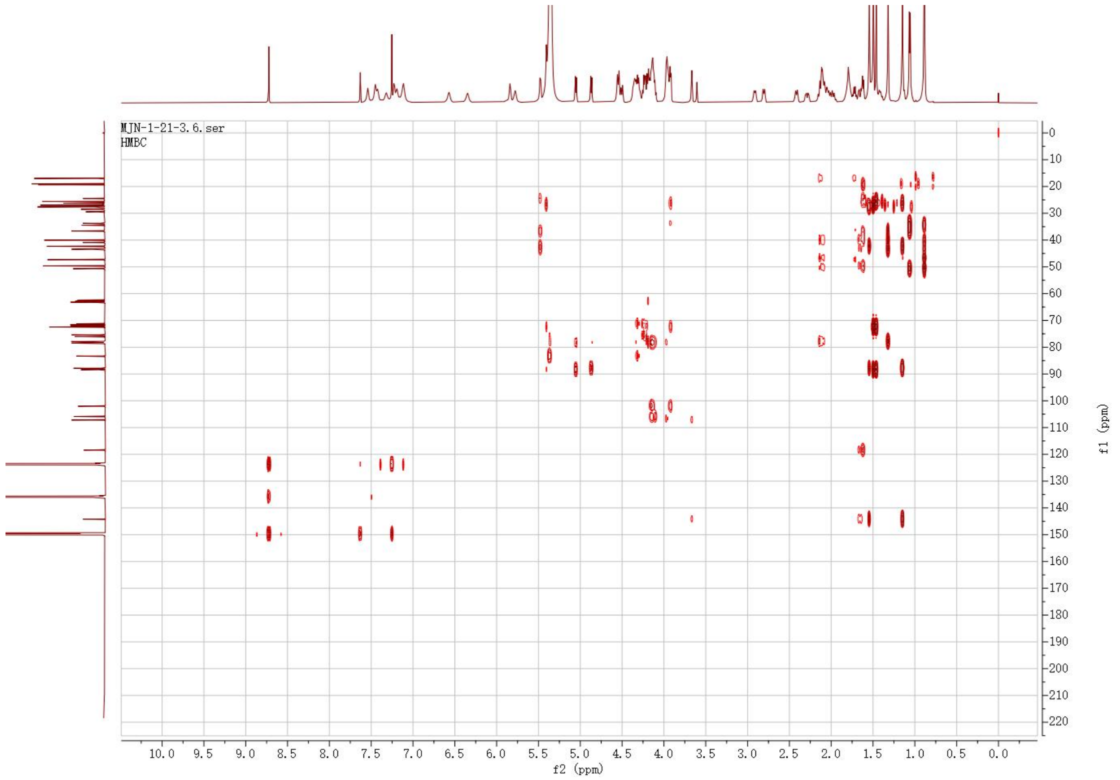

NOESY

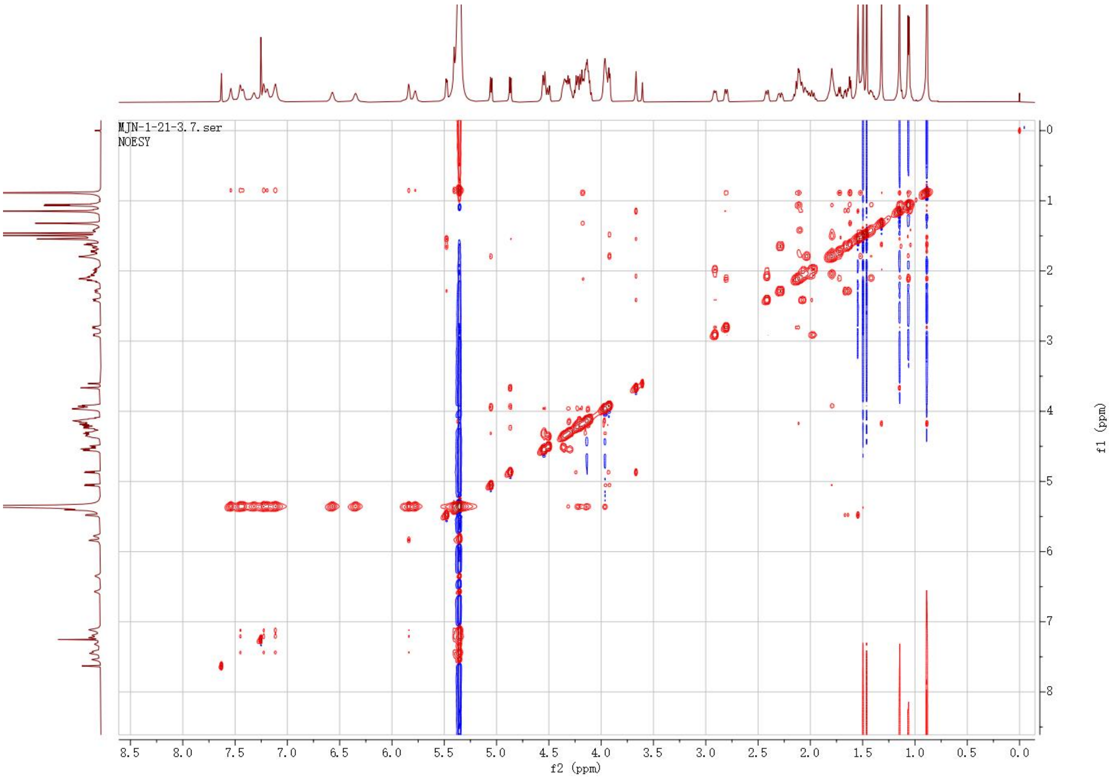

HRESIMS [M+Na]+

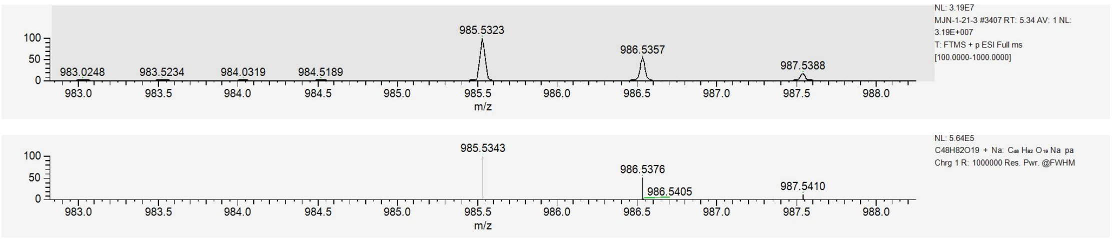

Compound **14**

^{1}H-NMR

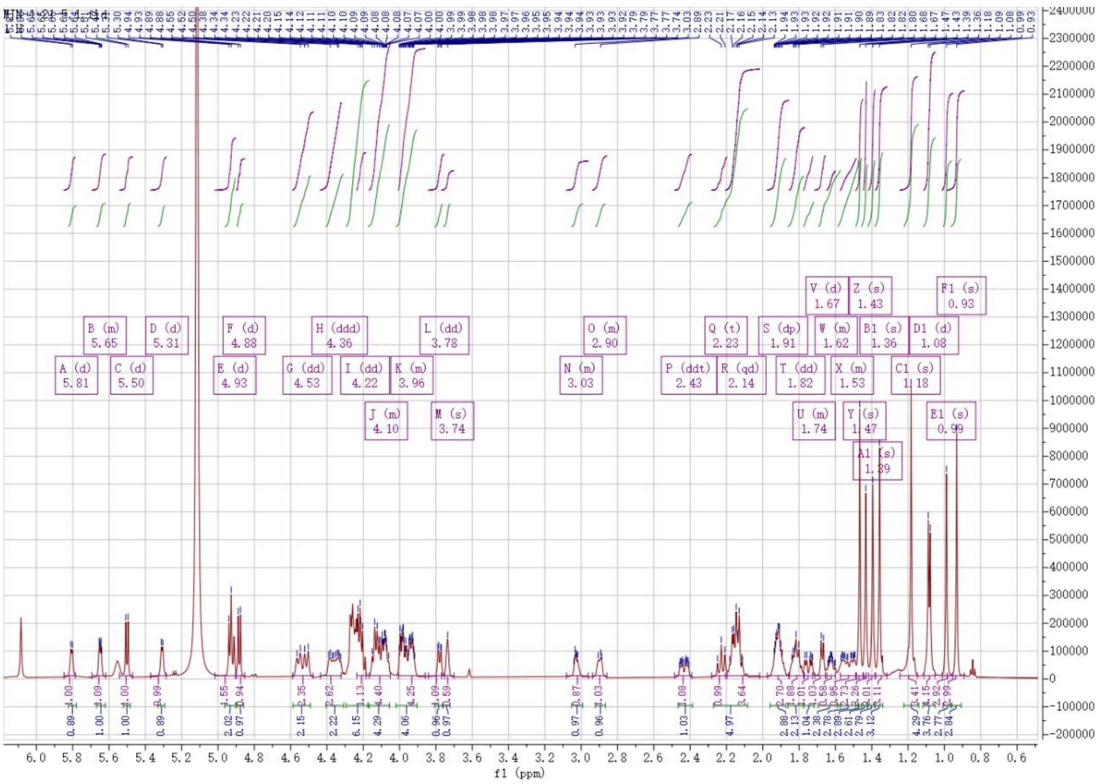

^{13}C NMR

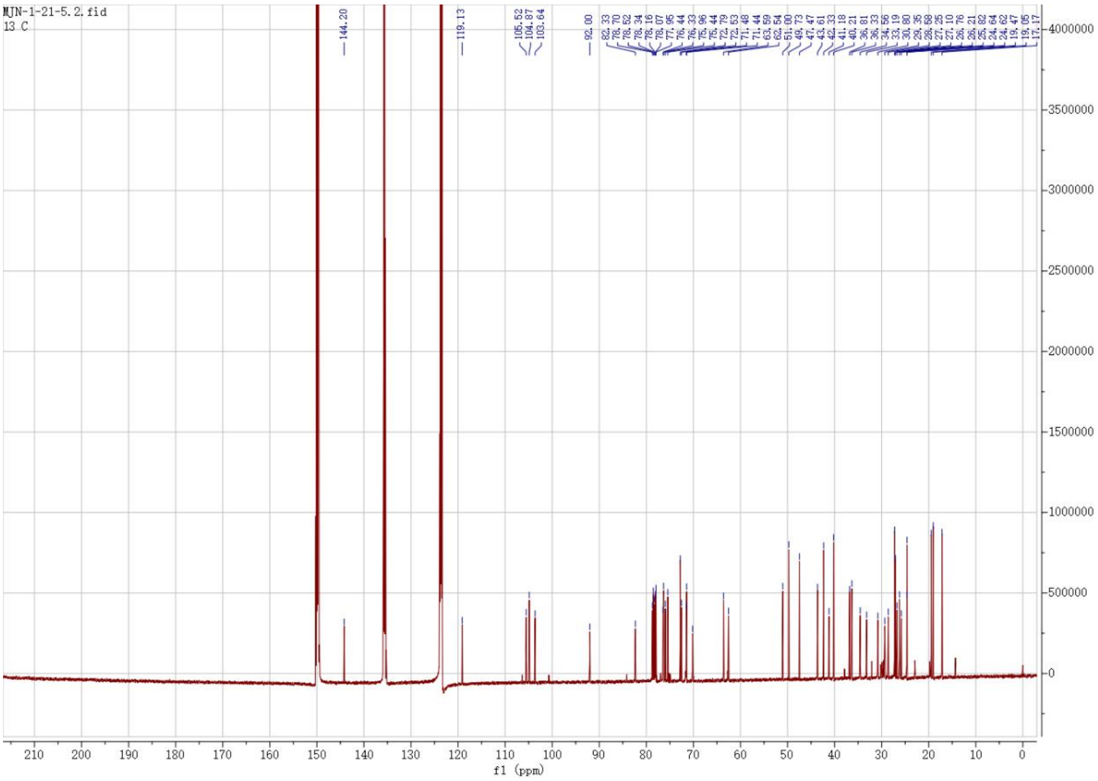

HSQC

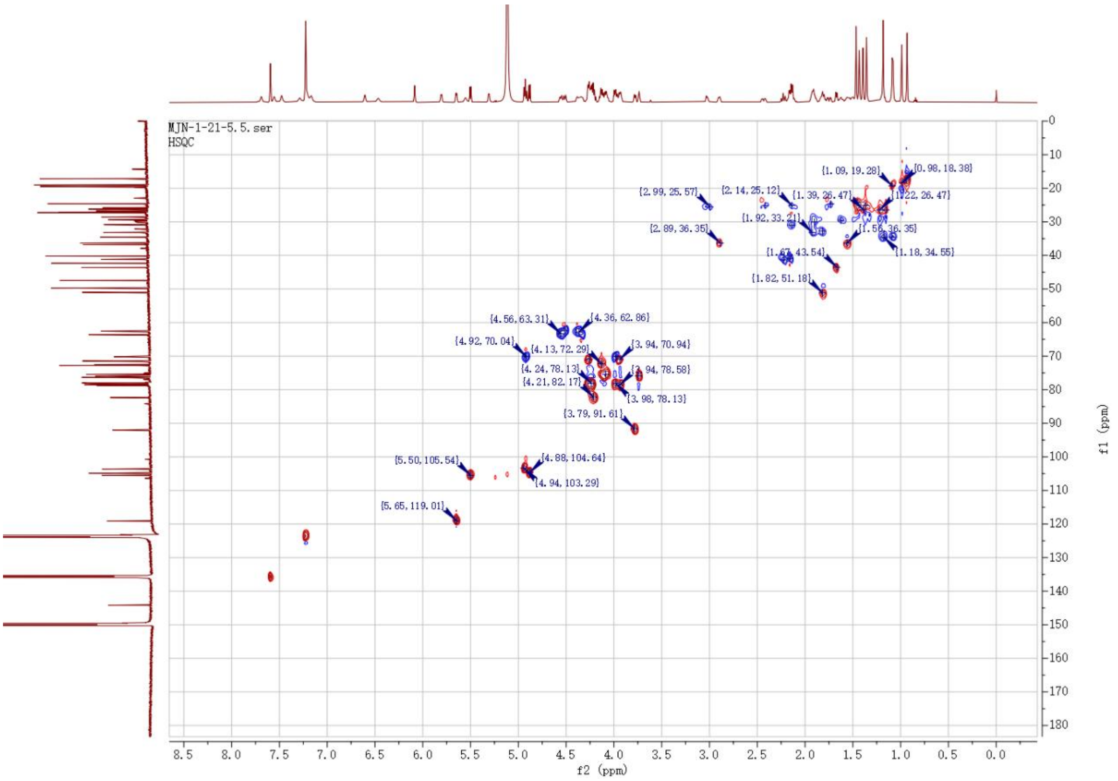

^{1}H-^{1}H COSY

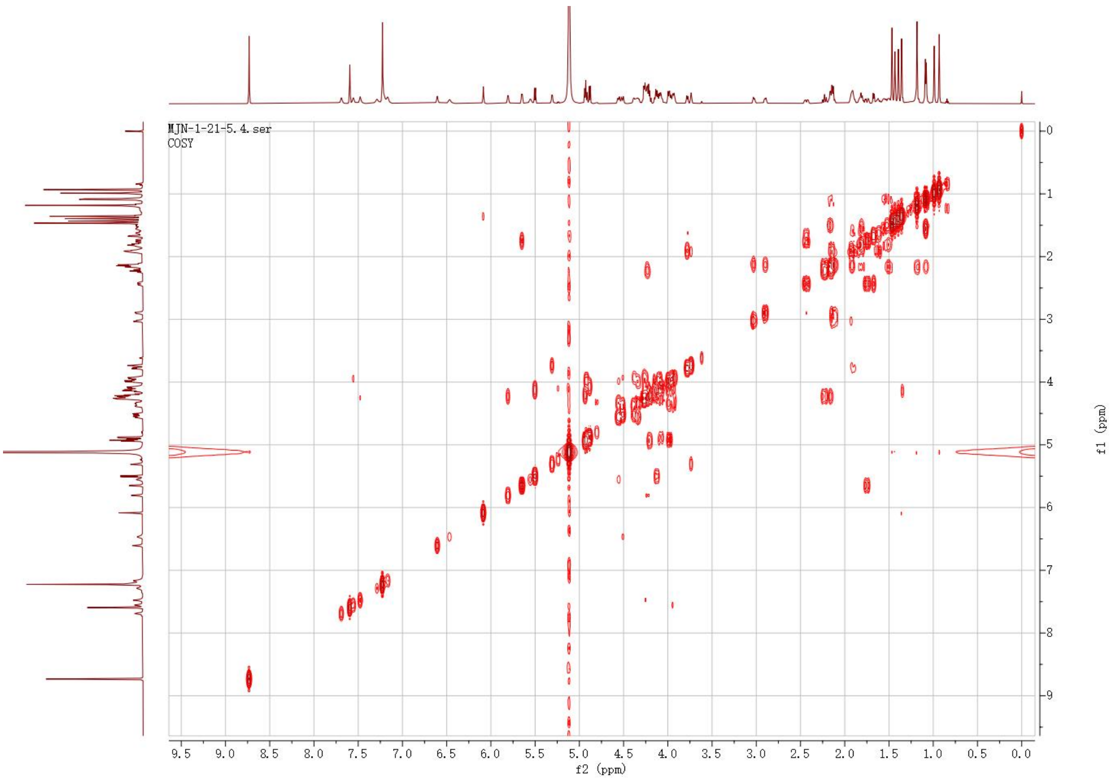
MJN-1-21-5.4.ser
COSY
f1 (ppm)
f2 (ppm)

HMBC

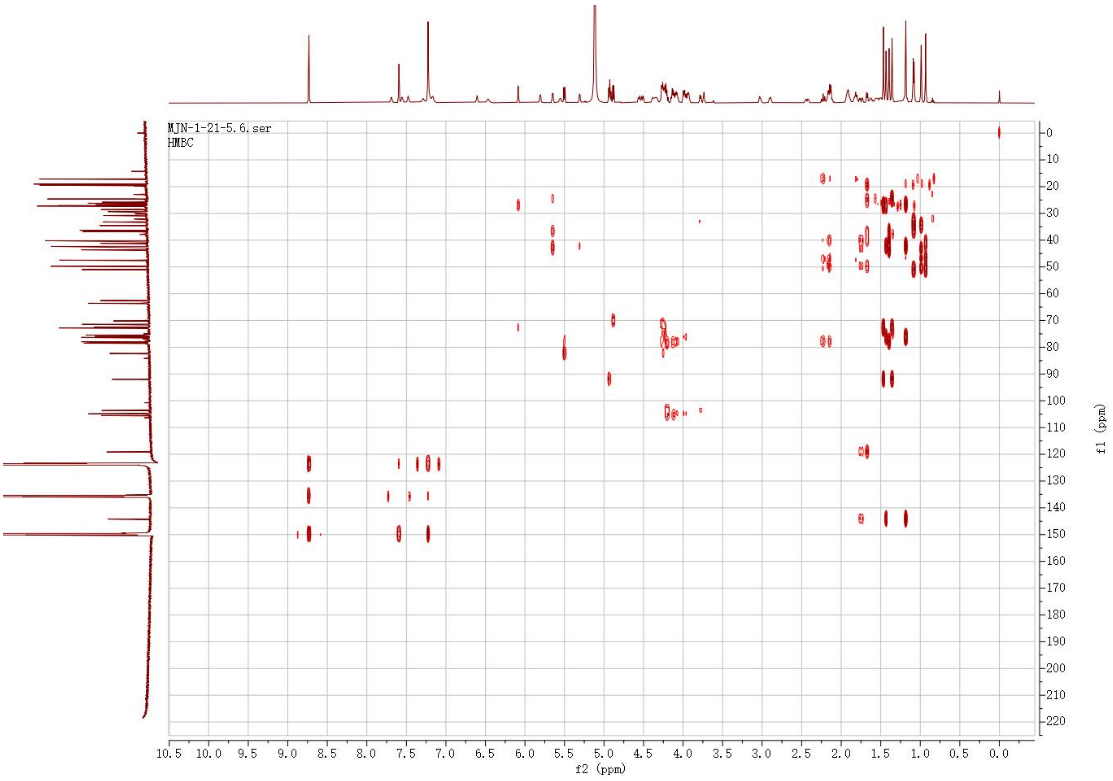
MJN-1-21-5.6.ser
HMBC
f1 (ppm)
f2 (ppm)

NOESY

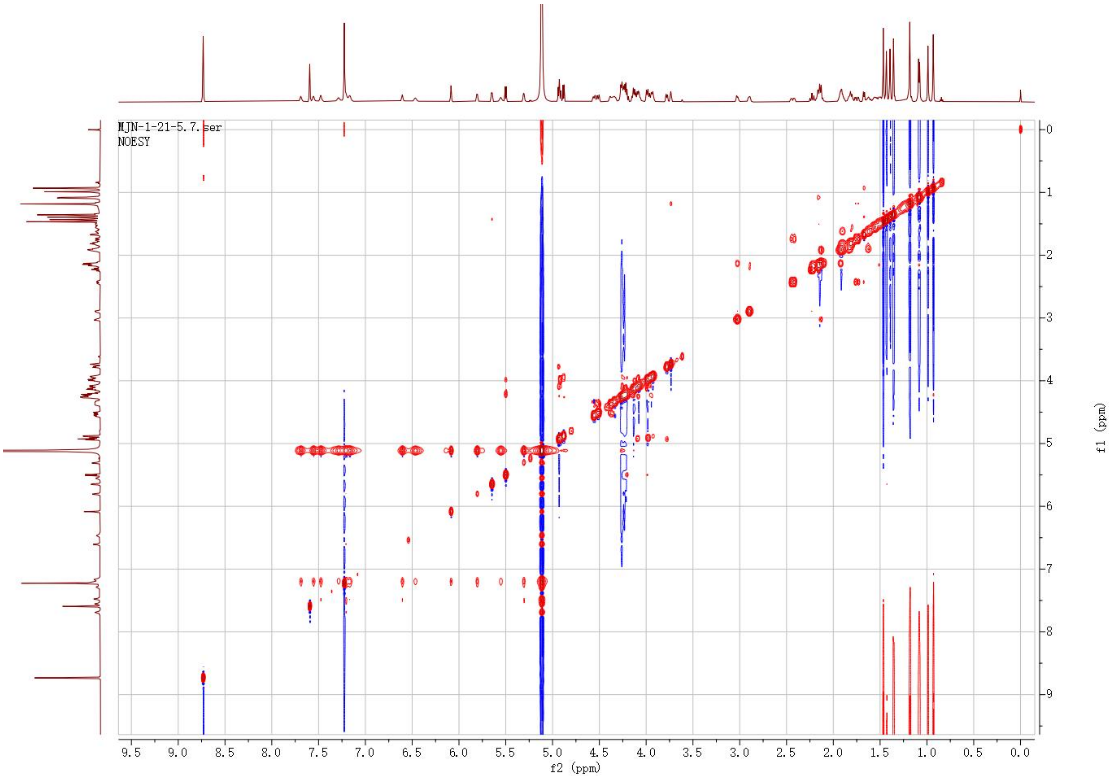

HRESIMS [M+H]$^{+}$

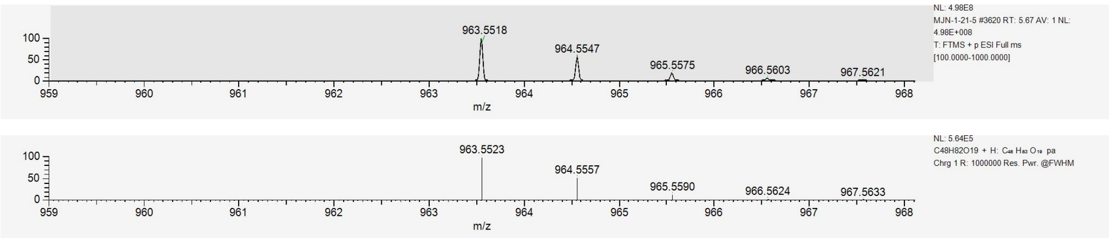

Compound **15**

^{1}H NMR

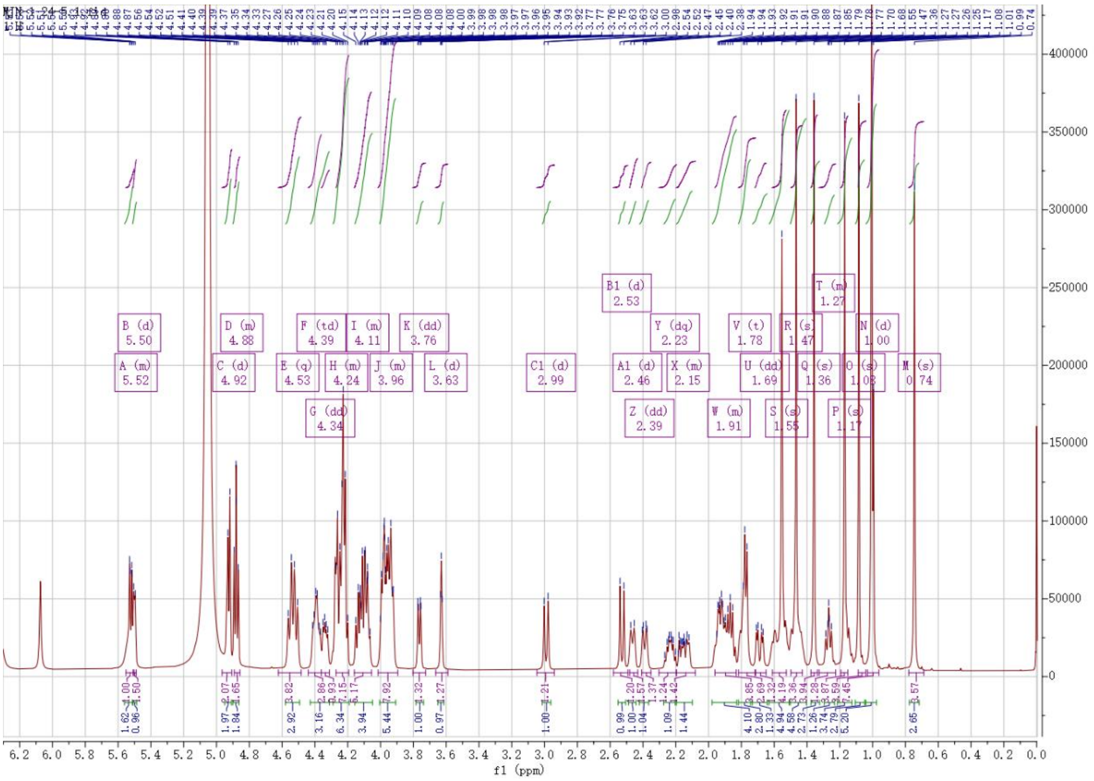

^{13}C NMR

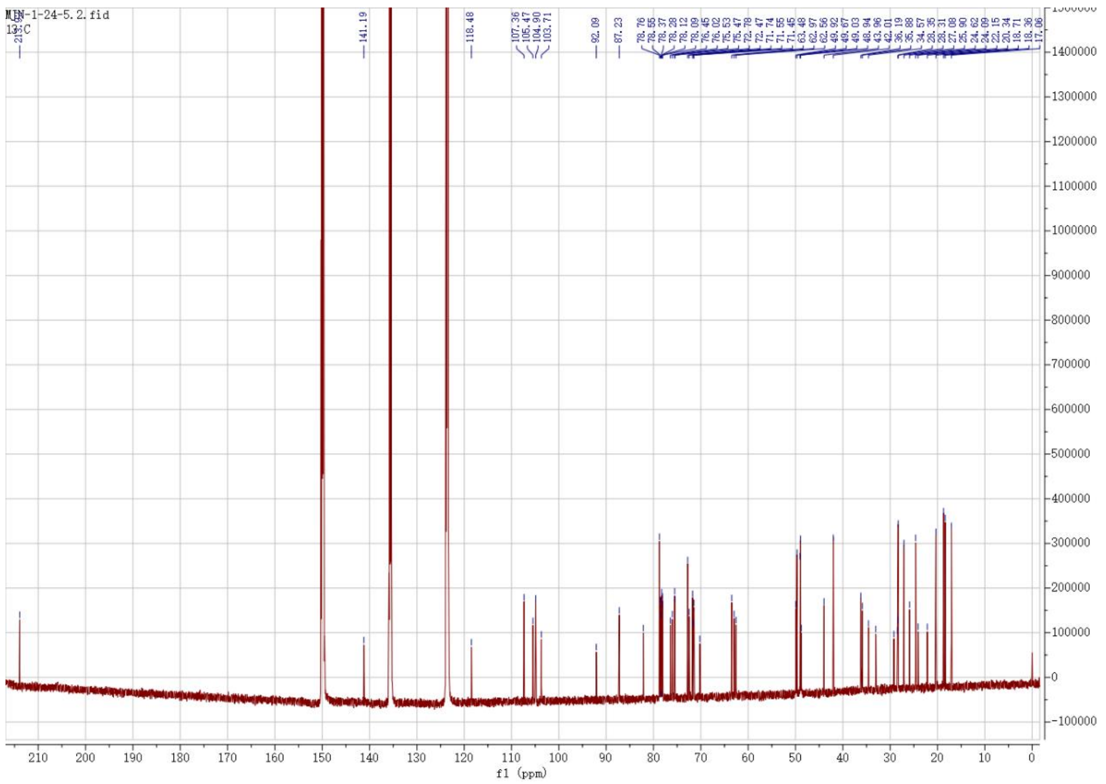

HSQC

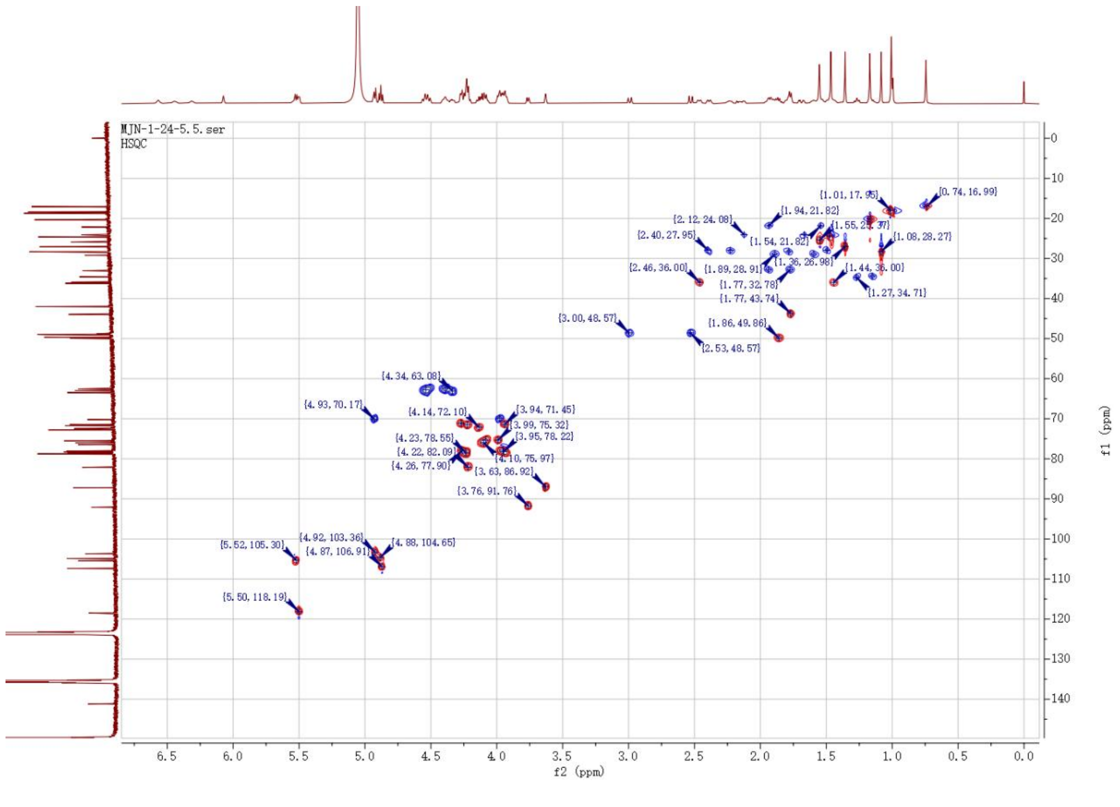

^{1}H-^{1}H COSY

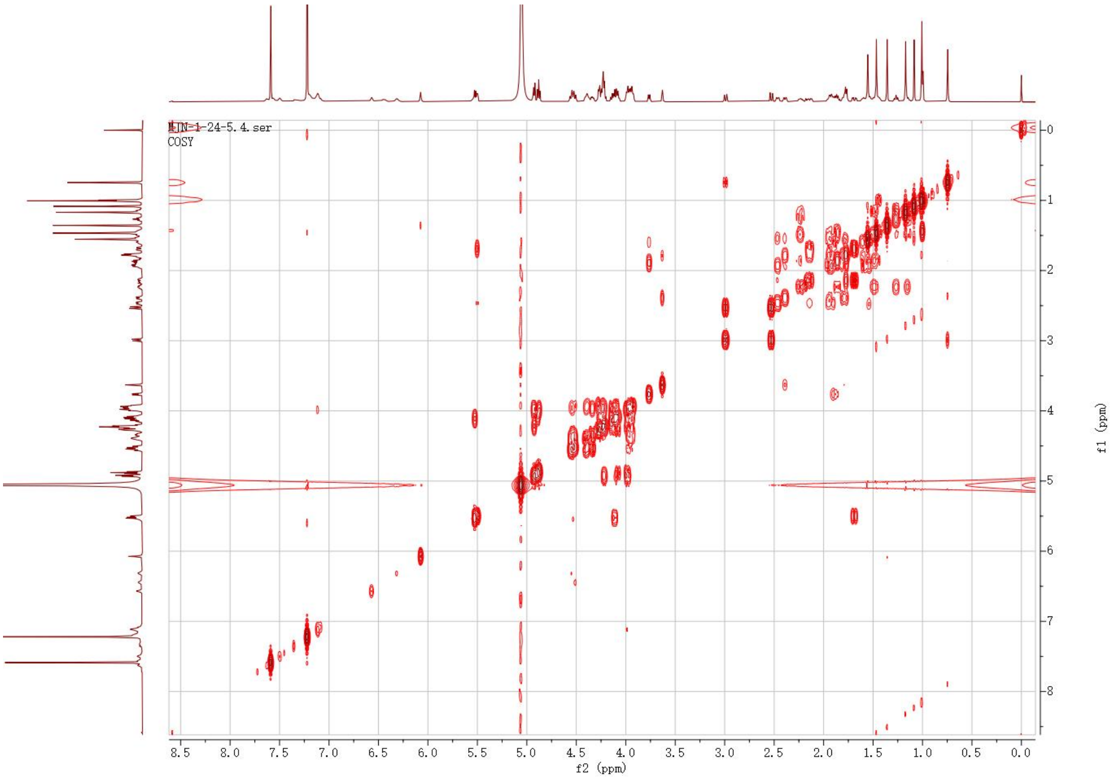

HMBC

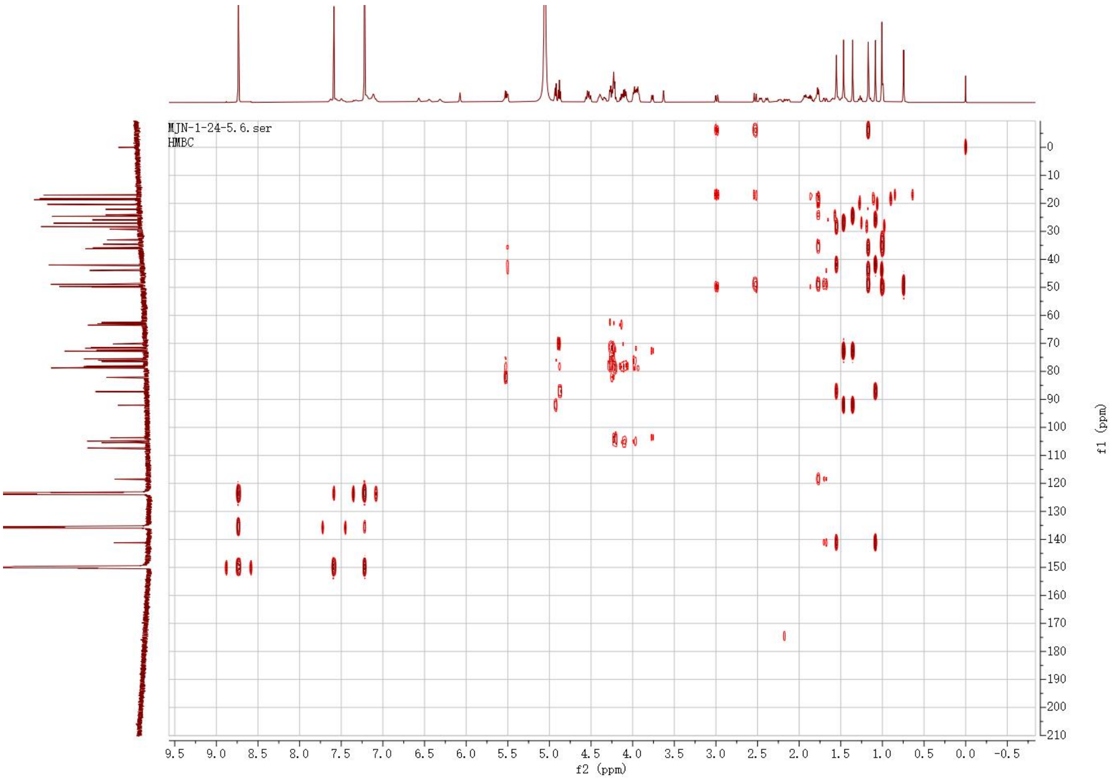

NOESY

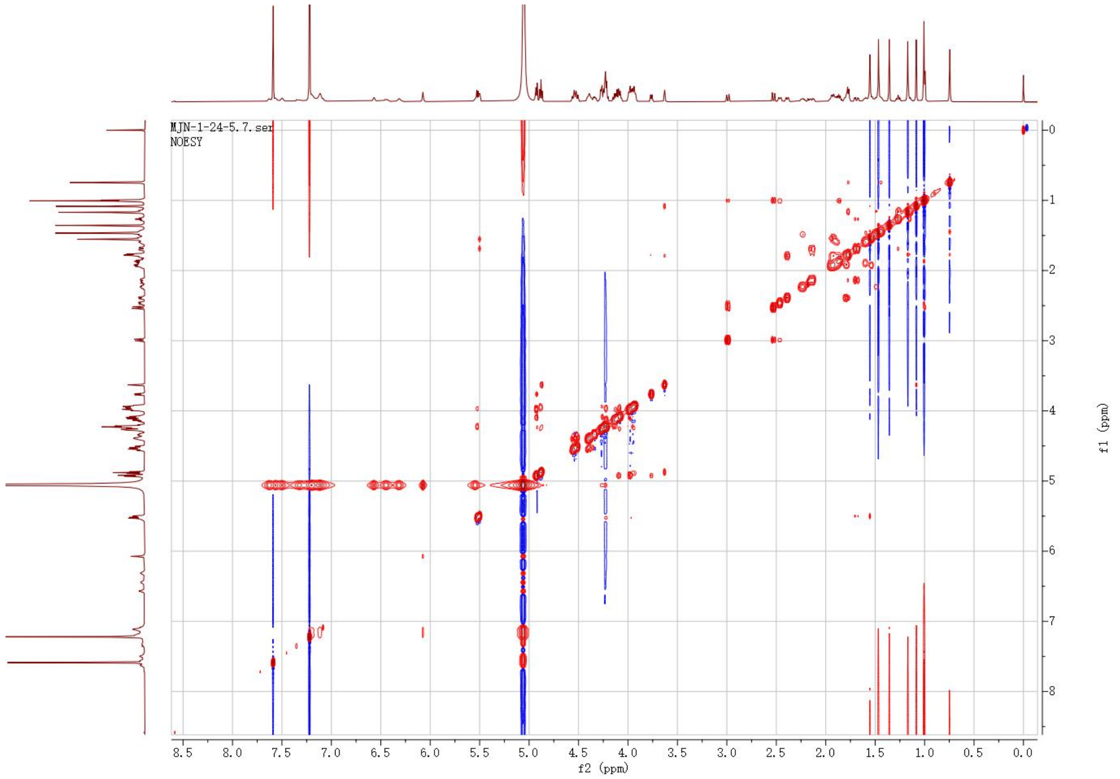

HRESIMS [M+Na]$^+$

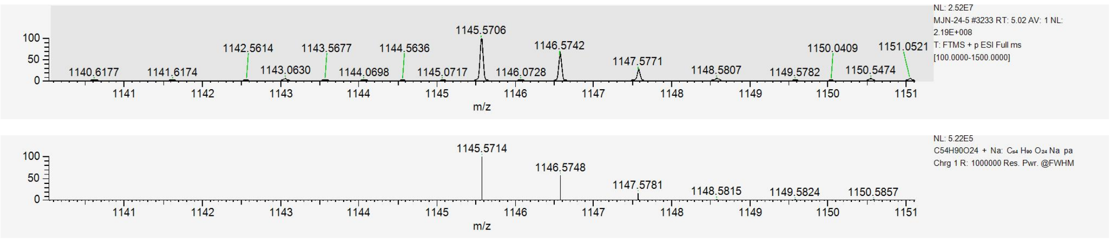

Supplement: Supplementary file 1 [file molecules-30-03983-s001.zip › molecules-3851765-supplementary.pdf]
